# Supplementary material for: The genome of pest Rhynchophorus ferrugineus reveals gene families important at the plant-beetle interface
Source: Commun Biol. 2020 Jun 24;3:323. doi: 10.1038/s42003-020-1060-8 (PMC7314810; doi:10.1038/s42003-020-1060-8)
Supplement: Supplementary file 1 — Supplementary Information [file 42003_2020_1060_MOESM1_ESM.pdf]

The genome of the red palm weevil pest (*Rhynchophorus ferrugineus*) reveals key gene families functioning at the plant-beetle interface

Supplementary Figures

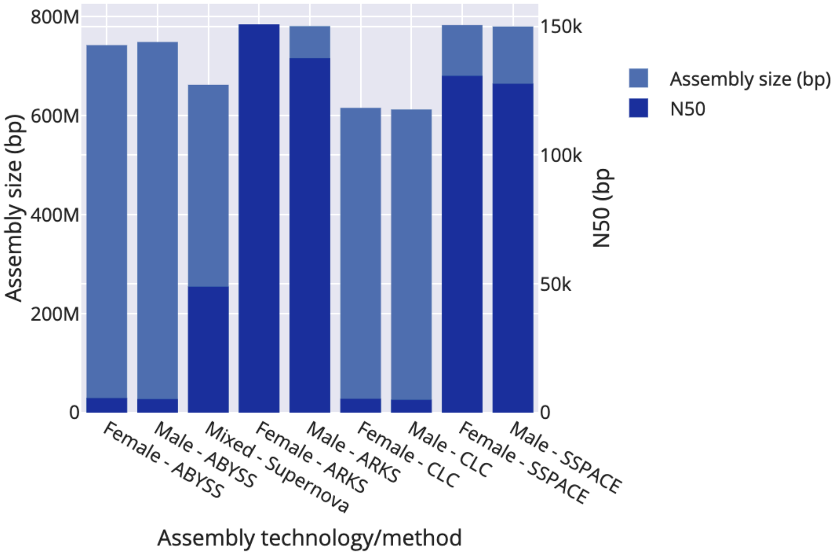

Supplementary Fig.1 Different approaches using CLC and SSPACE softwares to improve assembly statistics

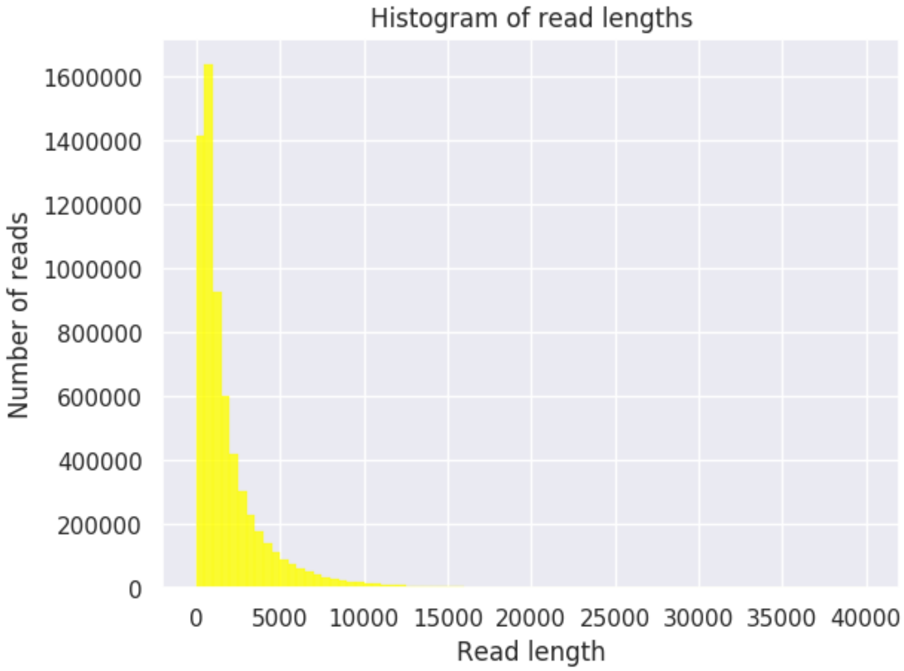

Supplementary Fig.2 Distribution of reads from the oxford Nanopore data

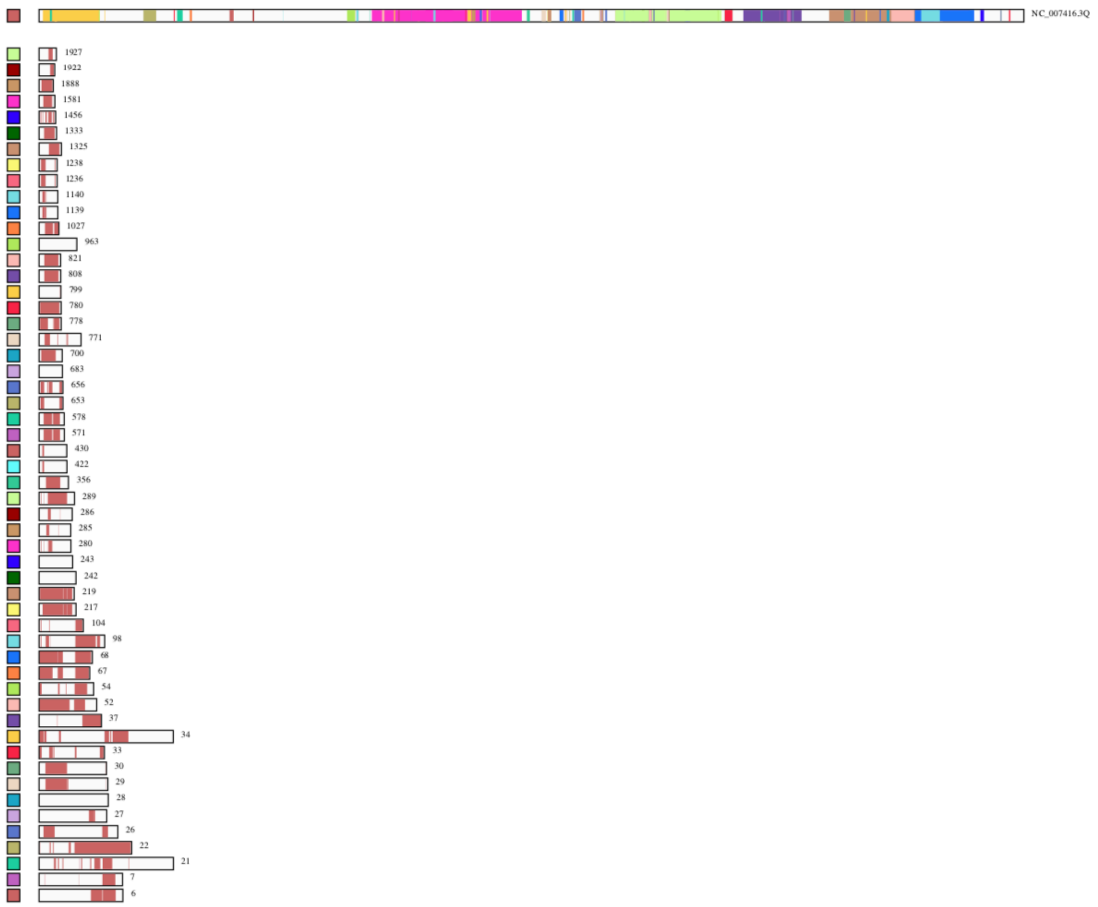

Supplementary Fig. 3 The mapping of 54 scaffolds of the red palm weevil to red flour beetle is highlighted in different colors. The top is the X chromosome of the red flour beetle (*T. castaneum*).

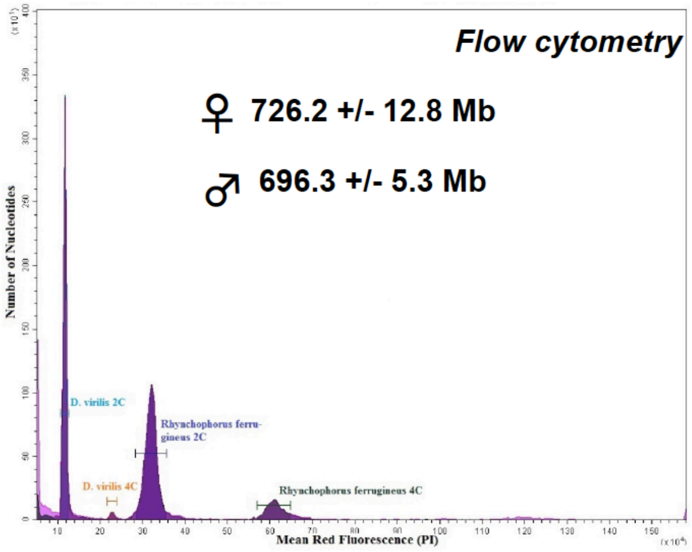

Supplementary Fig. 4 Flow cytometry results for male and female red palm weevil

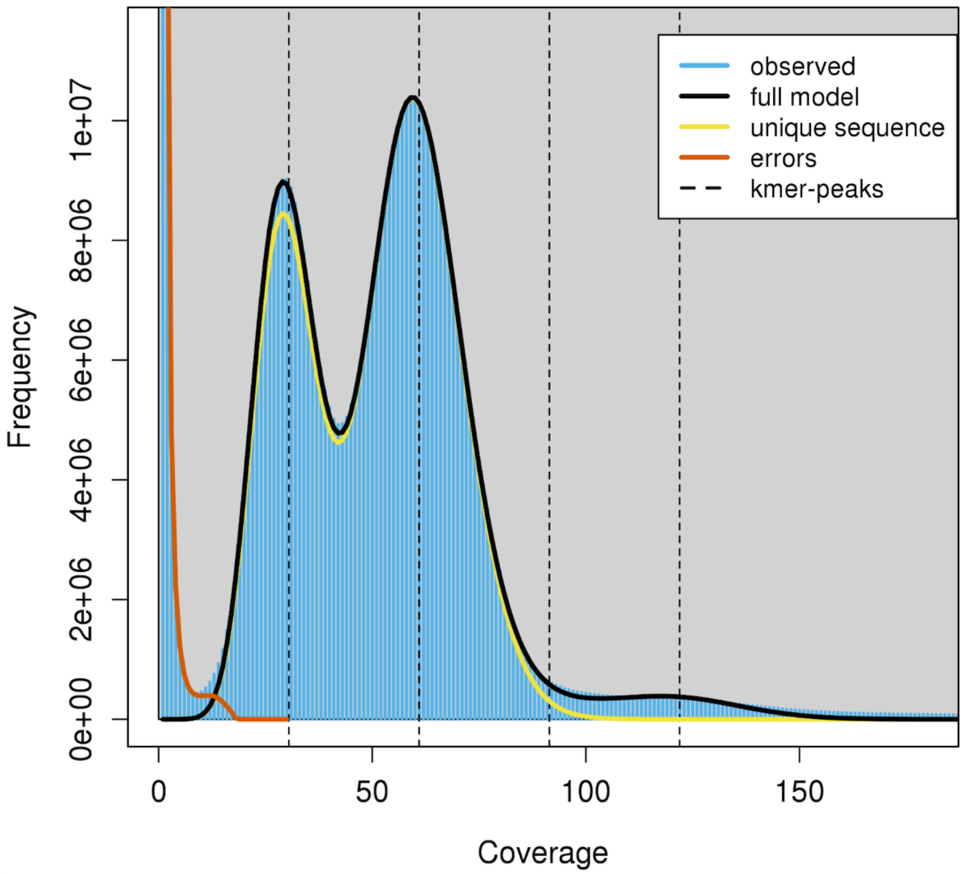

Supplementary Fig. 5 Kmer plot for genome size estimation using jellyfish software in GenomeScope

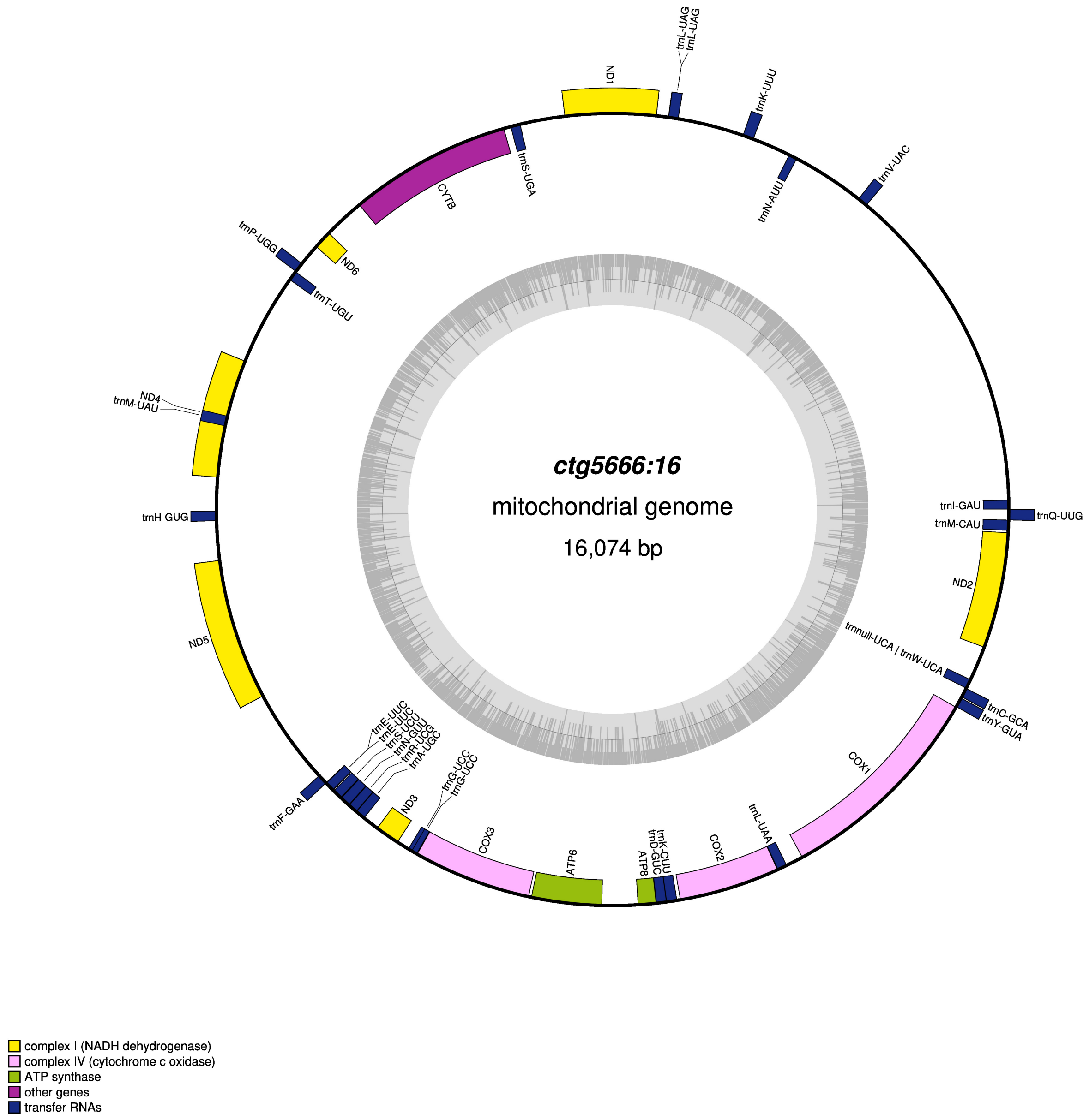

Supplementary Fig. 6 The complete mitochondrial genome assembly of the red palm weevil (*R. ferrugineus)*

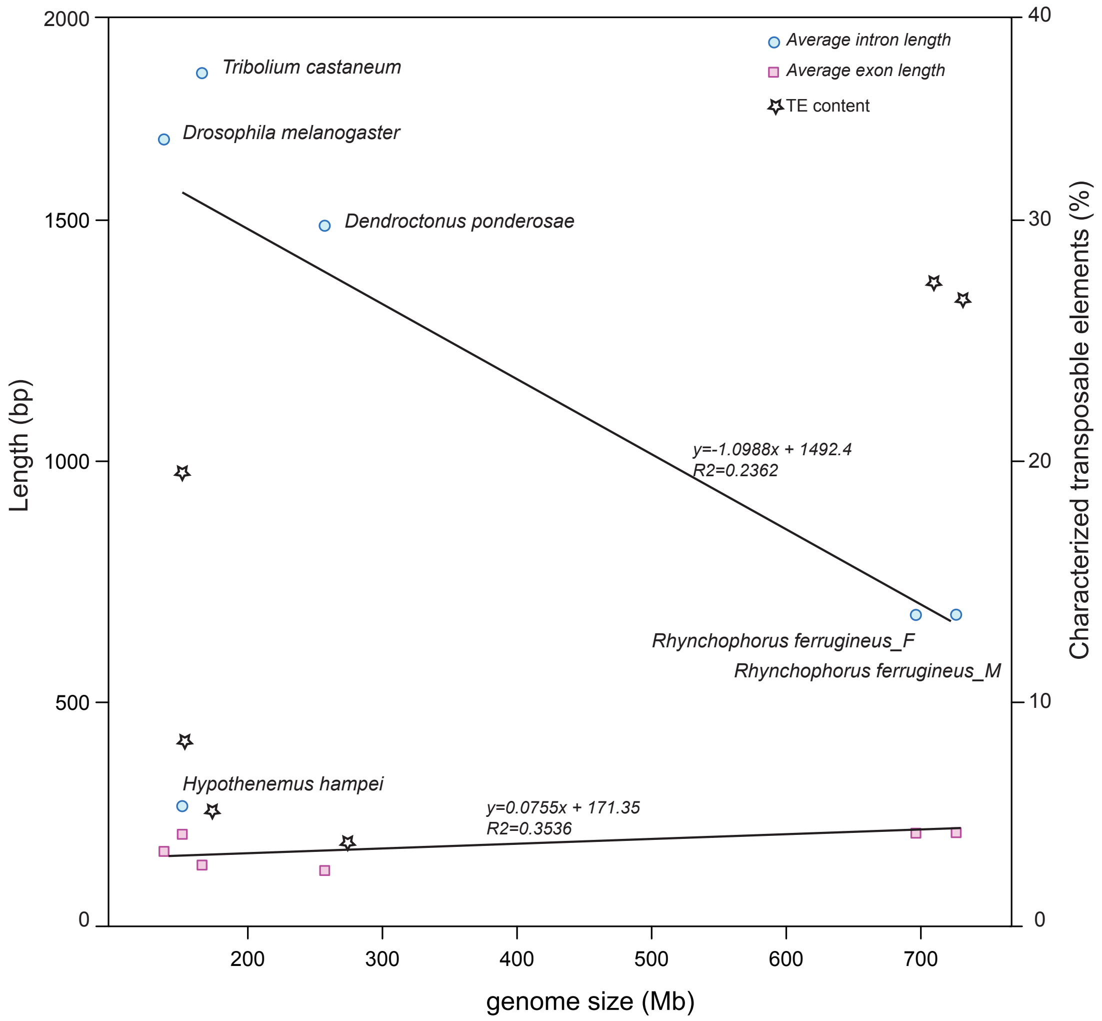

Supplementary Fig. 7 Correlation of transposable elements with intron, exon length and genome size in five insects studied.

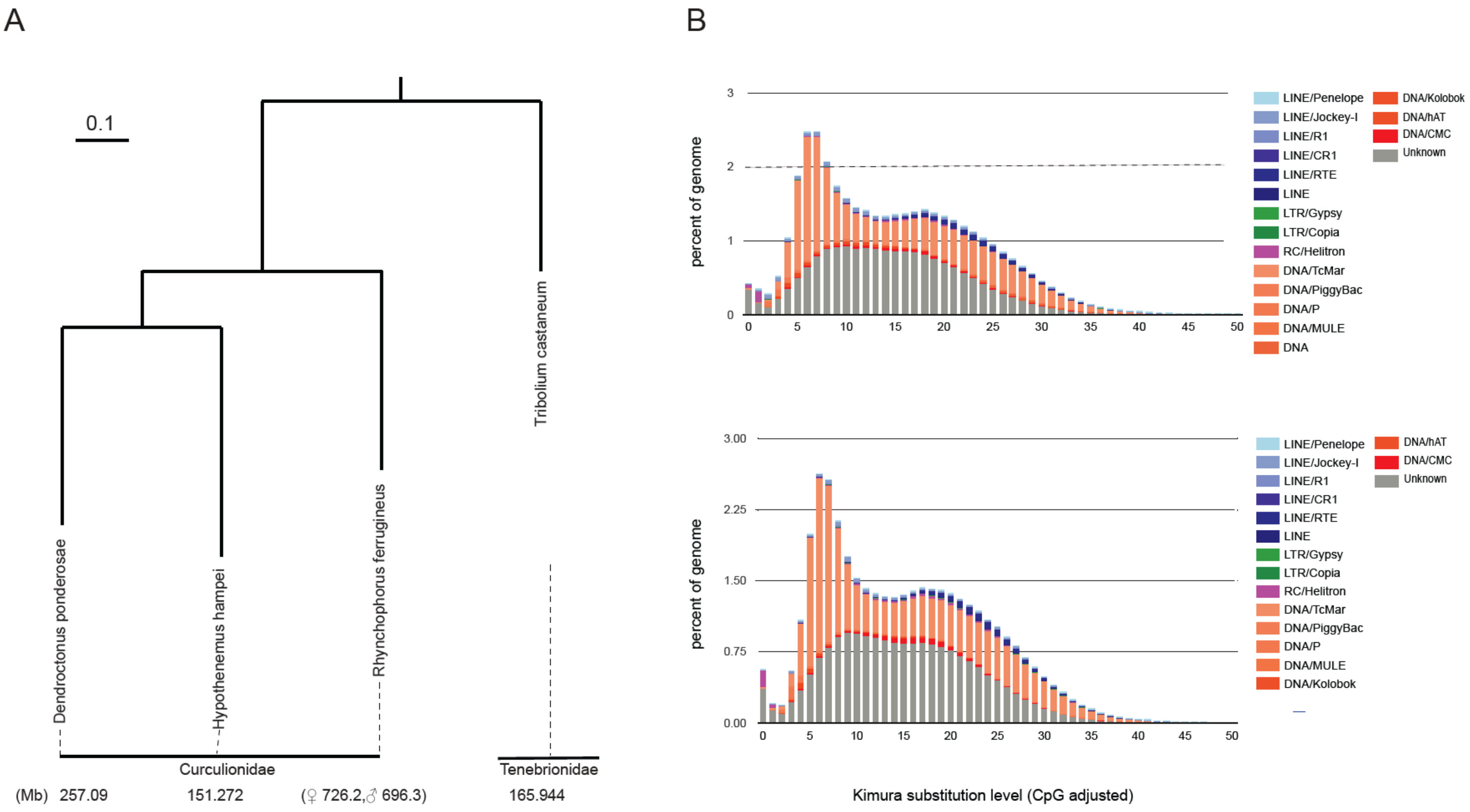

Supplementary Fig. 8 Repeat landscape in red palm weevil (*R. ferrugineus)* male and female (B) and with other insects in this study (A).

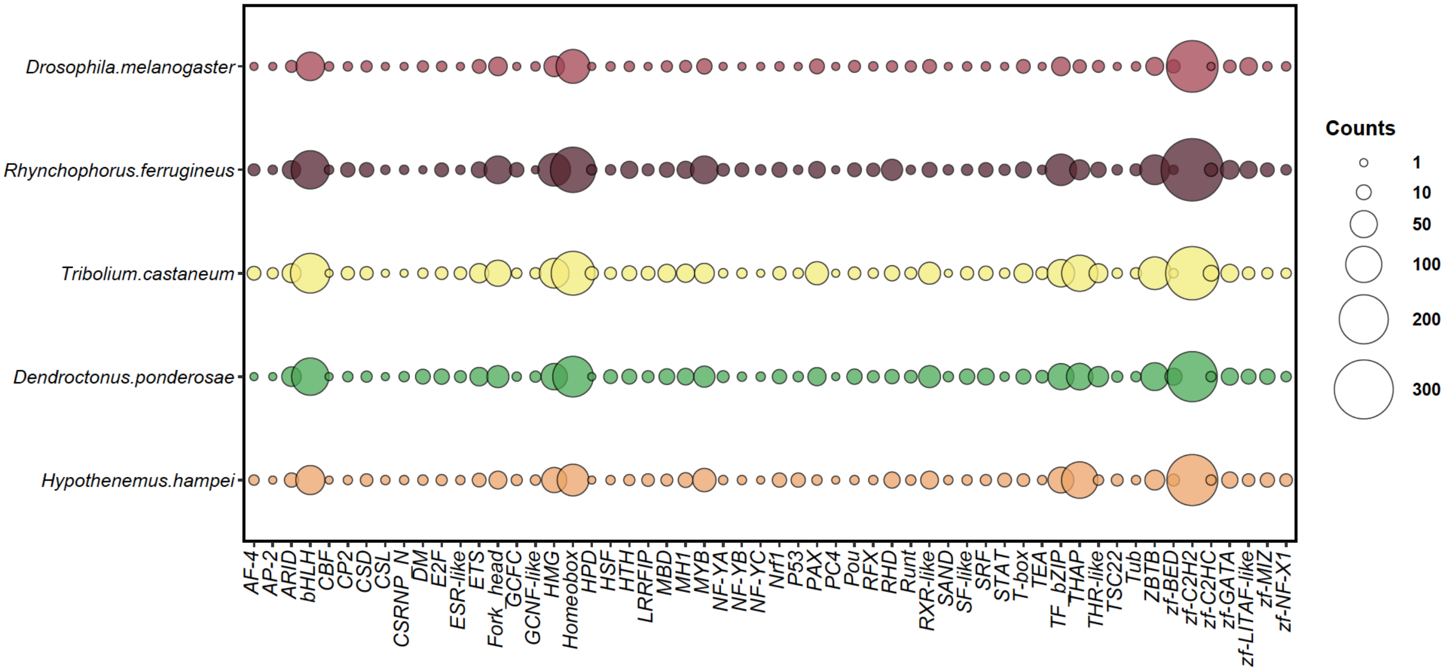

Supplementary Fig. 9 Comparative analysis of transcription factors (TFs) in the five species studied. The x-axis depicts the different families predicted and the y-axis the different species. The legend is the counts representing the numbers in terms of the size of the bubble.

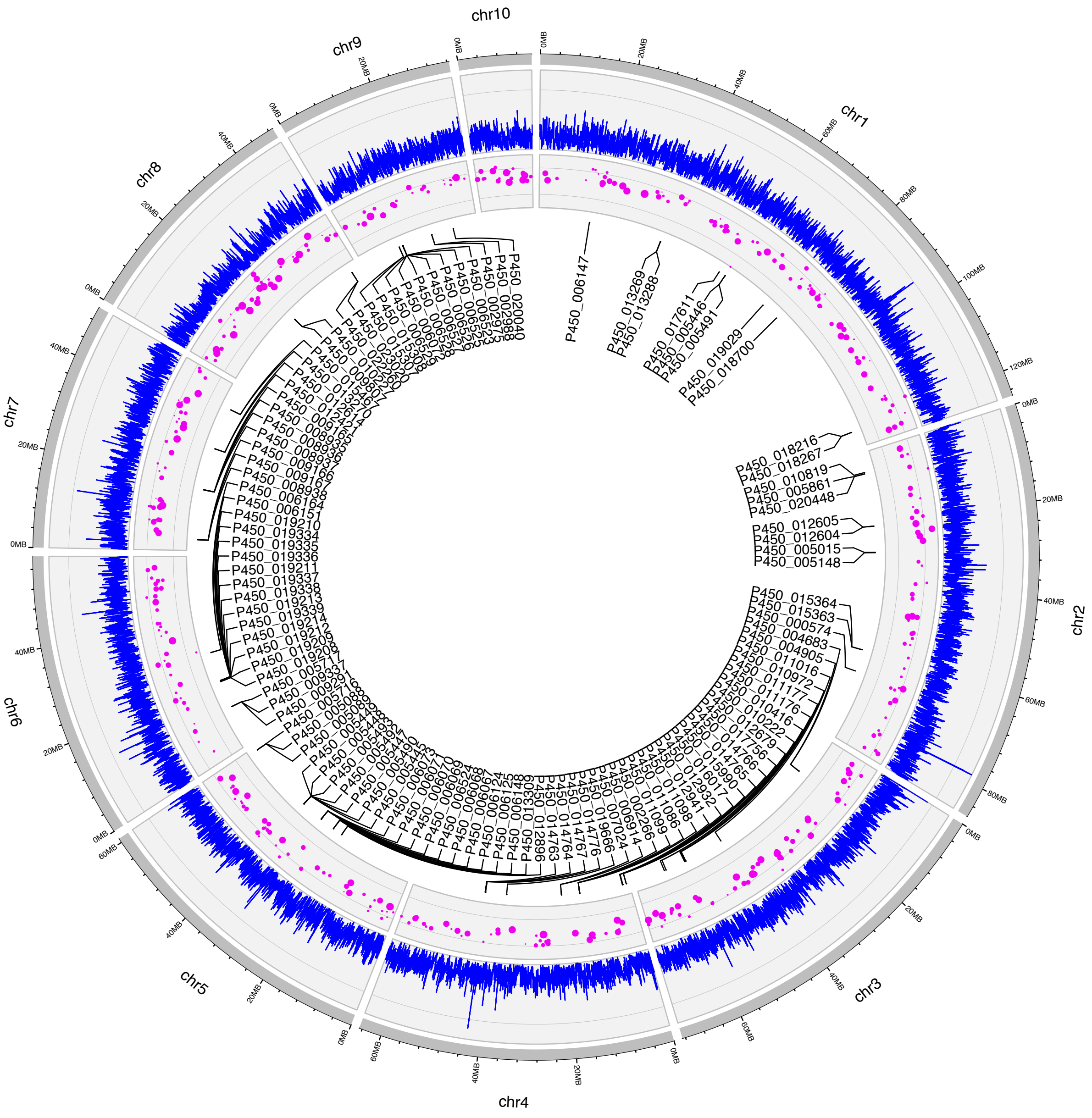

Supplementary Fig. 10 Circos plot showing diversity Pi (π) in the outer track, followed by duplication and location of tandem duplication of P450 gene family on pseudochromosomes.

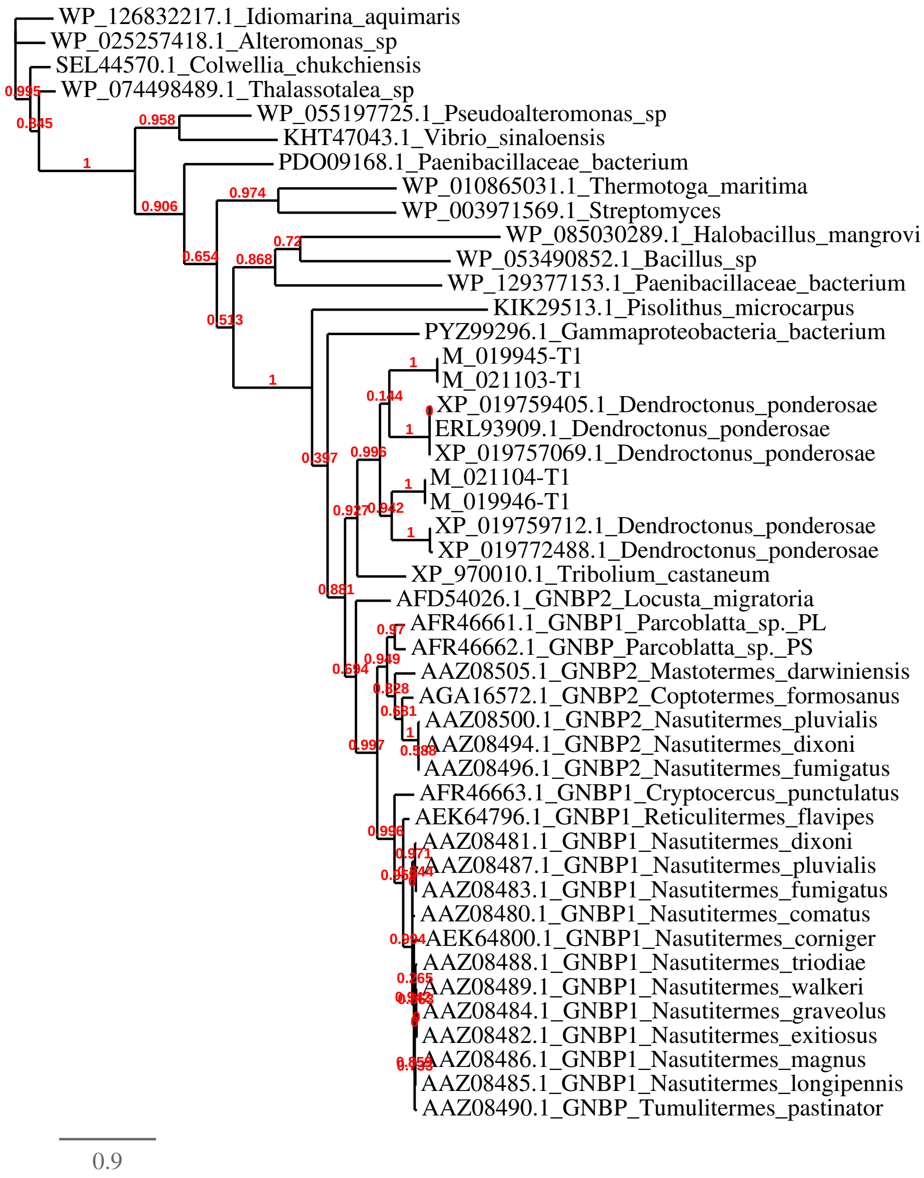

Supplementary Fig. 11 Bootstrapped Phylogeny of the Glycosyl hydrolase 16 with other insects and microorganisms. Red value are the branch and node support.

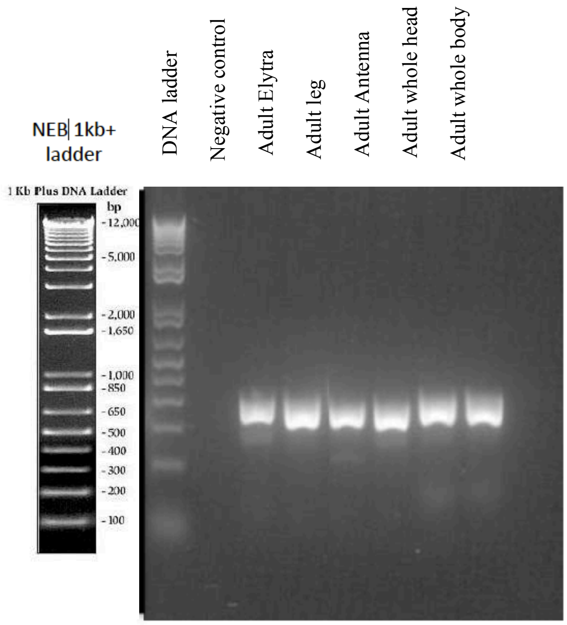

Left primer: CTCGCCAACAAAGACATGAA
Right primer: GTTGTGGTTCCATGTTGCTC

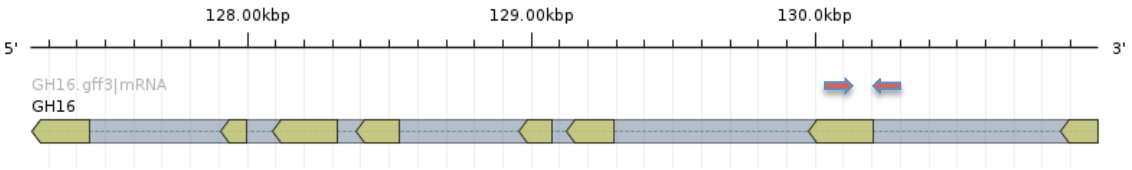

Supplementary Fig. 12 The top part is a gel electrophoresis of a fragment of one GH16 (primers included) to validate the presence in the genome and rule out gut microbial contamination. The lower part is the structure of the GH16 and the location of the primers.

Supplementary Tables

GO ID	Namespace	Name	Count	p-value
GO:0006310	biological_process	DNA recombination	6	2.0771860895782084e-06
GO:0031098	biological_process	stress-activated protein kinase signaling cascade	7	0.00016010234381436095
GO:0031297	biological_process	replication fork processing	4	0.00028241147532428757

Supplementary Table 1 GO enrichment of orphan genes in red palm weevil (*R. ferrugineus)*

Description	Locus
probable chitinase 10	LOC105256951 (GH18)
cytochrome P450 4c3-like	LOC109535116
putative odorant-binding protein 23 mRNA	OBP
UDP-glucuronosyltransferase-like	LOC109544879
cytochrome P450 9e2-like	LOC109540845
pectinesterase B-like	LOC109533512
protein yellow-like	LOC109542861
protein retinal degeneration B	LOC109542972
**gamma-aminobutyric acid receptor subunit beta**	**LOC109542303 (Rdl)**
**beta-1,3-glucan-binding protein-like**	**LOC109534426 (GH16)**

Supplementary Table 2 Frequency of changes identified in 50 individuals red palm weevil obtained by RNA sequencing (RNAseq) Rdl from adult heads.

Genotype	Number of Individuals	Frequency
WT: Ala^{301}	14	0.28
Mutant: Ser^{301}	30	0.6
Mutant: Thr^{301}	6	0.12

Supplementary Table 3 Summary of some gene families under positive selection.

CAZy family	Pfam	Pfam description	Known activities	Number
**GH1**	PF00232	Glycosyl hydrolase family 1	β-glucosidase, β-galactosidase, 6-phospho-β-glucosidase, β-glucuronidase, others	44
**GH20**	PF00728	Glycosyl hydrolase family 20	β-hexosaminidase, lacto-N-biosidase, β-1,6 Nacetylglucosaminidase	8
**GH3**	PF01915	Glycosyl hydrolase family 3	β-glucosidase	2
**GH30**	PF02055	Glycosyl hydrolase family 30	glucosylceramidase, β-1,6-glucanase, β-xylosidase	6
**GH45**	PF02015	Glycosyl hydrolase family 45	Endoglucanase	4
**GH47**	PF01532	Glycosyl hydrolase family 47	A-mannosidase	16
**GH48**	PF02011	Glycosyl hydrolase family 48	Endoglucanase, chitinase, cellobiohydrolases, endoprocessive cellulases	10
**GH63**	PF16923	Glycosyl hydrolase family 63	Processing α-glucosidase, α-1,3-glucosidase, α-glucosidase	6
**GH79**	PF03662	Glycosyl hydrolase family 79	β-glucuronidase, β-4-O-methyl-glucuronidase, heparanase	1
**GH85**	PF03644	Glycosyl hydrolase family 85	Endo-β-N-acetylglucosaminidase	6
**GH15**	PF00723	Glycosyl hydrolase family 15	glucoamylase, glucodextranase, α,α-trehalase	4
**GH16**	PF00722	Glycosyl hydrolase family 16	endo-1,4-β-galactosidase, endo-1,3-β-glucanase, endo-1,3(4)-β-glucanase, licheninase, β-agarase, others	10
**GH18**	PF00704	Glycosyl hydrolase family 18	Chitinase, endo-β-N-acetylglucosaminidase, others	35
**GH2**	PF02836	Glycosyl hydrolase family 2	β-galactosidase, β-glucuronidase, β-mannosidase, others	9
**GH28**	PF00295	Glycosyl hydrolase family 28	Polygalacturonase, rhamnogalacturonase, others	6
**GH31**	PF01055	Glycosyl hydrolase family 31	α-glucosidase, α-1,3-glucosidase, α-xylosidase	12
**GH32**	PF00251	Glycosyl hydrolase family 1	Levanase, invertase, others	4
**GH35**	PF01301	Glycosyl hydrolase family 35	β-galactosidase, exo-β-glucosaminidase	20
**GH38**	PF01074	Glycosyl hydrolase family 38	α-mannosidase, N-mannosyl-oligosaccharide α-1,3-1,6-mannosidase	21

Supplementary Table 4 Summary of Glycosyl Hydrolase (GH) in red palm weevil

Number of introns	GH16 (Female)	Female (log2fold)	Male (log2fold)	Egg (log2fold)	Larvae (log2fold)	Pupae (log2fold)
**4**	FM_020572-T1	6.541753075	5.943302219	2	4.774280302	3.341238925
**3**	FM_019802-T1	6.158783316	6.15126173	2	2	2
**2**	FM_019803-T1	8.31941106	8.861576217	4.875888275	6.111339845	8.249698663
**7**	FM_007011-T1	8.158273273	8.13915537	3.589783061	2	3.887134951
**0**	FM_000628-T1	4.191468923	2.767752768	2	2	2
**0**	FM_020068-T1	4.989497925	2.900925091	2	2	2
**0**	FM_021131-T1	4.785029822	2.593975215	2	2	2
**Introns**	**Average_Log2fold**	7.294555181	7.273823884	3.116417834	3.721405037	4.369518135
**No introns**	**Average_Log2fold**	4.655332223	2.754217691	2	2	2

Supplementary Table 5 Log2fold change in Expression difference of different GH16 with 0 intron to 7 introns across different developmental stages.

Supplementary methods

Genome assembly and annotation

We adopted a de novo assembly strategy that combined Illumina short insert libraries, linked reads (10X Genomics), and Oxford nanopore sequence. We started by de novo assembling 10X paired-end Illumina (150bp) sequences using ABYSS [1] and independently generated a second 'megabubbles' assembly with Supernova using linked reads (10X Genomics). The first run of linked reads produced 166 million reads with a N50 length of 37.9 Kbp, giving coverage of 32.38 X for the *de novo* assembly. After sequencing more libraries for higher coverage and a better assembly, the assembly size was only 292.84 Mbp, which is less than the expected size of the genome. The second run produced 387.78 million reads with a N50 length of 146.32 Kbp, giving coverage of 76.75 X on the *de novo* assembly. We used Supernova [2] for 10x Genomics linked reads. The resultant 'megabubbles' assembly from this run was used to scaffold the male and female ABYSS [1] assemblies from 2x150bp Illumina paired end data. Oxford Nanopore long reads were generated for the *R. ferrugineus* male. We constructed an assembly from Nanopore reads using wtdbg v1.2.8 [3] (https://github.com/fantasticair/wtdbg-1.2.8) and this was followed by two round of polishing with Pilon [4] version 1.21 (bwa Illumina reads) and Racon version 1.2.0 [5] (minimap2 aligning Nanopore long reads). A hybrid assembly was generated using DBG2OLC assembler combining ABYSS Illumina contigs and long reads Nanopore [6]. Finally we used QuickMerge version 0.2 [7] to merge the different assemblies and generate a final merged assembly setting "-hco 5.0 -c 1.5 -l 300,000 -ml 5,000". We evaluated the different assemblies using Quast [8]. Using the long read Oxford Nanopore assembly; we completely assembled and annotated the mitochondrial genome.

Scaffolds shorter than 5 kbp were removed from the genome, and the genome was syntenically aligned against the red flour beetle (*Tribolium castaneum*) reference genome (version 5.2, GeneBank Assembly accession GCA_000002335.3) using Chromosemble in Satsuma v3.1.0 [9] to generate pseudochromosome-level assemblies for male and female.

Funannotate Gene prediction was carried out by both *de novo* (GeneMark [10] and Augustus [11] and evidence-based methods (EVM [12]). For Augustus *de novo* gene prediction, we used "rhodnius" which

is the closest model to our beetle that is available in their database. Non-coding tRNA genes were predicted using tRNAscan-SE [13]. Gene prediction accuracy was confirmed by searching against the insect BUSCO [14] database. Predicted proteins were similarity searched against NCBI and UniProt Insecta' protein database by BlastP [15] with the e-value e-10. Protein domain analysis was carried out by InterProscan [16]. Protein family classification was carried out using Pfam [17] by hmmer [18] tool. Gene Ontology information associated with the proteins was extracted from the InterPro and UniProt database. Pathway enzyme mapping was carried out using KEGG-KAAS tool [19], and all available insect KEGG models were used for pathway prediction. Enrichment analysis (http://supfam.org/SUPERFAMILY/cgi-bin/dcenrichment.cgi) was done using PFAM domains.

Structural variation

The generated Illumina reads Hiseq 2500 (2x 150bp) were trimmed using Trimmomatic [20]. Trimmed reads were aligned separately to the male and female genome assembly using BWA [21] version 1.0 samtools [22] version 1.2. Duplicates were marked and removed using Picard tools version 1.52 (http://sourceforge.net/projects/picard/files/picard-tools/). Coverage depth for alignment files bams was computed using samtools. Normalized Read-depth variation analysis was performed using CNVnator [23] (version 0.2.7). Aligned bams were used as input for CNVnator to extract read alignment information. A bin size of 1 Kb was used in the intermediate processing of the bams as well as when calling variants. A table of duplication and deletion is generated. We discarded any duplication/deletion more than > 100 Kb as well as hits that span gaps and beginning of a scaffold. Tandem duplication was screened using the software SoftV [24].

Horizontally gene transfer

Briefly, the approach uses a combination of homology and phylogenetic sequence comparison. We applied that for *R. ferrugineus*. We used Diamond 'BlastP' (e value ≤ 10-5) to compare our proteomes to the UniRef90 databases [25]. To eliminate any hits to our species of interest, we omitted their Taxonomic ID(s) from further analysis (e.g. 354439 of *R. ferrugineus*). We applied

two metrics the HGT index [26] h_U and the Consensus Hit Support (CHS) [27] to select putative candidates. For putative HGT_C candidate, a $h_U \geq 30$ and $CHS_{OUT} \geq 90\%$ was applied. We discarded any candidates that have occupied ≥ 90% of scaffolds, as those considered as contaminants. For HGT_C, we tested for physical linkage and looked for presence of intron in the HGT_C. Finally, phylogenetic tree was generated using IQ-TREE v.1.5.3 [28] for all candidates with automatic model selection using 1000 bootstrap replicates.

Supplementary references

1. Simpson JT, Wong K, Jackman SD, Schein JE, Jones SJ, Birol I. ABySS: a parallel assembler for short read sequence data. *Genome research* **19**, 1117-1123 (2009).

2. Weisenfeld NI, Kumar V, Shah P, Church DM, Jaffe DB. Direct determination of diploid genome sequences. *Genome research* **27**, 757-767 (2017).

3. Ruan J, Li H. Fast and accurate long-read assembly with wtdbg2. *Nature Methods* **17**, 155-158 (2020).

4. Walker BJ*, et al.* Pilon: an integrated tool for comprehensive microbial variant detection and genome assembly improvement. *PloS one* **9**, (2014).

5. Vaser R, Sović I, Nagarajan N, Šikić M. Fast and accurate de novo genome assembly from long uncorrected reads. *Genome research* **27**, 737-746 (2017).

6. Ye C, Hill CM, Wu S, Ruan J, Ma ZS. DBG2OLC: efficient assembly of large genomes using long erroneous reads of the third generation sequencing technologies. *Scientific reports* **6**, 31900 (2016).

7. Chakraborty M, Baldwin-Brown JG, Long AD, Emerson J. Contiguous and accurate de novo assembly of metazoan genomes with modest long read coverage. *Nucleic acids research* **44**, e147-e147 (2016).

8. Gurevich A, Saveliev V, Vyahhi N, Tesler G. QUAST: quality assessment tool for genome assemblies. *Bioinformatics* **29**, 1072-1075 (2013).

9. Grabherr MG*, et al.* Genome-wide synteny through highly sensitive sequence alignment: Satsuma. *Bioinformatics* **26**, 1145-1151 (2010).

10. Besemer J, Borodovsky M. GeneMark: web software for gene finding in prokaryotes, eukaryotes and viruses. *Nucleic acids research* **33**, W451-W454 (2005).

11. Stanke M, Keller O, Gunduz I, Hayes A, Waack S, Morgenstern B. AUGUSTUS: ab initio prediction of alternative transcripts. *Nucleic acids research* **34**, W435-W439 (2006).

12. Haas BJ*, et al.* Automated eukaryotic gene structure annotation using EVidenceModeler and the Program to Assemble Spliced Alignments. *Genome biology* **9**, R7 (2008).

13. Lowe TM, Eddy SR. tRNAscan-SE: a program for improved detection of transfer RNA genes in genomic sequence. *Nucleic acids research* **25**, 955-964 (1997).

14. Simão FA, Waterhouse RM, Ioannidis P, Kriventseva EV, Zdobnov EM. BUSCO: assessing genome assembly and annotation completeness with single-copy orthologs. *Bioinformatics* **31**, 3210-3212 (2015).

15. Altschul SF, Gish W, Miller W, Myers EW, Lipman DJ. Basic local alignment search tool. *Journal of molecular biology* **215**, 403-410 (1990).

16. Zdobnov EM, Apweiler R. InterProScan–an integration platform for the signature-recognition methods in InterPro. *Bioinformatics* **17**, 847-848 (2001).

17. Bateman A*, et al.* The Pfam protein families database. *Nucleic acids research* **30**, 276-280 (2002).

18. Finn RD, Clements J, Eddy SR. HMMER web server: interactive sequence similarity searching. *Nucleic acids research* **39**, W29-W37 (2011).

19. Moriya Y, Itoh M, Okuda S, Yoshizawa AC, Kanehisa M. KAAS: an automatic genome annotation and pathway reconstruction server. *Nucleic acids research* **35**, W182-W185 (2007).

20. Bolger AM, Lohse M, Usadel B. Trimmomatic: a flexible trimmer for Illumina sequence data. *Bioinformatics* **30**, 2114-2120 (2014).

21. Li H, Durbin R. Fast and accurate short read alignment with Burrows–Wheeler transform. *bioinformatics* **25**, 1754-1760 (2009).

22. Li H*, et al.* The sequence alignment/map format and SAMtools. *Bioinformatics* **25**, 2078-2079 (2009).

23. Abyzov A, Urban AE, Snyder M, Gerstein M. CNVnator: an approach to discover, genotype, and characterize typical and atypical CNVs from family and population genome sequencing. *Genome research* **21**, 974-984 (2011).

24. Bartenhagen C, Dugas M. Robust and exact structural variation detection with paired-end and soft-clipped alignments: SoftSV compared with eight algorithms. *Briefings in bioinformatics* **17**, 51-62 (2016).

25. Suzek BE, Huang H, McGarvey P, Mazumder R, Wu CH. UniRef: comprehensive and non-redundant UniProt reference clusters. *Bioinformatics* **23**, 1282-1288 (2007).

26. Boschetti C*, et al.* Biochemical diversification through foreign gene expression in bdelloid rotifers. *PLoS genetics* **8**, (2012).

27. Koutsovoulos G*, et al.* No evidence for extensive horizontal gene transfer in the genome of the tardigrade Hypsibius dujardini. *Proceedings of the National Academy of Sciences* **113**, 5053-5058 (2016).

28. Nguyen L-T, Schmidt HA, Von Haeseler A, Minh BQ. IQ-TREE: a fast and effective stochastic algorithm for estimating maximum-likelihood phylogenies. *Molecular biology and evolution* **32**, 268-274 (2015).
