# Supplementary material for: The genome of pest Rhynchophorus ferrugineus reveals gene families important at the plant-beetle interface
Source: Commun Biol. 2020 Jun 24;3:323. doi: 10.1038/s42003-020-1060-8 (PMC7314810; doi:10.1038/s42003-020-1060-8)
Supplement: Supplementary file 17 — Supplementary data file 17 [file 42003_2020_1060_MOESM17_ESM.docx]

Parameter estimates and likelihood scores for transient receptor potential ion channels (Melastatin) (TRPM) genes under models of variable ω ratios.

| Nested model pairs | d _N_/d_S_ ^b^ | Parameter estimates ^c^ | PSS (*: P>95%; **: P>99%) ^d^ | Likelihood |
| --- | --- | --- | --- | --- |
| M0:one-ratio (1)^a^ | 0.0375 | ω= 0.0375 |  | -10637.580 |
| M3: discrete (5) | 1.4731 | p_0_= 0.658,p_1_= 0.307, (p_2_= 0.015)  ω_0_= 0.00, ω_1_= 0.069, ω_2_=1.511 | 2 K 1.000**  4 S 1.000**  7 H 0.997**  10 T 0.999**  1628 V 1.000**  1629 Y 1.000**  1630 S 1.000**  1631 K 1.000**  1632 N 1.000**  1633 E 1.000**  1635 T 1.000**  1690 I 1.000** | -10467.920 |
| M1: neutral (1) | 0.0758 | p_0_= 0.940,p_1_= 0.059  ω_0_= 0.017, ω_1_= 1 |  | -10550.276 |
| M2: selection (3) | 1.473 | p_0_= 0.658,p_1_= 0.307, (p_2_= 0.015)  ω_0_= 0.00, (ω_1_= 0.069), ω_2_= 1.511 | 2 K 1.000**  4 S 1.000**  7 H 0.997**  10 T 0.999**  1628 V 1.000**  1629 Y 1.000**  1630 S 1.000**  1631 K 1.000**  1632 N 1.000**  1633 E 1.000**  1635 T 1.000**  1690 I 1.000** | -10467.920 |
| M7: beta (2)  M8: beta + ω>1(4)  M8a: beta + ω=1(4) | 0.0529  1.4091  0.0684 | p= 0.030, q= 0.280  p_0_= 0.981,(p_1_= 0.018)  p= 0.072, q=1.265, ω=75.151  p_0_= 0.956,(p_1_= 0.043)  p= 0.029, q= 0.348, ω=1 | 2 K 1.000**  4 S 1.000**  7 H 0.997**  10 T 0.999**  1628 V 1.000**  1629 Y 1.000**  1630 S 1.000**  1631 K 1.000**  1632 N 1.000**  1633 E 1.000**  1635 T 1.000**  1690 I 1.000** | -10564.468  -10468.598  -10550.579 |

| Gene | Model ^e^ | P value |
| --- | --- | --- |
| TRPM | M3 vs M0 | 3.02478603897e-70 |
| TRPM | M2 vs M1 | 4.77568943492e-36 |
| TRPM | M8 vs M7 | 2.3125733429e-42 |
| TRPM | M8 vs M8a | 1.54055657418e-37 |

^a^ The number of free parameters in the ω distribution.

^b^ Average ratio d_N_/d_S_ of all sites for the GH16 gene alignment.

^c^ The number in parentheses are not free parameters.

^d^ Number of positively selected sites.

^e^ Likelihood ratio test statistics for models of variable selective pressure among codons.
