# Supplementary figures and images for: The genome of pest Rhynchophorus ferrugineus reveals gene families important at the plant-beetle interface (part 2 of 2)
Source: Commun Biol. 2020 Jun 24;3:323. doi: 10.1038/s42003-020-1060-8 (PMC7314810; doi:10.1038/s42003-020-1060-8)

# Evolution of the gene family "672" ( $p=0.017$ )

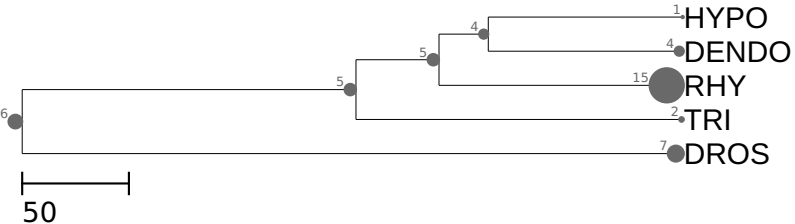

Supplement: Supplementary file 18 — Supplementary data file 13 [file 42003_2020_1060_MOESM18_ESM.zip › Additional_file_12/672.pdf]

# Evolution of the gene family "100" ( $p=0.012$ )

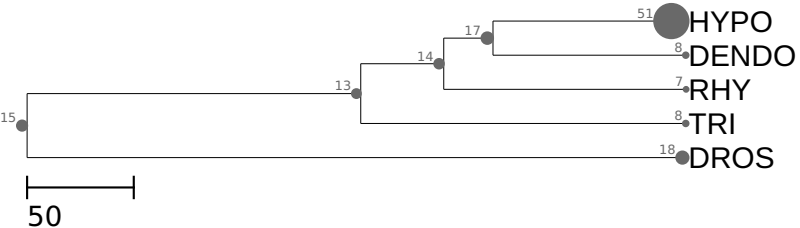

Supplement: Supplementary file 18 — Supplementary data file 13 [file 42003_2020_1060_MOESM18_ESM.zip › Additional_file_12/100.pdf]

Evolution of the gene family "128" (p=0.012)

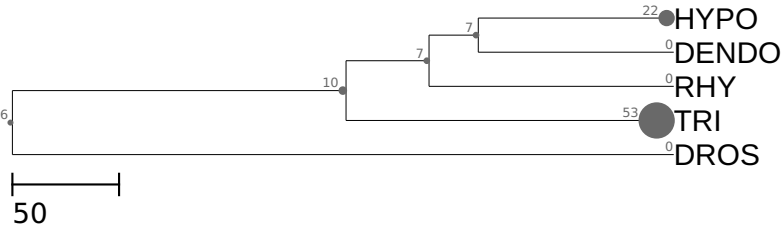

Supplement: Supplementary file 18 — Supplementary data file 13 [file 42003_2020_1060_MOESM18_ESM.zip › Additional_file_12/128.pdf]

Evolution of the gene family "1331" (p=0.012)

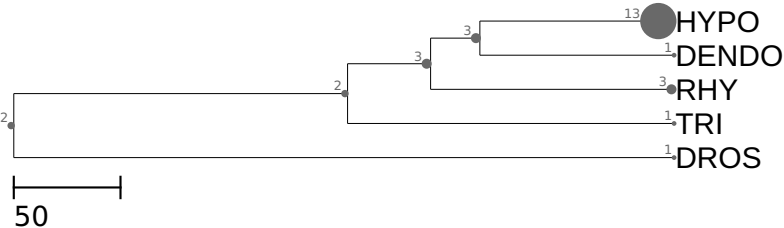

Supplement: Supplementary file 18 — Supplementary data file 13 [file 42003_2020_1060_MOESM18_ESM.zip › Additional_file_12/1331.pdf]

Evolution of the gene family "316" (p=0.0)

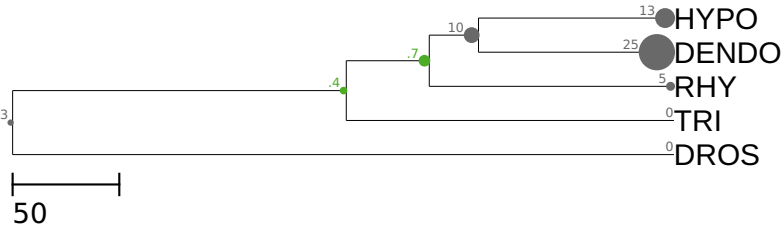

Supplement: Supplementary file 18 — Supplementary data file 13 [file 42003_2020_1060_MOESM18_ESM.zip › Additional_file_12/316.pdf]

# Evolution of the gene family "302" ( $p=0.008$ )

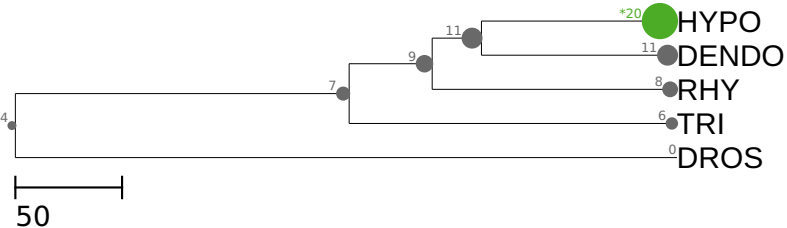

Supplement: Supplementary file 18 — Supplementary data file 13 [file 42003_2020_1060_MOESM18_ESM.zip › Additional_file_12/302.pdf]

# Evolution of the gene family "1127" ( $p=0.01$ )

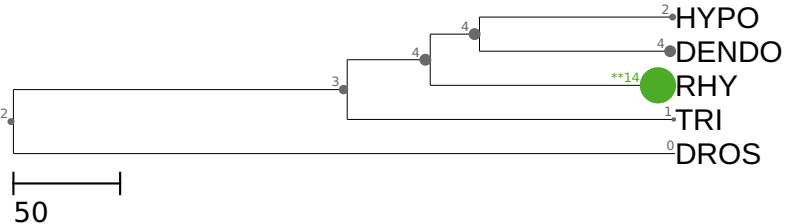

Supplement: Supplementary file 18 — Supplementary data file 13 [file 42003_2020_1060_MOESM18_ESM.zip › Additional_file_12/1127.pdf]

# Evolution of the gene family "465" ( $p=0.043$ )

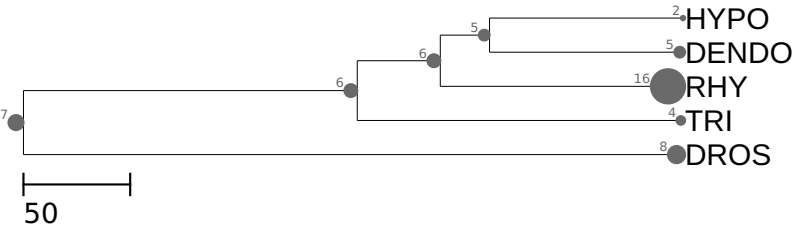

Supplement: Supplementary file 18 — Supplementary data file 13 [file 42003_2020_1060_MOESM18_ESM.zip › Additional_file_12/465.pdf]

Evolution of the gene family "1456" (p=0.006)

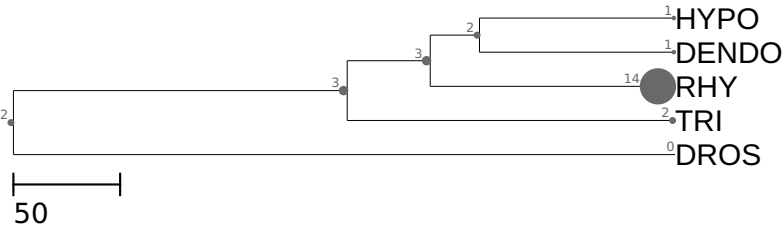

Supplement: Supplementary file 18 — Supplementary data file 13 [file 42003_2020_1060_MOESM18_ESM.zip › Additional_file_12/1456.pdf]

# Evolution of the gene family "883" ( $p=0.04$ )

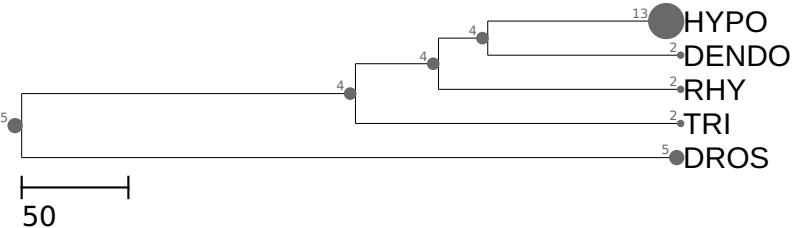

Supplement: Supplementary file 18 — Supplementary data file 13 [file 42003_2020_1060_MOESM18_ESM.zip › Additional_file_12/883.pdf]

# Evolution of the gene family "129" ( $p=0.001$ )

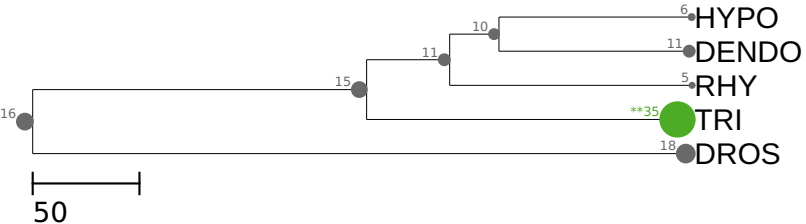

Supplement: Supplementary file 18 — Supplementary data file 13 [file 42003_2020_1060_MOESM18_ESM.zip › Additional_file_12/129.pdf]

# Evolution of the gene family "673" ( $p=0.005$ )

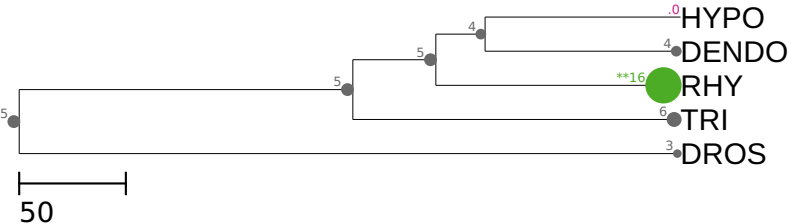

Supplement: Supplementary file 18 — Supplementary data file 13 [file 42003_2020_1060_MOESM18_ESM.zip › Additional_file_12/673.pdf]

Evolution of the gene family "908" (p=0.0)

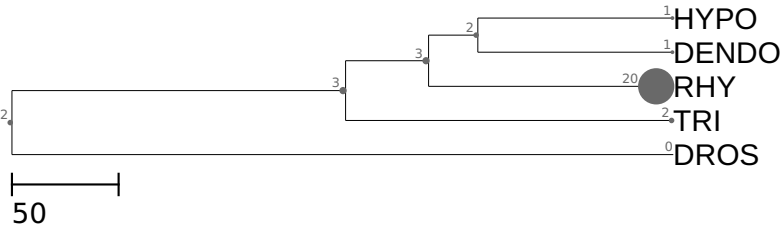

Supplement: Supplementary file 18 — Supplementary data file 13 [file 42003_2020_1060_MOESM18_ESM.zip › Additional_file_12/908.pdf]

# Evolution of the gene family "713" ( $p=0.007$ )

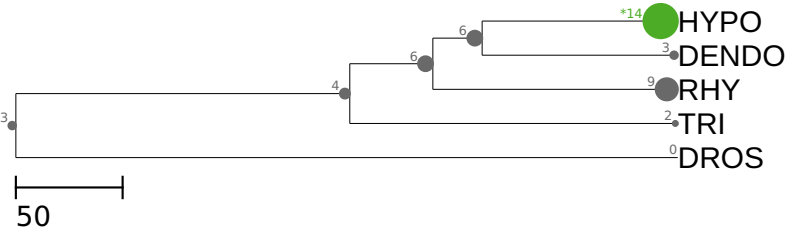

Supplement: Supplementary file 18 — Supplementary data file 13 [file 42003_2020_1060_MOESM18_ESM.zip › Additional_file_12/713.pdf]

Evolution of the gene family "1046" (p=0.003)

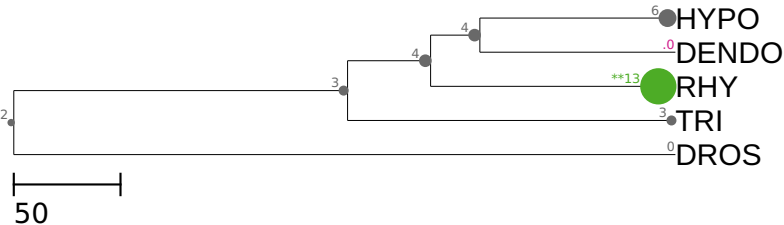

Supplement: Supplementary file 18 — Supplementary data file 13 [file 42003_2020_1060_MOESM18_ESM.zip › Additional_file_12/1046.pdf]

# Evolution of the gene family "511" (p=0.0)

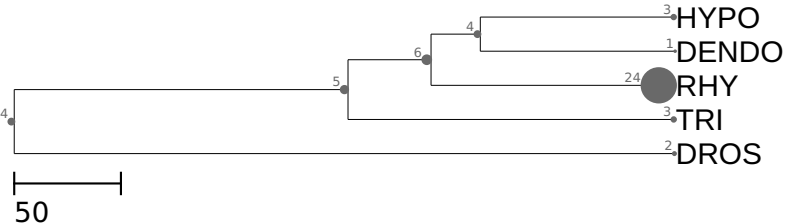

Supplement: Supplementary file 18 — Supplementary data file 13 [file 42003_2020_1060_MOESM18_ESM.zip › Additional_file_12/511.pdf]

# Evolution of the gene family "501" ( $p=0.001$ )

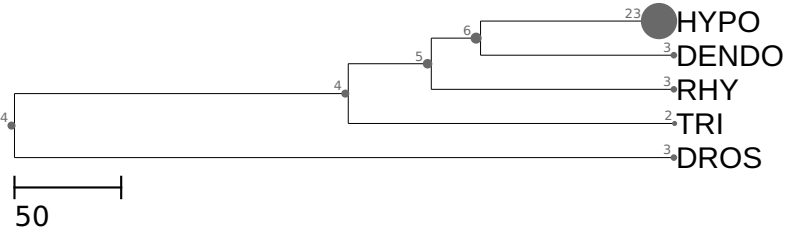

Supplement: Supplementary file 18 — Supplementary data file 13 [file 42003_2020_1060_MOESM18_ESM.zip › Additional_file_12/501.pdf]

# Evolution of the gene family "267" ( $p=0.04$ )

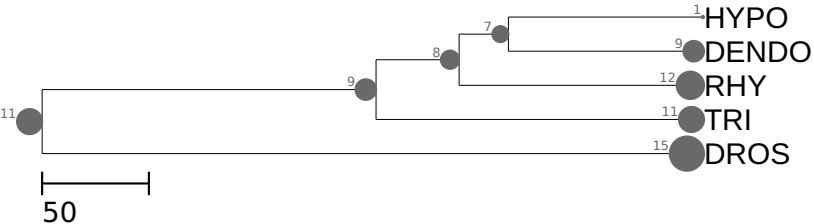

Supplement: Supplementary file 18 — Supplementary data file 13 [file 42003_2020_1060_MOESM18_ESM.zip › Additional_file_12/267.pdf]

# Evolution of the gene family "273" ( $p=0.003$ )

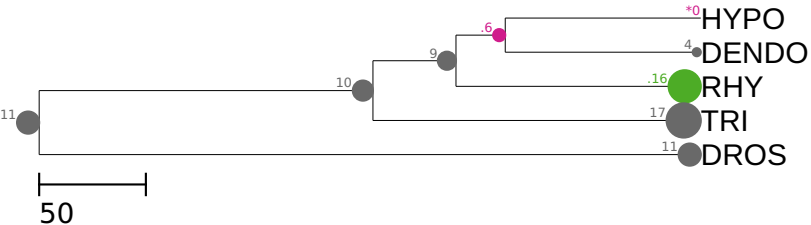

Supplement: Supplementary file 18 — Supplementary data file 13 [file 42003_2020_1060_MOESM18_ESM.zip › Additional_file_12/273.pdf]

Evolution of the gene family "298" (p=0.001)

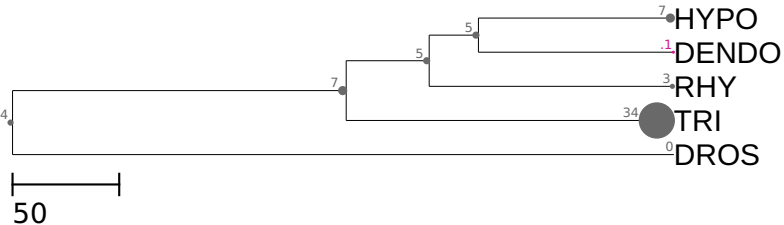

Supplement: Supplementary file 18 — Supplementary data file 13 [file 42003_2020_1060_MOESM18_ESM.zip › Additional_file_12/298.pdf]

Evolution of the gene family "1240" (p=0.002)

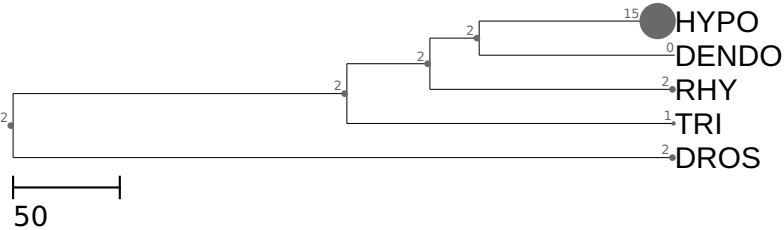

Supplement: Supplementary file 18 — Supplementary data file 13 [file 42003_2020_1060_MOESM18_ESM.zip › Additional_file_12/1240.pdf]

# Evolution of the gene family "893" ( $p=0.006$ )

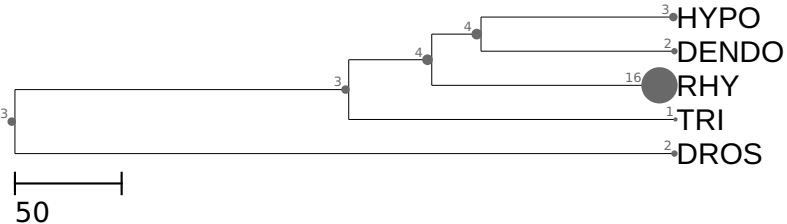

Supplement: Supplementary file 18 — Supplementary data file 13 [file 42003_2020_1060_MOESM18_ESM.zip › Additional_file_12/893.pdf]

# Evolution of the gene family "139" ( $p=0.01$ )

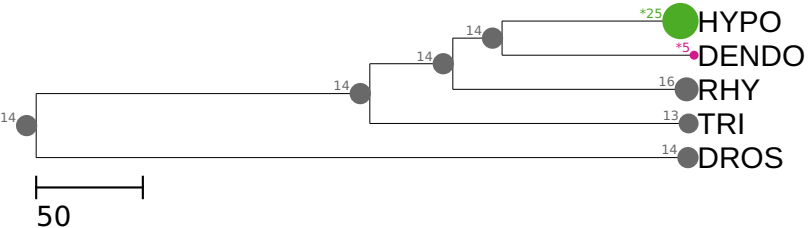

Supplement: Supplementary file 18 — Supplementary data file 13 [file 42003_2020_1060_MOESM18_ESM.zip › Additional_file_12/139.pdf]

# Evolution of the gene family "1308" (p=0.012)

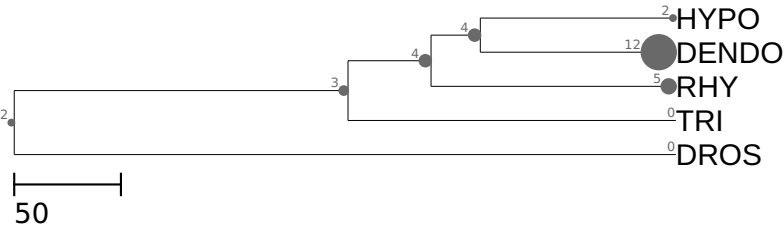

Supplement: Supplementary file 18 — Supplementary data file 13 [file 42003_2020_1060_MOESM18_ESM.zip › Additional_file_12/1308.pdf]

Evolution of the gene family "1687" (p=0.0)

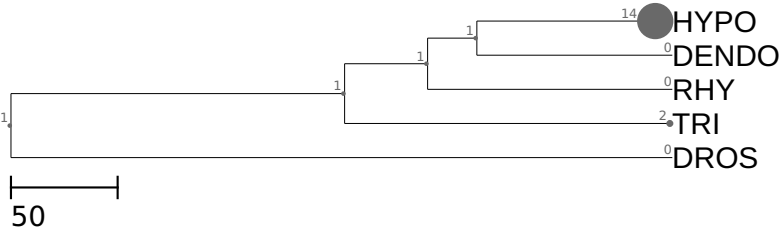

Supplement: Supplementary file 18 — Supplementary data file 13 [file 42003_2020_1060_MOESM18_ESM.zip › Additional_file_12/1687.pdf]

# Evolution of the gene family "313" ( $p=0.018$ )

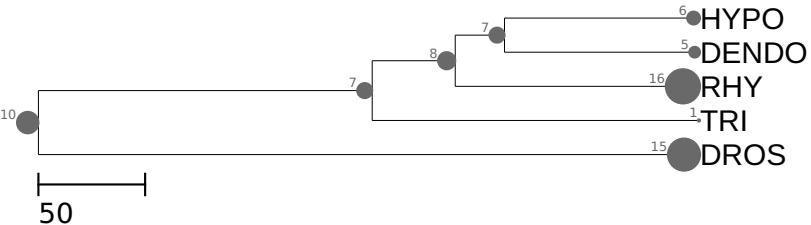

Supplement: Supplementary file 18 — Supplementary data file 13 [file 42003_2020_1060_MOESM18_ESM.zip › Additional_file_12/313.pdf]

Evolution of the gene family "461" (p=0.001)

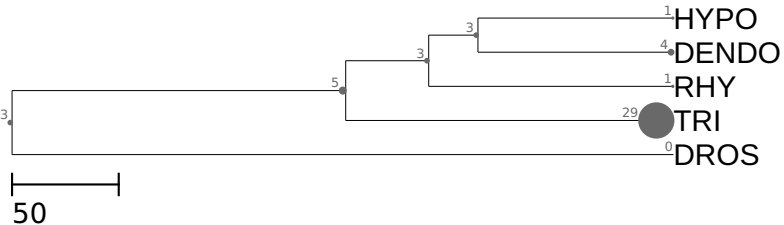

Supplement: Supplementary file 18 — Supplementary data file 13 [file 42003_2020_1060_MOESM18_ESM.zip › Additional_file_12/461.pdf]

# Evolution of the gene family "306" ( $p=0.002$ )

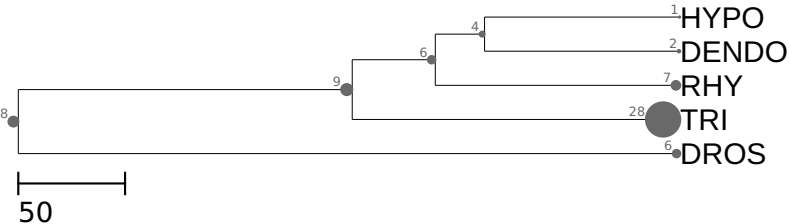

Supplement: Supplementary file 18 — Supplementary data file 13 [file 42003_2020_1060_MOESM18_ESM.zip › Additional_file_12/306.pdf]

Evolution of the gene family "879" (p=0.0)

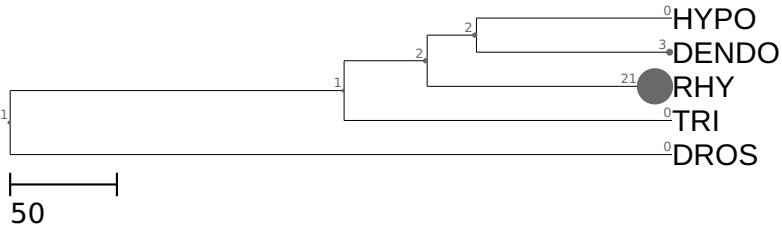

Supplement: Supplementary file 18 — Supplementary data file 13 [file 42003_2020_1060_MOESM18_ESM.zip › Additional_file_12/879.pdf]

# Evolution of the gene family "845" ( $p=0.024$ )

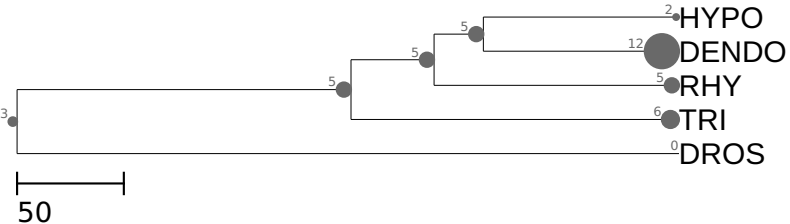

Supplement: Supplementary file 18 — Supplementary data file 13 [file 42003_2020_1060_MOESM18_ESM.zip › Additional_file_12/845.pdf]

# Evolution of the gene family "110" ( $p=0.037$ )

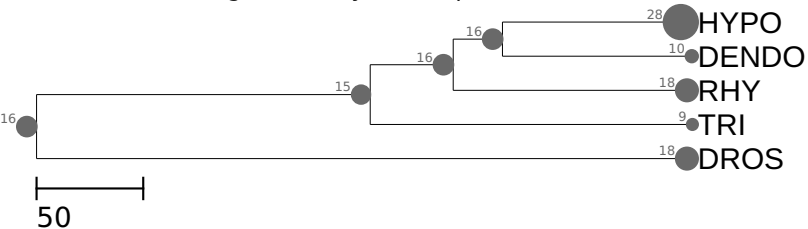

Supplement: Supplementary file 18 — Supplementary data file 13 [file 42003_2020_1060_MOESM18_ESM.zip › Additional_file_12/110.pdf]

# Evolution of the gene family "138" ( $p=0.004$ )

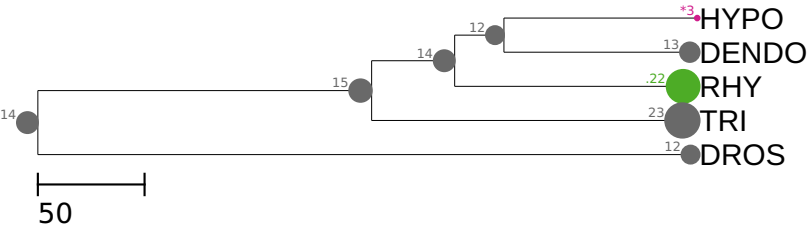

Supplement: Supplementary file 18 — Supplementary data file 13 [file 42003_2020_1060_MOESM18_ESM.zip › Additional_file_12/138.pdf]

Evolution of the gene family "919" (p=0.0)

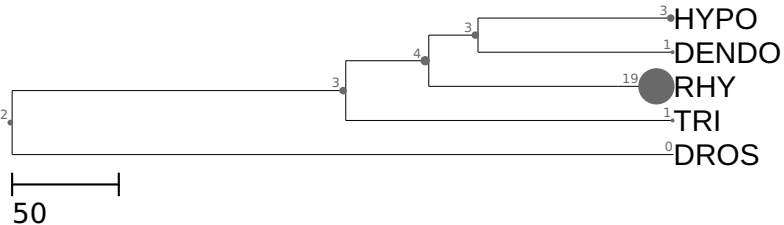

Supplement: Supplementary file 18 — Supplementary data file 13 [file 42003_2020_1060_MOESM18_ESM.zip › Additional_file_12/919.pdf]

Evolution of the gene family "2210" (p=0.017)

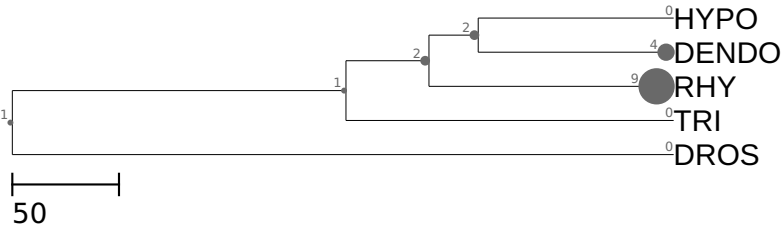

Supplement: Supplementary file 18 — Supplementary data file 13 [file 42003_2020_1060_MOESM18_ESM.zip › Additional_file_12/2210.pdf]

# Evolution of the gene family "258" ( $p=0.025$ )

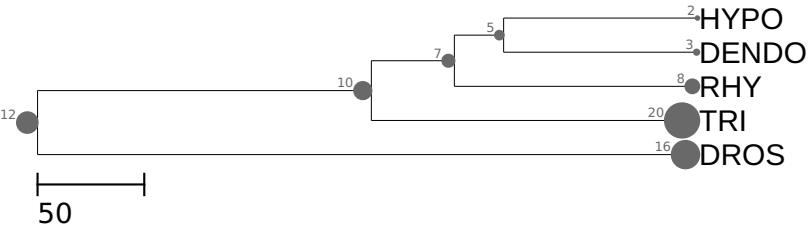

Supplement: Supplementary file 18 — Supplementary data file 13 [file 42003_2020_1060_MOESM18_ESM.zip › Additional_file_12/258.pdf]

# Evolution of the gene family "264" ( $p=0.001$ )

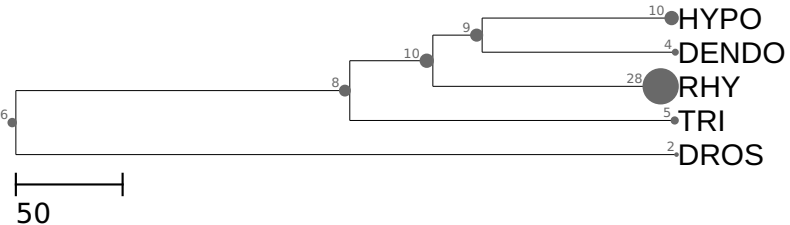

Supplement: Supplementary file 18 — Supplementary data file 13 [file 42003_2020_1060_MOESM18_ESM.zip › Additional_file_12/264.pdf]

# Evolution of the gene family "714" ( $p=0.015$ )

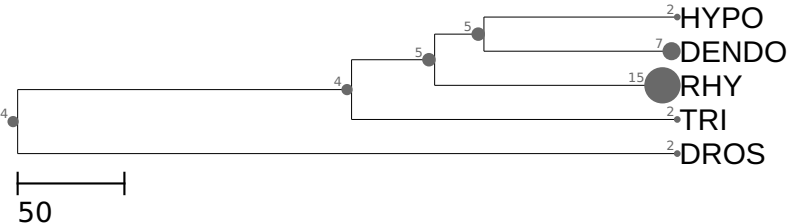

Supplement: Supplementary file 18 — Supplementary data file 13 [file 42003_2020_1060_MOESM18_ESM.zip › Additional_file_12/714.pdf]

# Evolution of the gene family "884" ( $p=0.007$ )

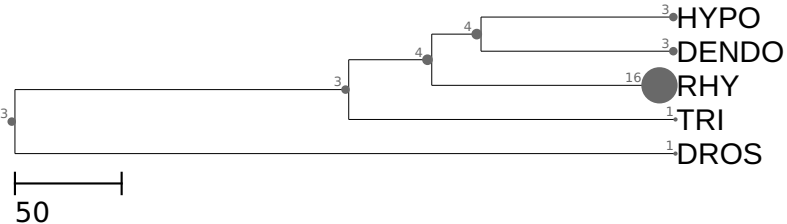

Supplement: Supplementary file 18 — Supplementary data file 13 [file 42003_2020_1060_MOESM18_ESM.zip › Additional_file_12/884.pdf]

# Evolution of the gene family "890" ( $p=0.046$ )

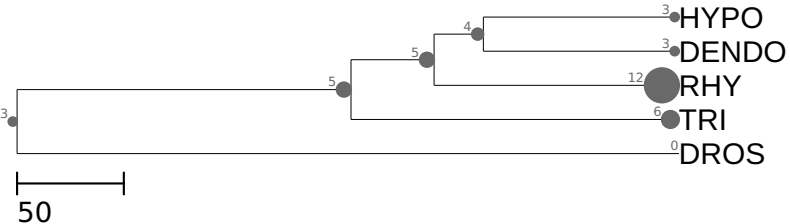

Supplement: Supplementary file 18 — Supplementary data file 13 [file 42003_2020_1060_MOESM18_ESM.zip › Additional_file_12/890.pdf]

# Evolution of the gene family "648" ( $p=0.029$ )

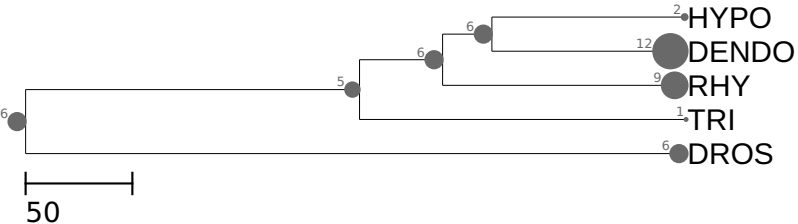

Supplement: Supplementary file 18 — Supplementary data file 13 [file 42003_2020_1060_MOESM18_ESM.zip › Additional_file_12/648.pdf]

# Evolution of the gene family "106" ( $p=0.009$ )

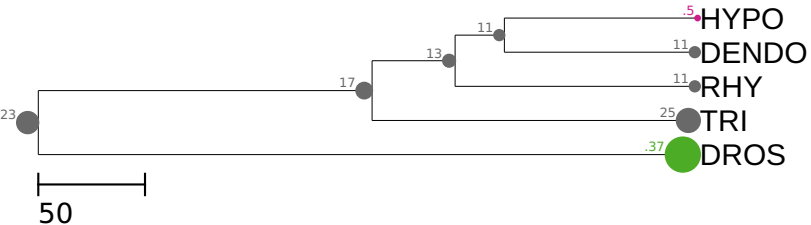

Supplement: Supplementary file 18 — Supplementary data file 13 [file 42003_2020_1060_MOESM18_ESM.zip › Additional_file_12/106.pdf]

# Evolution of the gene family "853" ( $p=0.046$ )

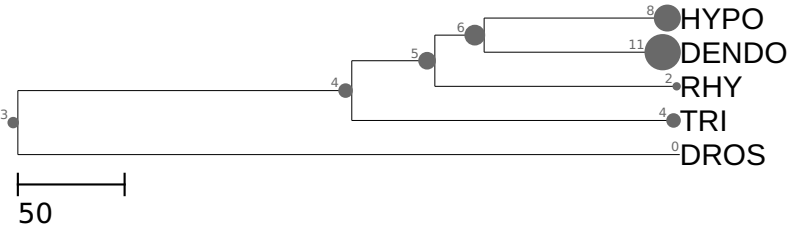

Supplement: Supplementary file 18 — Supplementary data file 13 [file 42003_2020_1060_MOESM18_ESM.zip › Additional_file_12/853.pdf]

Evolution of the gene family "489" (p=0.001)

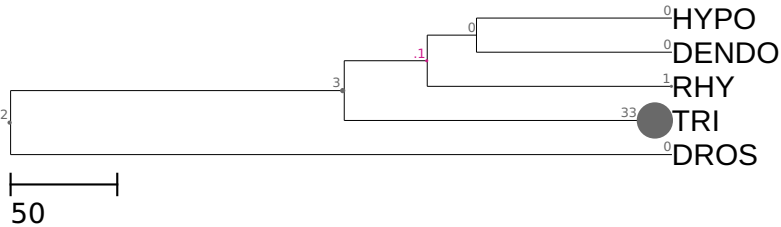

Supplement: Supplementary file 18 — Supplementary data file 13 [file 42003_2020_1060_MOESM18_ESM.zip › Additional_file_12/489.pdf]

Evolution of the gene family "3044" (p=0.039)

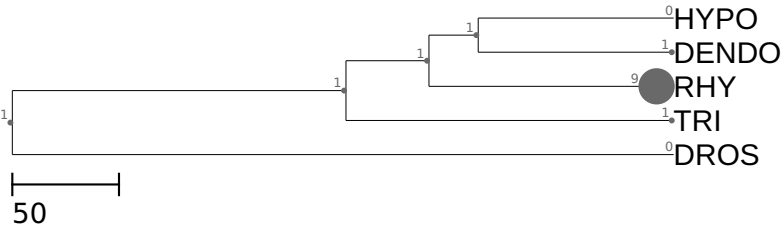

Supplement: Supplementary file 18 — Supplementary data file 13 [file 42003_2020_1060_MOESM18_ESM.zip › Additional_file_12/3044.pdf]

Evolution of the gene family "3092" (p=0.031)

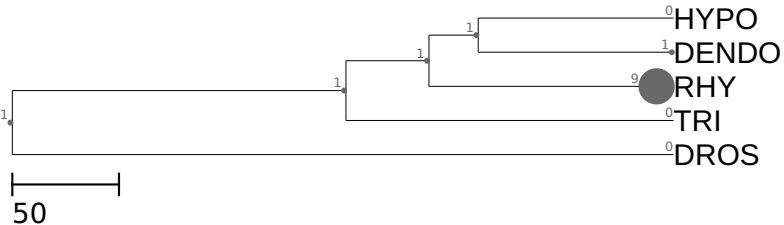

Supplement: Supplementary file 18 — Supplementary data file 13 [file 42003_2020_1060_MOESM18_ESM.zip › Additional_file_12/3092.pdf]

# Evolution of the gene family "305" ( $p=0.016$ )

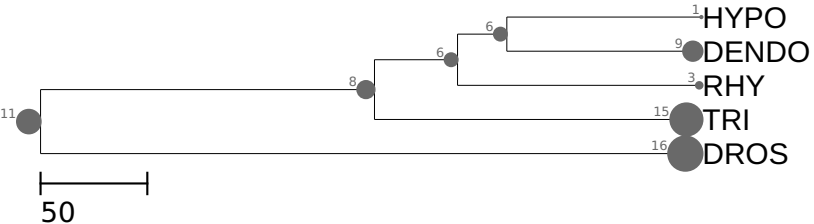

Supplement: Supplementary file 18 — Supplementary data file 13 [file 42003_2020_1060_MOESM18_ESM.zip › Additional_file_12/305.pdf]

# Evolution of the gene family "852" ( $p=0.046$ )

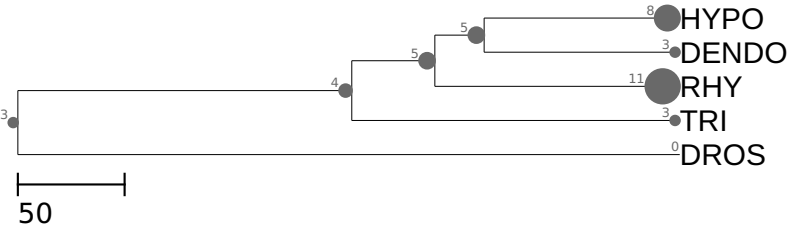

Supplement: Supplementary file 18 — Supplementary data file 13 [file 42003_2020_1060_MOESM18_ESM.zip › Additional_file_12/852.pdf]

Evolution of the gene family "932" (p=0.0)

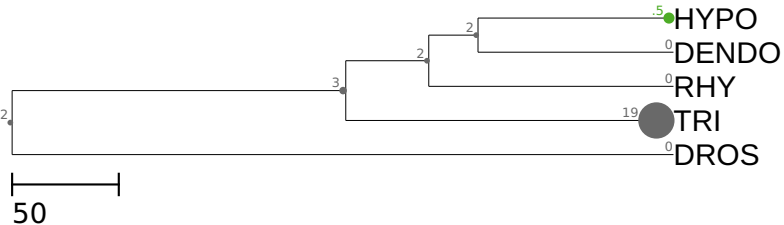

Supplement: Supplementary file 18 — Supplementary data file 13 [file 42003_2020_1060_MOESM18_ESM.zip › Additional_file_12/932.pdf]

# Evolution of the gene family "265" ( $p=0.01$ )

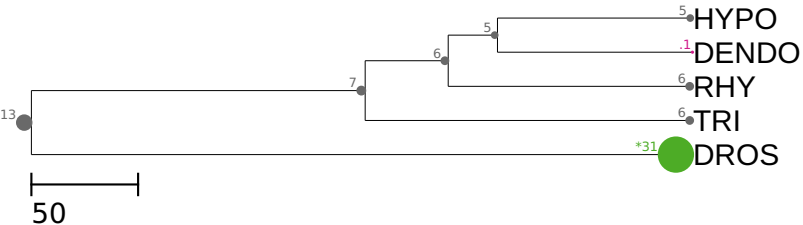

Supplement: Supplementary file 18 — Supplementary data file 13 [file 42003_2020_1060_MOESM18_ESM.zip › Additional_file_12/265.pdf]

# Evolution of the gene family "1918" ( $p=0.024$ )

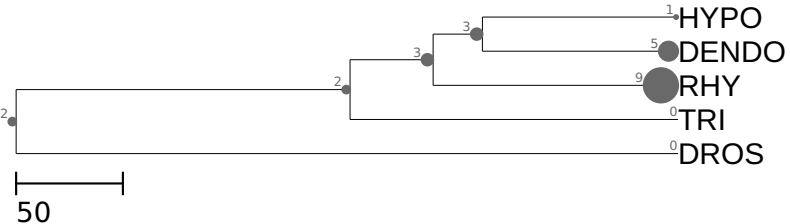

Supplement: Supplementary file 18 — Supplementary data file 13 [file 42003_2020_1060_MOESM18_ESM.zip › Additional_file_12/1918.pdf]

# Evolution of the gene family "532" ( $p=0.021$ )

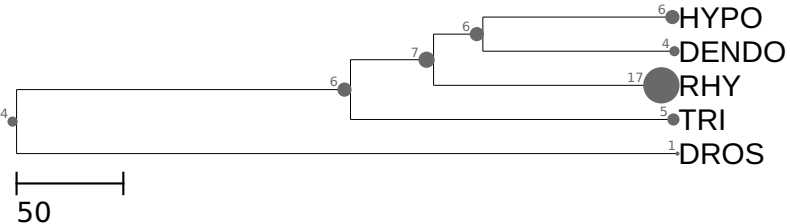

Supplement: Supplementary file 18 — Supplementary data file 13 [file 42003_2020_1060_MOESM18_ESM.zip › Additional_file_12/532.pdf]

# Evolution of the gene family "240" ( $p=0.009$ )

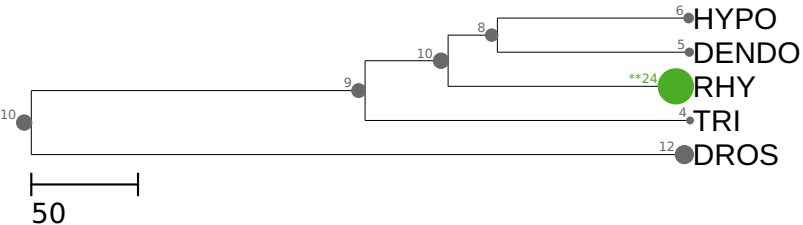

Supplement: Supplementary file 18 — Supplementary data file 13 [file 42003_2020_1060_MOESM18_ESM.zip › Additional_file_12/240.pdf]

# Evolution of the gene family "268" ( $p=0.048$ )

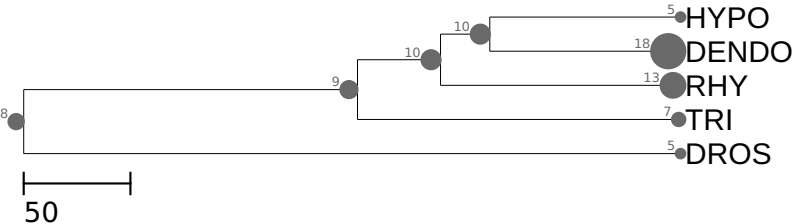

Supplement: Supplementary file 18 — Supplementary data file 13 [file 42003_2020_1060_MOESM18_ESM.zip › Additional_file_12/268.pdf]

# Evolution of the gene family "283" ( $p=0.026$ )

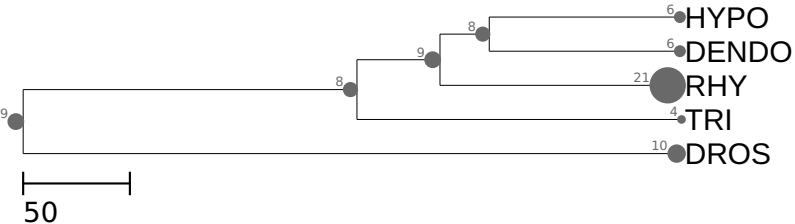

Supplement: Supplementary file 18 — Supplementary data file 13 [file 42003_2020_1060_MOESM18_ESM.zip › Additional_file_12/283.pdf]

Evolution of the gene family "1717" (p=0.002)

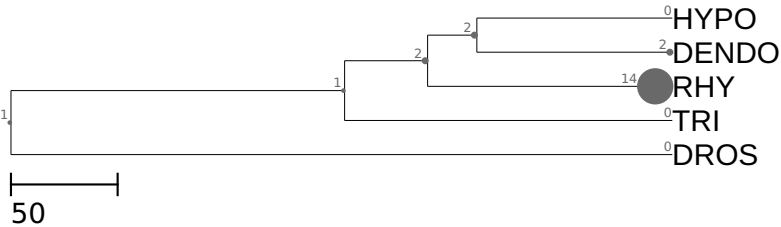

Supplement: Supplementary file 18 — Supplementary data file 13 [file 42003_2020_1060_MOESM18_ESM.zip › Additional_file_12/1717.pdf]

# Evolution of the gene family "1059" ( $p=0.03$ )

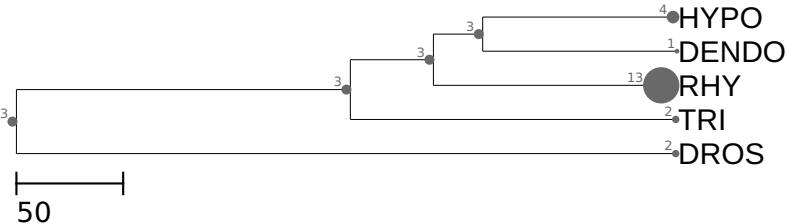

Supplement: Supplementary file 18 — Supplementary data file 13 [file 42003_2020_1060_MOESM18_ESM.zip › Additional_file_12/1059.pdf]

# Evolution of the gene family "724" ( $p=0.003$ )

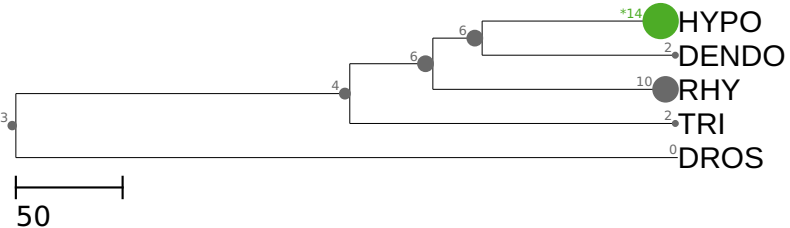

Supplement: Supplementary file 18 — Supplementary data file 13 [file 42003_2020_1060_MOESM18_ESM.zip › Additional_file_12/724.pdf]

# Evolution of the gene family "718" ( $p=0.023$ )

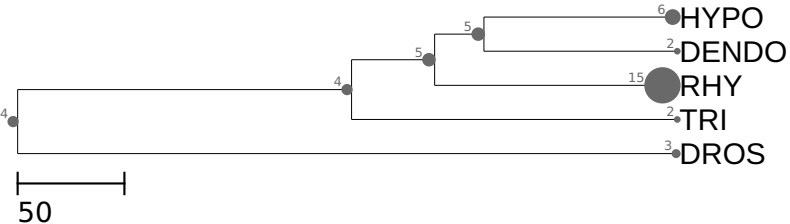

Supplement: Supplementary file 18 — Supplementary data file 13 [file 42003_2020_1060_MOESM18_ESM.zip › Additional_file_12/718.pdf]

Evolution of the gene family "3458" (p=0.039)

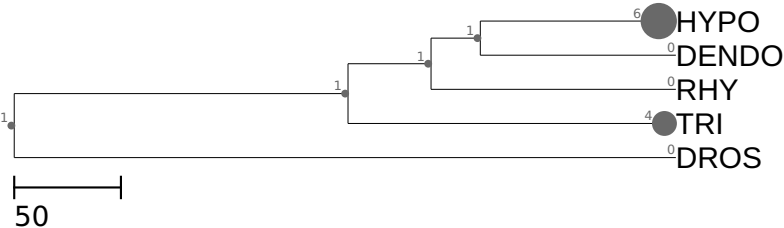

Supplement: Supplementary file 18 — Supplementary data file 13 [file 42003_2020_1060_MOESM18_ESM.zip › Additional_file_12/3458.pdf]

# Evolution of the gene family "136" ( $p=0.021$ )

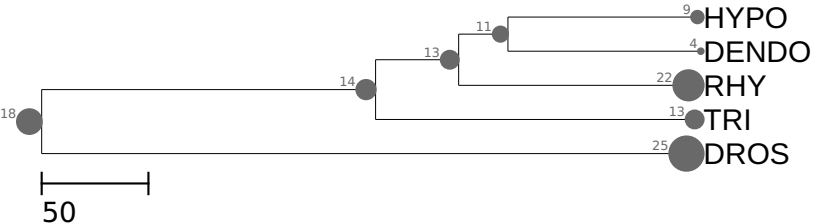

Supplement: Supplementary file 18 — Supplementary data file 13 [file 42003_2020_1060_MOESM18_ESM.zip › Additional_file_12/136.pdf]

# Evolution of the gene family "23" ( $p=0.019$ )

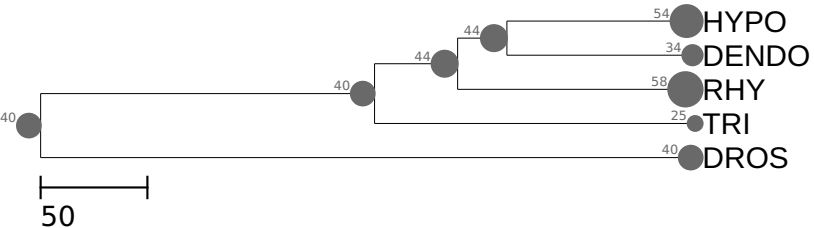

Supplement: Supplementary file 18 — Supplementary data file 13 [file 42003_2020_1060_MOESM18_ESM.zip › Additional_file_12/23.pdf]

# Evolution of the gene family "863" ( $p=0.024$ )

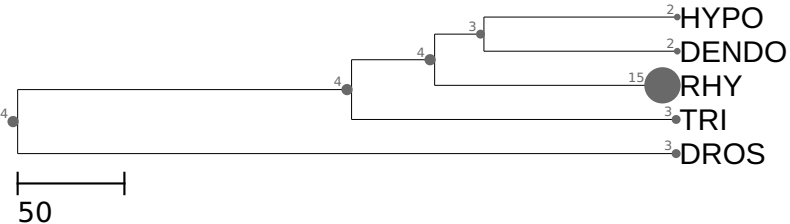

Supplement: Supplementary file 18 — Supplementary data file 13 [file 42003_2020_1060_MOESM18_ESM.zip › Additional_file_12/863.pdf]

# Evolution of the gene family "37" ( $p=0.007$ )

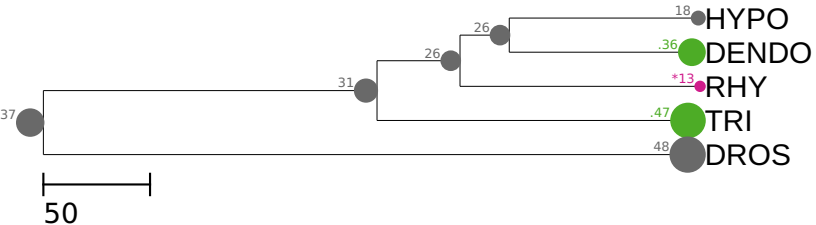

Supplement: Supplementary file 18 — Supplementary data file 13 [file 42003_2020_1060_MOESM18_ESM.zip › Additional_file_12/37.pdf]

# Evolution of the gene family "308" ( $p=0.001$ )

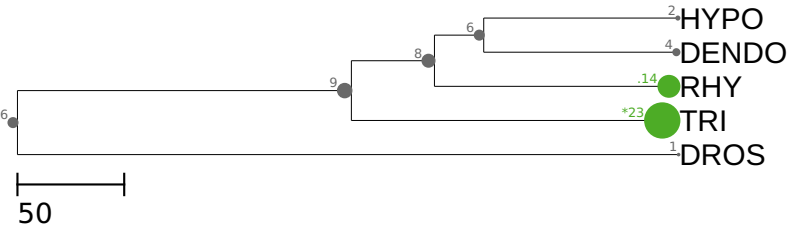

Supplement: Supplementary file 18 — Supplementary data file 13 [file 42003_2020_1060_MOESM18_ESM.zip › Additional_file_12/308.pdf]

# Evolution of the gene family "1892" ( $p=0.024$ )

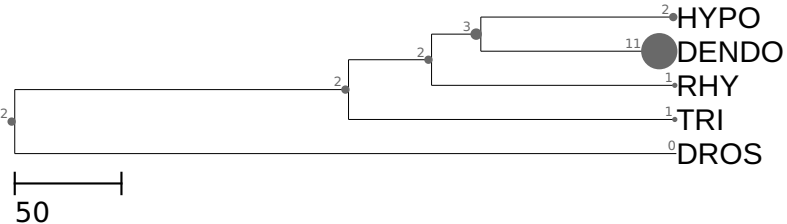

Supplement: Supplementary file 18 — Supplementary data file 13 [file 42003_2020_1060_MOESM18_ESM.zip › Additional_file_12/1892.pdf]

# Evolution of the gene family "1845" ( $p=0.048$ )

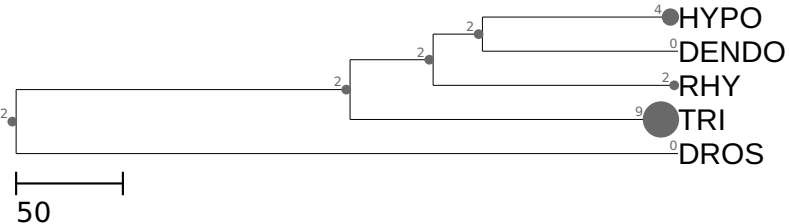

Supplement: Supplementary file 18 — Supplementary data file 13 [file 42003_2020_1060_MOESM18_ESM.zip › Additional_file_12/1845.pdf]

# Evolution of the gene family "453" ( $p=0.026$ )

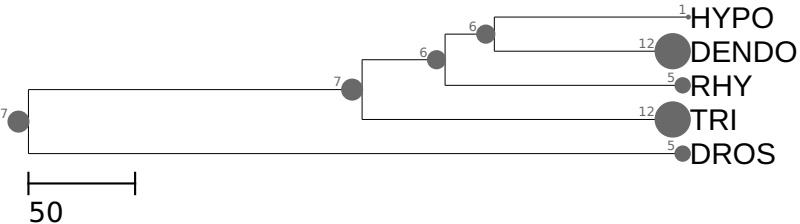

Supplement: Supplementary file 18 — Supplementary data file 13 [file 42003_2020_1060_MOESM18_ESM.zip › Additional_file_12/453.pdf]

# Evolution of the gene family "447" ( $p=0.007$ )

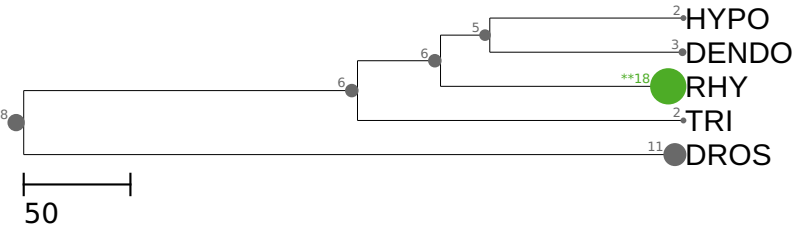

Supplement: Supplementary file 18 — Supplementary data file 13 [file 42003_2020_1060_MOESM18_ESM.zip › Additional_file_12/447.pdf]

# Evolution of the gene family "692" ( $p=0.009$ )

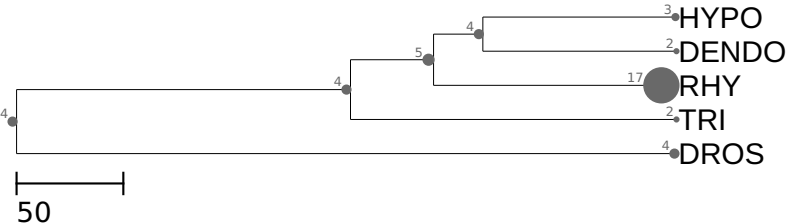

Supplement: Supplementary file 18 — Supplementary data file 13 [file 42003_2020_1060_MOESM18_ESM.zip › Additional_file_12/692.pdf]

## Evolution of the gene family "731" (p=0.009)

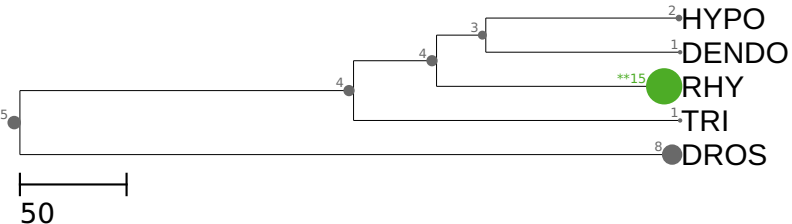

Supplement: Supplementary file 18 — Supplementary data file 13 [file 42003_2020_1060_MOESM18_ESM.zip › Additional_file_12/731.pdf]

Evolution of the gene family "2209" (p=0.012)

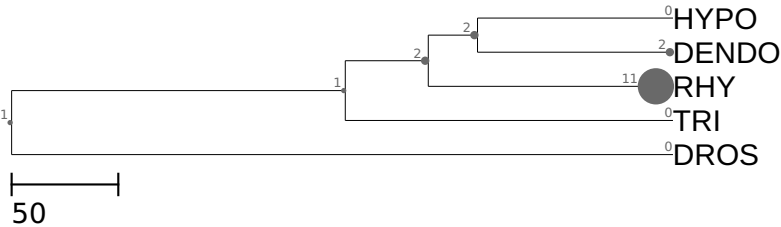

Supplement: Supplementary file 18 — Supplementary data file 13 [file 42003_2020_1060_MOESM18_ESM.zip › Additional_file_12/2209.pdf]

Evolution of the gene family "3895" (p=0.036)

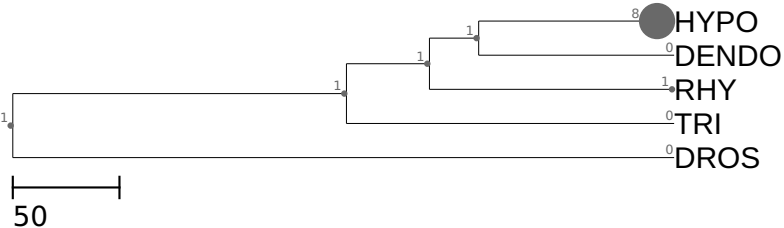

Supplement: Supplementary file 18 — Supplementary data file 13 [file 42003_2020_1060_MOESM18_ESM.zip › Additional_file_12/3895.pdf]

# Evolution of the gene family "1270" ( $p=0.024$ )

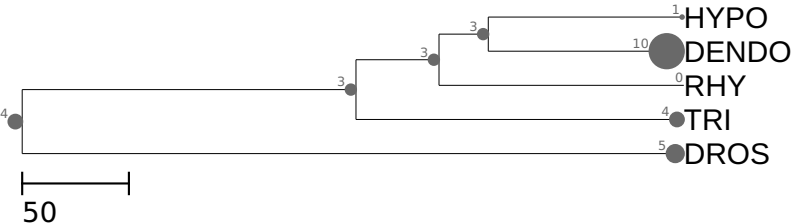

Supplement: Supplementary file 18 — Supplementary data file 13 [file 42003_2020_1060_MOESM18_ESM.zip › Additional_file_12/1270.pdf]

# Evolution of the gene family "647" ( $p=0.019$ )

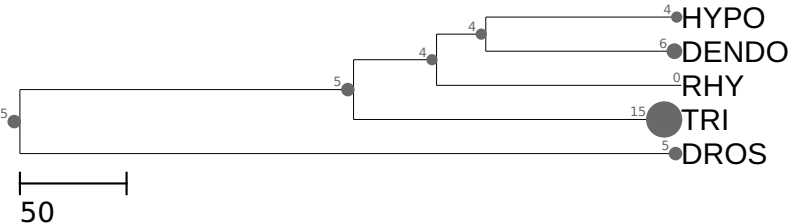

Supplement: Supplementary file 18 — Supplementary data file 13 [file 42003_2020_1060_MOESM18_ESM.zip › Additional_file_12/647.pdf]

# Evolution of the gene family "109" ( $p=0.024$ )

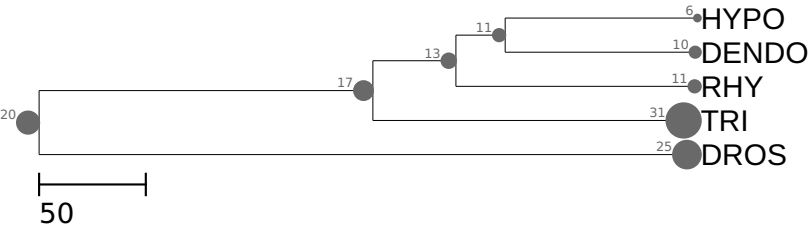

Supplement: Supplementary file 18 — Supplementary data file 13 [file 42003_2020_1060_MOESM18_ESM.zip › Additional_file_12/109.pdf]

# Evolution of the gene family "34" ( $p=0.029$ )

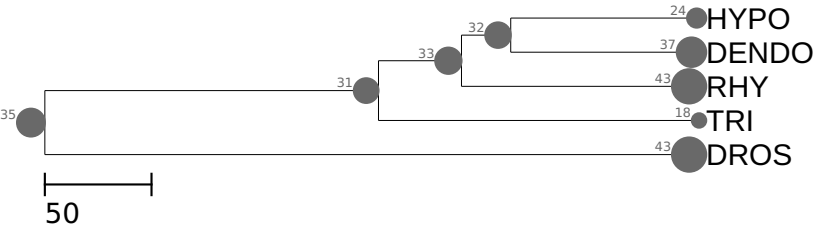

Supplement: Supplementary file 18 — Supplementary data file 13 [file 42003_2020_1060_MOESM18_ESM.zip › Additional_file_12/34.pdf]

# Evolution of the gene family "445" ( $p=0.028$ )

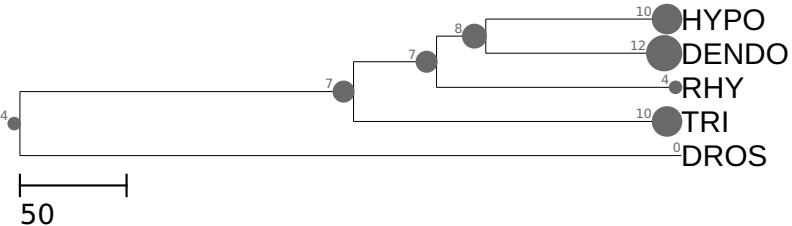

Supplement: Supplementary file 18 — Supplementary data file 13 [file 42003_2020_1060_MOESM18_ESM.zip › Additional_file_12/445.pdf]

# Evolution of the gene family "323" ( $p=0.002$ )

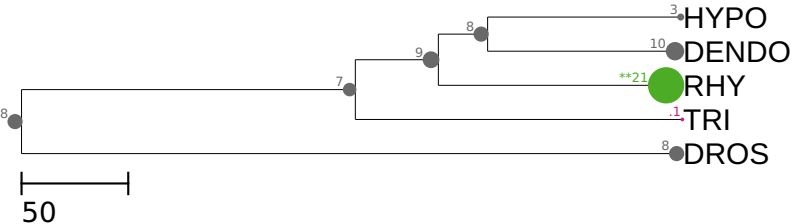

Supplement: Supplementary file 18 — Supplementary data file 13 [file 42003_2020_1060_MOESM18_ESM.zip › Additional_file_12/323.pdf]

# Evolution of the gene family "492" ( $p=0.001$ )

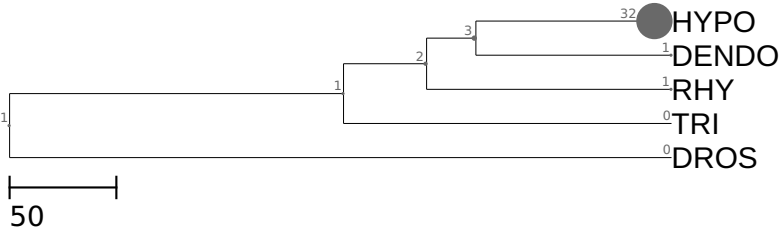

Supplement: Supplementary file 18 — Supplementary data file 13 [file 42003_2020_1060_MOESM18_ESM.zip › Additional_file_12/492.pdf]

# Evolution of the gene family "1885" ( $p=0.023$ )

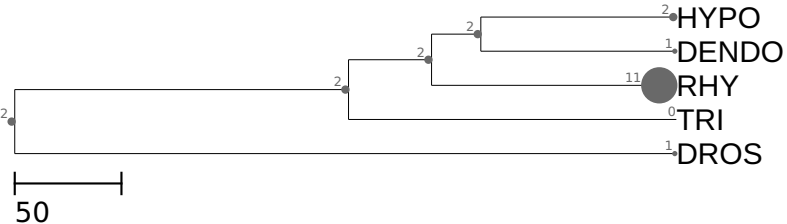

Supplement: Supplementary file 18 — Supplementary data file 13 [file 42003_2020_1060_MOESM18_ESM.zip › Additional_file_12/1885.pdf]

# Evolution of the gene family "336" ( $p=0.05$ )

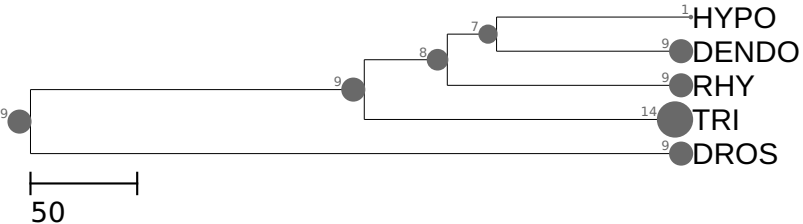

Supplement: Supplementary file 18 — Supplementary data file 13 [file 42003_2020_1060_MOESM18_ESM.zip › Additional_file_12/336.pdf]

Evolution of the gene family "450" (p=0.0)

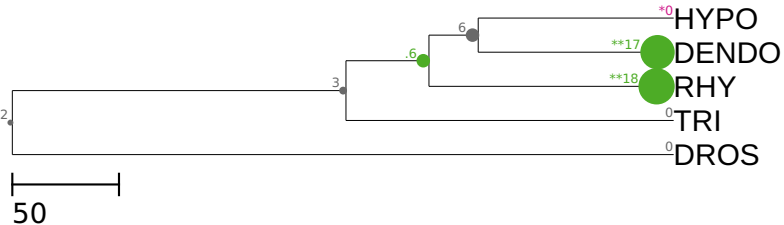

Supplement: Supplementary file 18 — Supplementary data file 13 [file 42003_2020_1060_MOESM18_ESM.zip › Additional_file_12/450.pdf]

# Evolution of the gene family "21" ( $p=0.027$ )

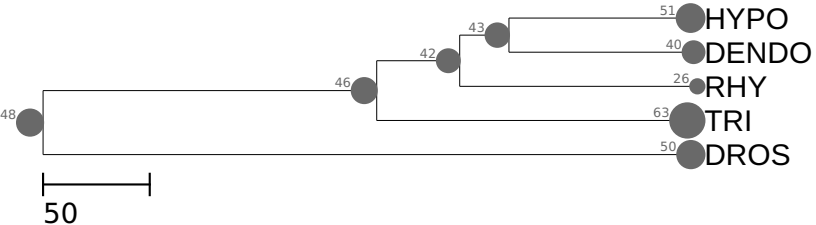

Supplement: Supplementary file 18 — Supplementary data file 13 [file 42003_2020_1060_MOESM18_ESM.zip › Additional_file_12/21.pdf]

Evolution of the gene family "1339" (p=0.01)

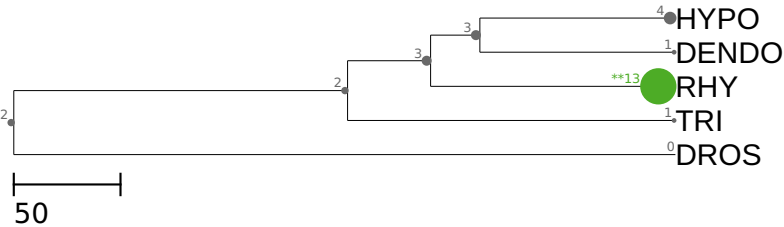

Supplement: Supplementary file 18 — Supplementary data file 13 [file 42003_2020_1060_MOESM18_ESM.zip › Additional_file_12/1339.pdf]

Evolution of the gene family "685" (p=0.0)

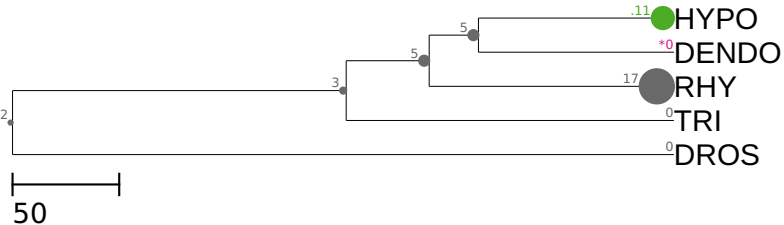

Supplement: Supplementary file 18 — Supplementary data file 13 [file 42003_2020_1060_MOESM18_ESM.zip › Additional_file_12/685.pdf]

Evolution of the gene family "732" (p=0.0)

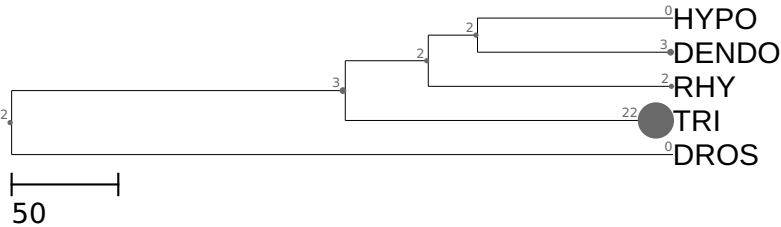

Supplement: Supplementary file 18 — Supplementary data file 13 [file 42003_2020_1060_MOESM18_ESM.zip › Additional_file_12/732.pdf]

# Evolution of the gene family "1932" ( $p=0.026$ )

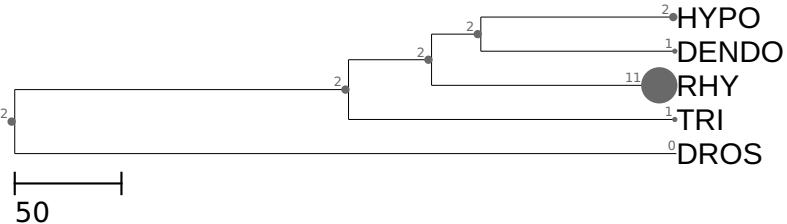

Supplement: Supplementary file 18 — Supplementary data file 13 [file 42003_2020_1060_MOESM18_ESM.zip › Additional_file_12/1932.pdf]

# Evolution of the gene family "530" ( $p=0.001$ )

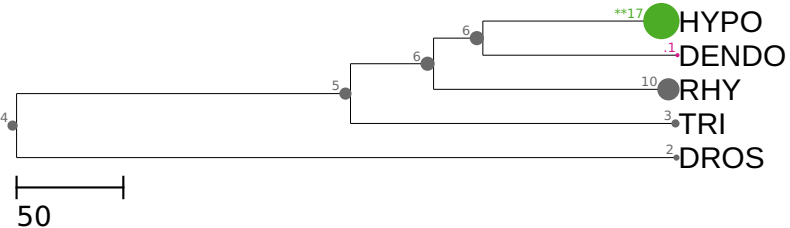

Supplement: Supplementary file 18 — Supplementary data file 13 [file 42003_2020_1060_MOESM18_ESM.zip › Additional_file_12/530.pdf]

# Evolution of the gene family "524" ( $p=0.001$ )

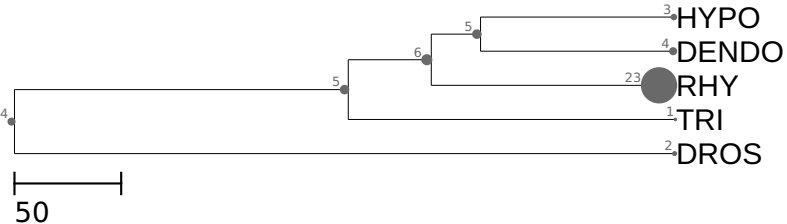

Supplement: Supplementary file 18 — Supplementary data file 13 [file 42003_2020_1060_MOESM18_ESM.zip › Additional_file_12/524.pdf]

# Evolution of the gene family "1077" ( $p=0.009$ )

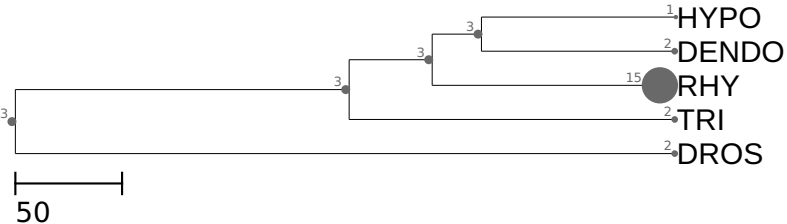

Supplement: Supplementary file 18 — Supplementary data file 13 [file 42003_2020_1060_MOESM18_ESM.zip › Additional_file_12/1077.pdf]

Evolution of the gene family "291" (p=0.003)

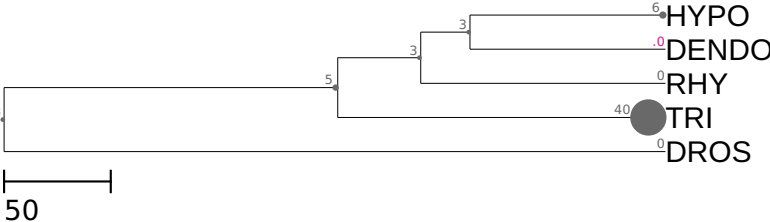

Supplement: Supplementary file 18 — Supplementary data file 13 [file 42003_2020_1060_MOESM18_ESM.zip › Additional_file_12/291.pdf]

# Evolution of the gene family "905" ( $p=0.039$ )

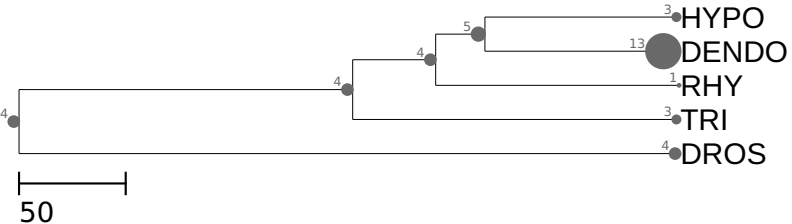

Supplement: Supplementary file 18 — Supplementary data file 13 [file 42003_2020_1060_MOESM18_ESM.zip › Additional_file_12/905.pdf]

# Evolution of the gene family "1249" (p=0.018)

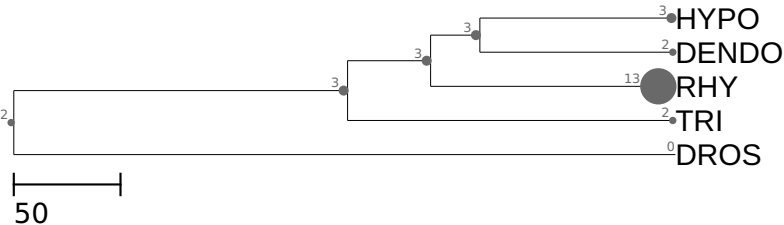

Supplement: Supplementary file 18 — Supplementary data file 13 [file 42003_2020_1060_MOESM18_ESM.zip › Additional_file_12/1249.pdf]

# Evolution of the gene family "124" ( $p=0.003$ )

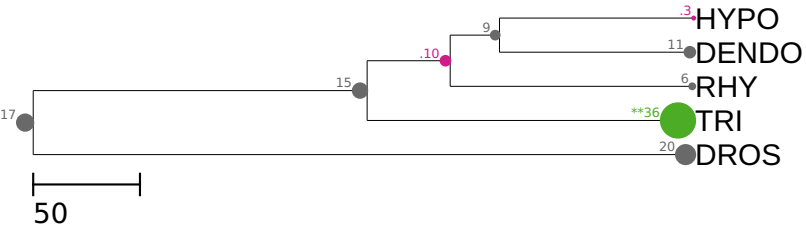

Supplement: Supplementary file 18 — Supplementary data file 13 [file 42003_2020_1060_MOESM18_ESM.zip › Additional_file_12/124.pdf]

# Evolution of the gene family "1671" ( $p=0.049$ )

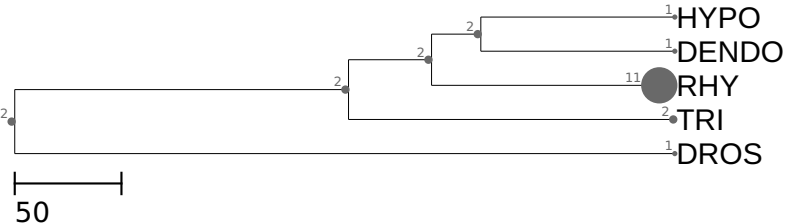

Supplement: Supplementary file 18 — Supplementary data file 13 [file 42003_2020_1060_MOESM18_ESM.zip › Additional_file_12/1671.pdf]

# Evolution of the gene family "483" ( $p=0.011$ )

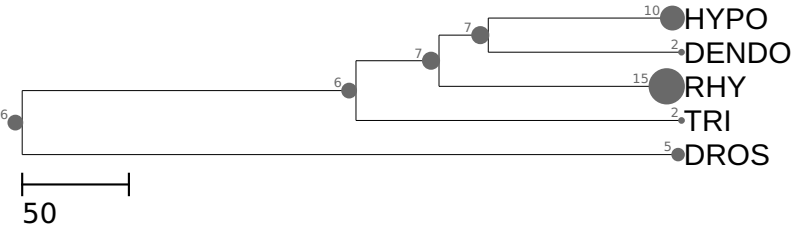

Supplement: Supplementary file 18 — Supplementary data file 13 [file 42003_2020_1060_MOESM18_ESM.zip › Additional_file_12/483.pdf]

# Evolution of the gene family "327" ( $p=0.023$ )

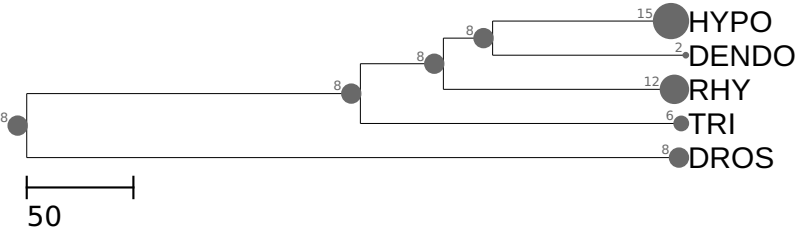

Supplement: Supplementary file 18 — Supplementary data file 13 [file 42003_2020_1060_MOESM18_ESM.zip › Additional_file_12/327.pdf]

# Evolution of the gene family "441" ( $p=0.05$ )

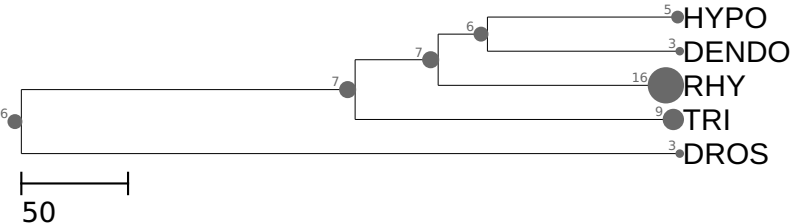

Supplement: Supplementary file 18 — Supplementary data file 13 [file 42003_2020_1060_MOESM18_ESM.zip › Additional_file_12/441.pdf]
