# Supplementary figures and images for: The genome of pest Rhynchophorus ferrugineus reveals gene families important at the plant-beetle interface (part 1 of 2)
Source: Commun Biol. 2020 Jun 24;3:323. doi: 10.1038/s42003-020-1060-8 (PMC7314810; doi:10.1038/s42003-020-1060-8)

# Evolution of the gene family "202" ( $p=0.012$ )

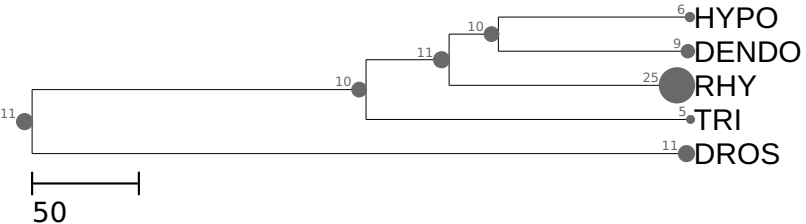

Supplement: Supplementary file 18 — Supplementary data file 13 [file 42003_2020_1060_MOESM18_ESM.zip › Additional_file_12/202.pdf]

Evolution of the gene family "2248" (p=0.012)

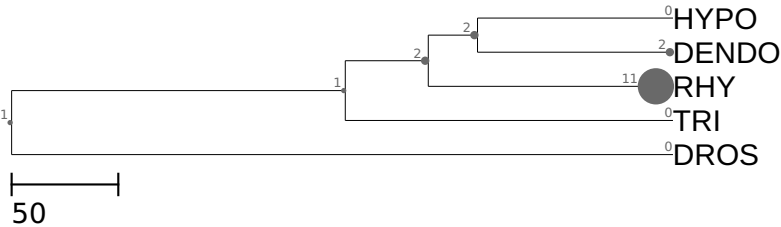

Supplement: Supplementary file 18 — Supplementary data file 13 [file 42003_2020_1060_MOESM18_ESM.zip › Additional_file_12/2248.pdf]

# Evolution of the gene family "1741" ( $p=0.025$ )

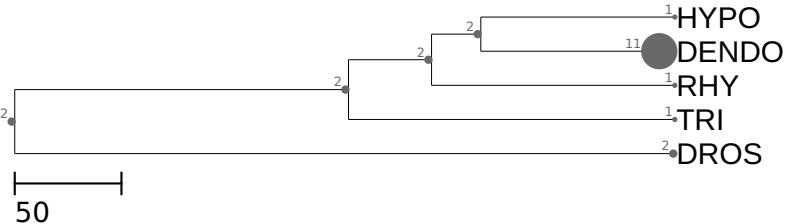

Supplement: Supplementary file 18 — Supplementary data file 13 [file 42003_2020_1060_MOESM18_ESM.zip › Additional_file_12/1741.pdf]

Evolution of the gene family "2274" (p=0.023)

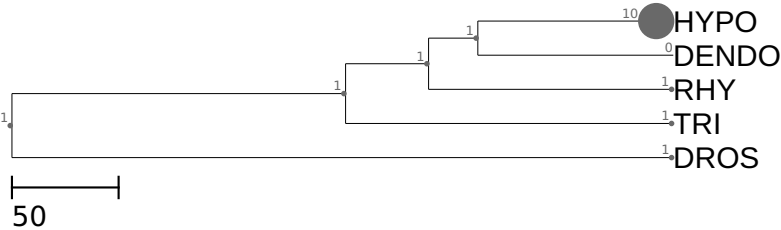

Supplement: Supplementary file 18 — Supplementary data file 13 [file 42003_2020_1060_MOESM18_ESM.zip › Additional_file_12/2274.pdf]

# Evolution of the gene family "61" ( $p=0.007$ )

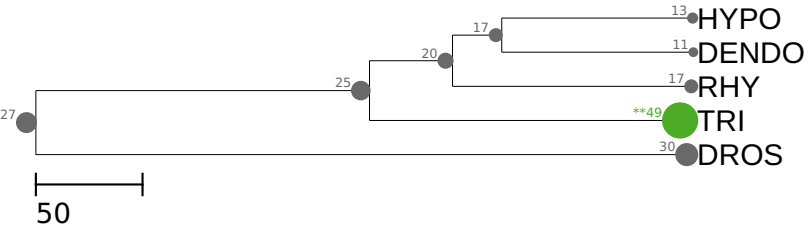

Supplement: Supplementary file 18 — Supplementary data file 13 [file 42003_2020_1060_MOESM18_ESM.zip › Additional_file_12/61.pdf]

Evolution of the gene family "821" (p=0.0)

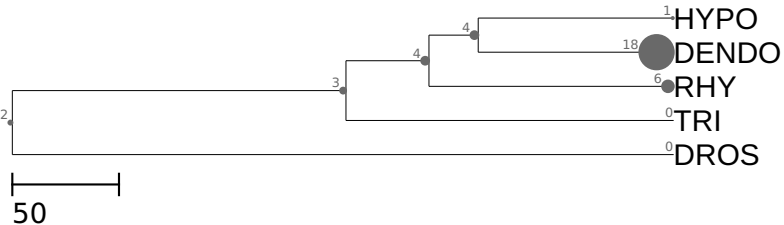

Supplement: Supplementary file 18 — Supplementary data file 13 [file 42003_2020_1060_MOESM18_ESM.zip › Additional_file_12/821.pdf]

# Evolution of the gene family "75" ( $p=0.009$ )

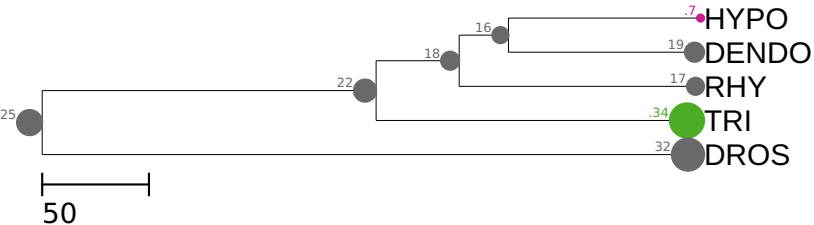

Supplement: Supplementary file 18 — Supplementary data file 13 [file 42003_2020_1060_MOESM18_ESM.zip › Additional_file_12/75.pdf]

# Evolution of the gene family "404" ( $p=0.007$ )

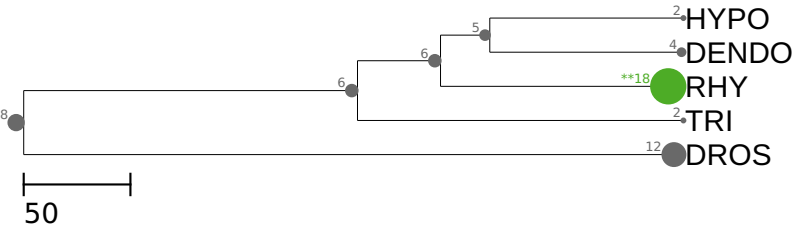

Supplement: Supplementary file 18 — Supplementary data file 13 [file 42003_2020_1060_MOESM18_ESM.zip › Additional_file_12/404.pdf]

# Evolution of the gene family "362" ( $p=0.05$ )

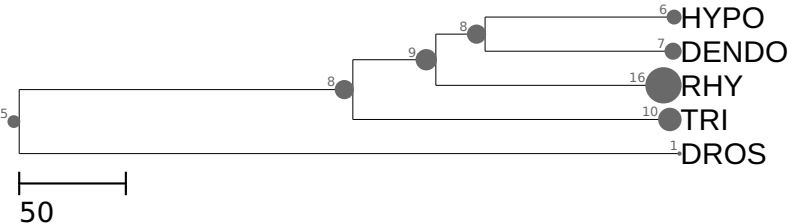

Supplement: Supplementary file 18 — Supplementary data file 13 [file 42003_2020_1060_MOESM18_ESM.zip › Additional_file_12/362.pdf]

# Evolution of the gene family "410" (p=0.0)

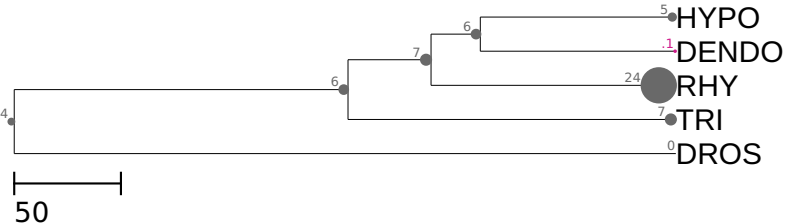

Supplement: Supplementary file 18 — Supplementary data file 13 [file 42003_2020_1060_MOESM18_ESM.zip › Additional_file_12/410.pdf]

# Evolution of the gene family "1146" (p=0.018)

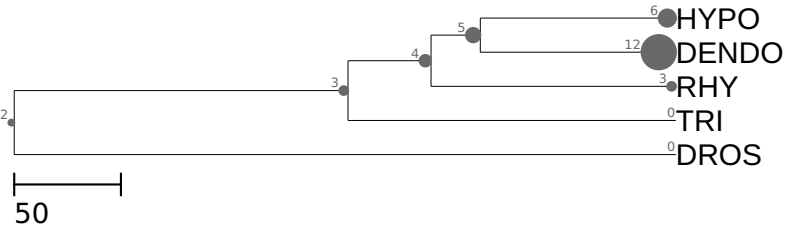

Supplement: Supplementary file 18 — Supplementary data file 13 [file 42003_2020_1060_MOESM18_ESM.zip › Additional_file_12/1146.pdf]

# Evolution of the gene family "411" ( $p=0.007$ )

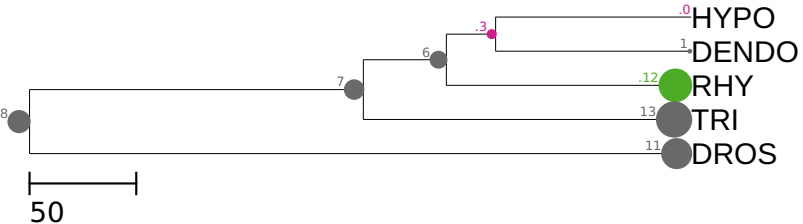

Supplement: Supplementary file 18 — Supplementary data file 13 [file 42003_2020_1060_MOESM18_ESM.zip › Additional_file_12/411.pdf]

# Evolution of the gene family "363" ( $p=0.013$ )

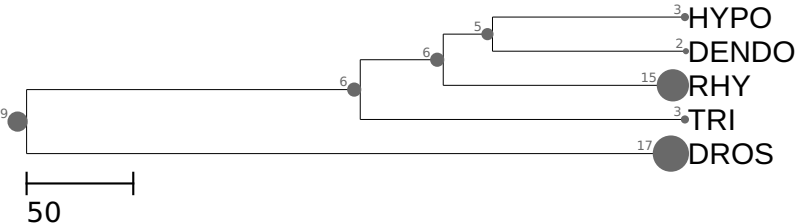

Supplement: Supplementary file 18 — Supplementary data file 13 [file 42003_2020_1060_MOESM18_ESM.zip › Additional_file_12/363.pdf]

# Evolution of the gene family "48" ( $p=0.007$ )

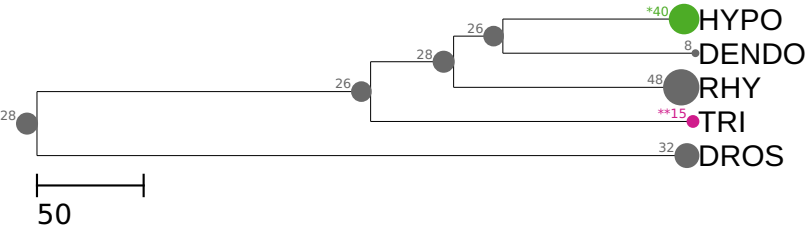

Supplement: Supplementary file 18 — Supplementary data file 13 [file 42003_2020_1060_MOESM18_ESM.zip › Additional_file_12/48.pdf]

Evolution of the gene family "1422" (p=0.002)

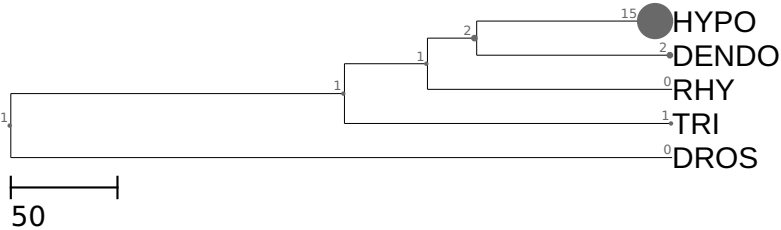

Supplement: Supplementary file 18 — Supplementary data file 13 [file 42003_2020_1060_MOESM18_ESM.zip › Additional_file_12/1422.pdf]

# Evolution of the gene family "613" ( $p=0.015$ )

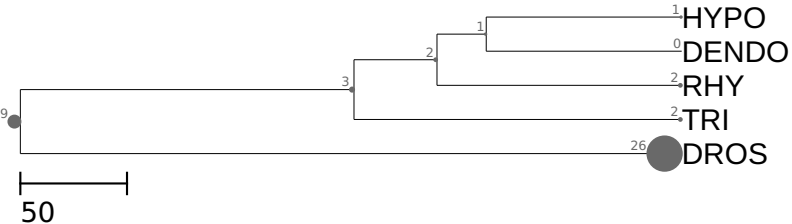

Supplement: Supplementary file 18 — Supplementary data file 13 [file 42003_2020_1060_MOESM18_ESM.zip › Additional_file_12/613.pdf]

# Evolution of the gene family "607" ( $p=0.041$ )

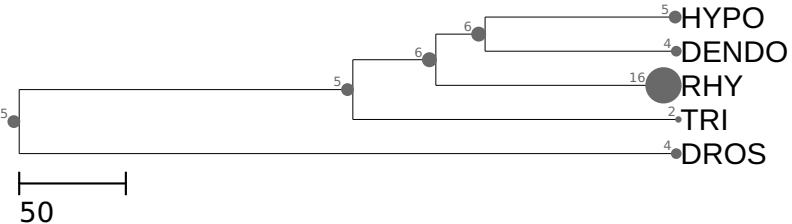

Supplement: Supplementary file 18 — Supplementary data file 13 [file 42003_2020_1060_MOESM18_ESM.zip › Additional_file_12/607.pdf]

# Evolution of the gene family "798" (p=0.0)

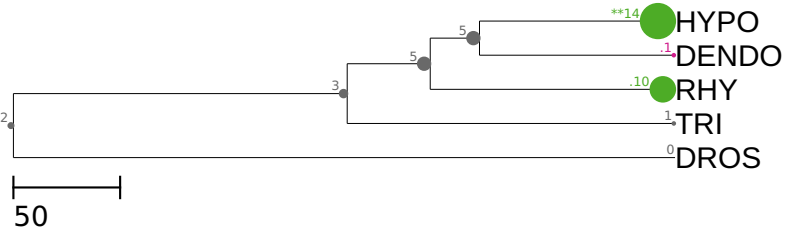

Supplement: Supplementary file 18 — Supplementary data file 13 [file 42003_2020_1060_MOESM18_ESM.zip › Additional_file_12/798.pdf]

# Evolution of the gene family "983" ( $p=0.042$ )

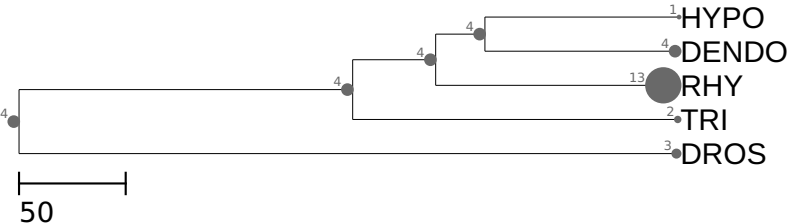

Supplement: Supplementary file 18 — Supplementary data file 13 [file 42003_2020_1060_MOESM18_ESM.zip › Additional_file_12/983.pdf]

# Evolution of the gene family "773" ( $p=0.005$ )

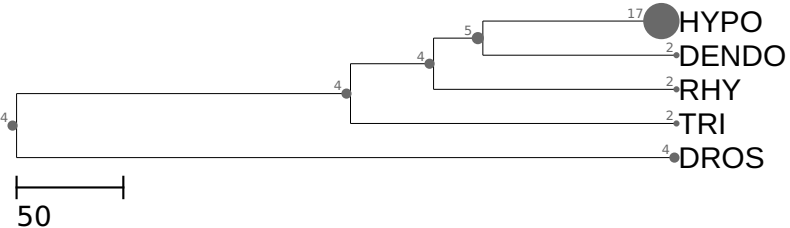

Supplement: Supplementary file 18 — Supplementary data file 13 [file 42003_2020_1060_MOESM18_ESM.zip › Additional_file_12/773.pdf]

# Evolution of the gene family "1768" ( $p=0.03$ )

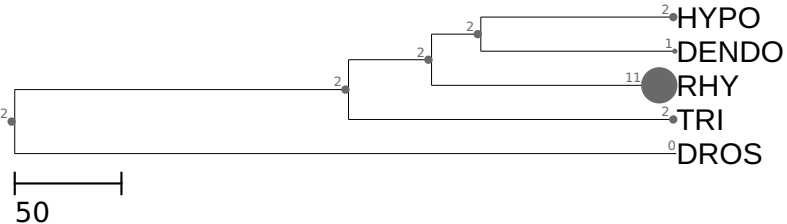

Supplement: Supplementary file 18 — Supplementary data file 13 [file 42003_2020_1060_MOESM18_ESM.zip › Additional_file_12/1768.pdf]

# Evolution of the gene family "2261" ( $p=0.036$ )

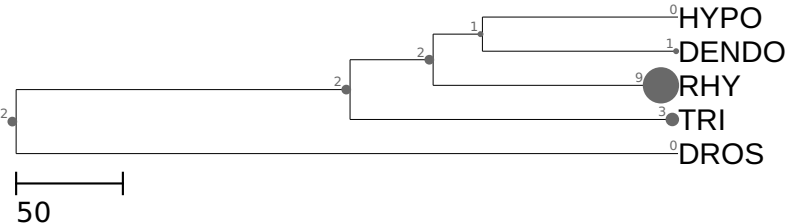

Supplement: Supplementary file 18 — Supplementary data file 13 [file 42003_2020_1060_MOESM18_ESM.zip › Additional_file_12/2261.pdf]

# Evolution of the gene family "1032" ( $p=0.009$ )

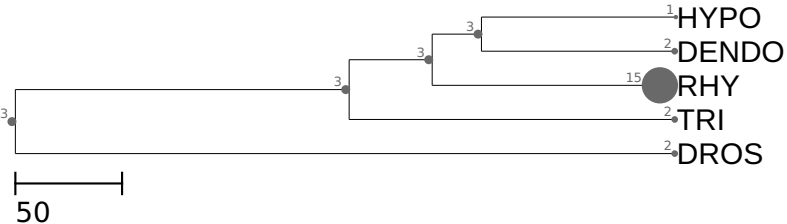

Supplement: Supplementary file 18 — Supplementary data file 13 [file 42003_2020_1060_MOESM18_ESM.zip › Additional_file_12/1032.pdf]

# Evolution of the gene family "571" ( $p=0.031$ )

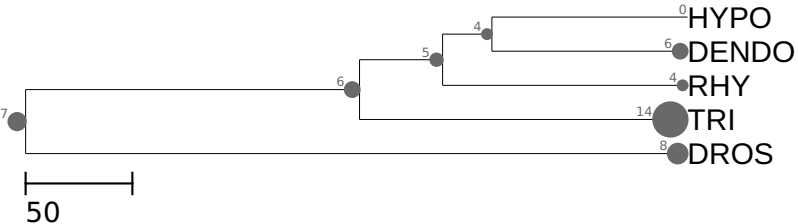

Supplement: Supplementary file 18 — Supplementary data file 13 [file 42003_2020_1060_MOESM18_ESM.zip › Additional_file_12/571.pdf]

# Evolution of the gene family "573" ( $p=0.026$ )

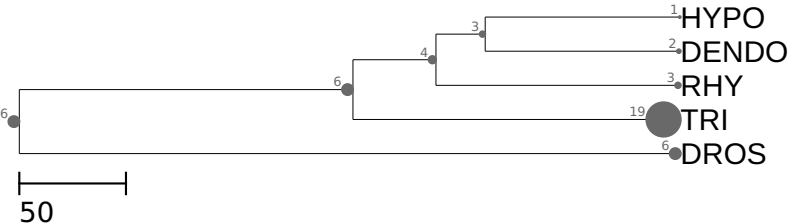

Supplement: Supplementary file 18 — Supplementary data file 13 [file 42003_2020_1060_MOESM18_ESM.zip › Additional_file_12/573.pdf]

# Evolution of the gene family "1583" ( $p=0.037$ )

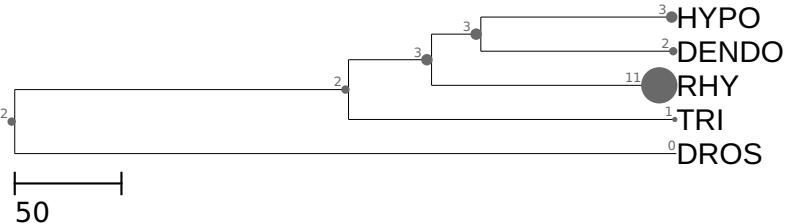

Supplement: Supplementary file 18 — Supplementary data file 13 [file 42003_2020_1060_MOESM18_ESM.zip › Additional_file_12/1583.pdf]

# Evolution of the gene family "1232" ( $p=0.044$ )

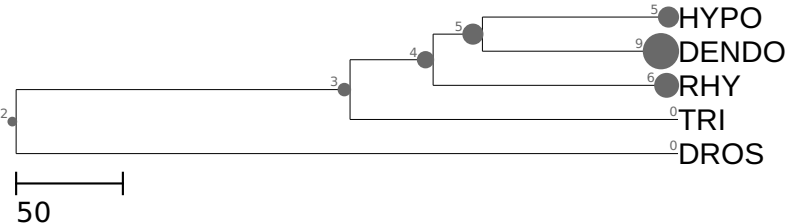

Supplement: Supplementary file 18 — Supplementary data file 13 [file 42003_2020_1060_MOESM18_ESM.zip › Additional_file_12/1232.pdf]

Evolution of the gene family "942" (p=0.0)

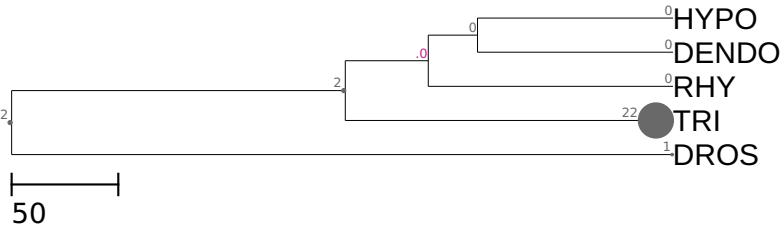

Supplement: Supplementary file 18 — Supplementary data file 13 [file 42003_2020_1060_MOESM18_ESM.zip › Additional_file_12/942.pdf]

# Evolution of the gene family "177" ( $p=0.041$ )

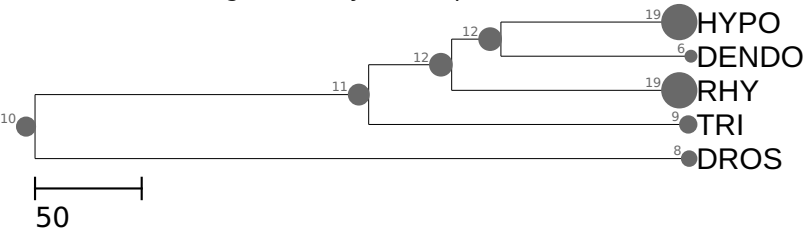

Supplement: Supplementary file 18 — Supplementary data file 13 [file 42003_2020_1060_MOESM18_ESM.zip › Additional_file_12/177.pdf]

# Evolution of the gene family "605" ( $p=0.001$ )

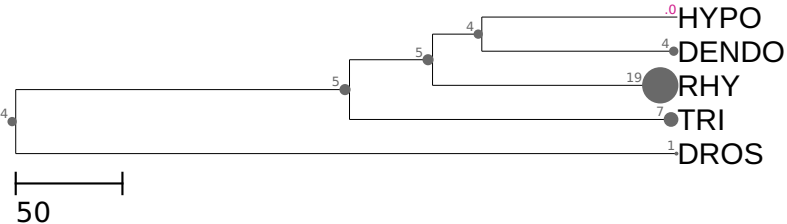

Supplement: Supplementary file 18 — Supplementary data file 13 [file 42003_2020_1060_MOESM18_ESM.zip › Additional_file_12/605.pdf]

# Evolution of the gene family "188" ( $p=0.033$ )

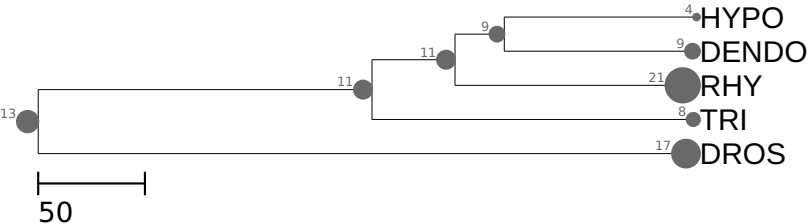

Supplement: Supplementary file 18 — Supplementary data file 13 [file 42003_2020_1060_MOESM18_ESM.zip › Additional_file_12/188.pdf]

# Evolution of the gene family "407" ( $p=0.004$ )

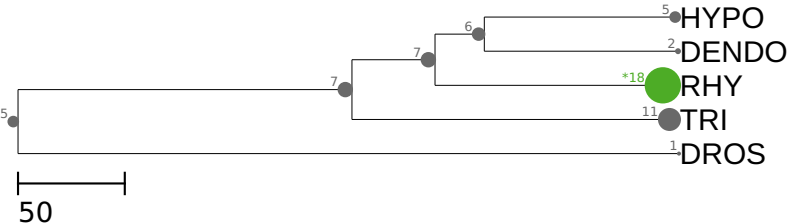

Supplement: Supplementary file 18 — Supplementary data file 13 [file 42003_2020_1060_MOESM18_ESM.zip › Additional_file_12/407.pdf]

Evolution of the gene family "1179" (p=0.003)

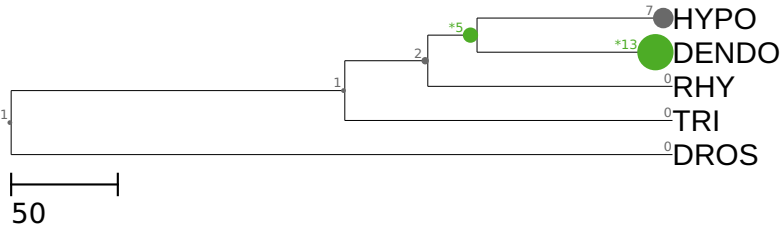

Supplement: Supplementary file 18 — Supplementary data file 13 [file 42003_2020_1060_MOESM18_ESM.zip › Additional_file_12/1179.pdf]

Evolution of the gene family "3008" (p=0.048)

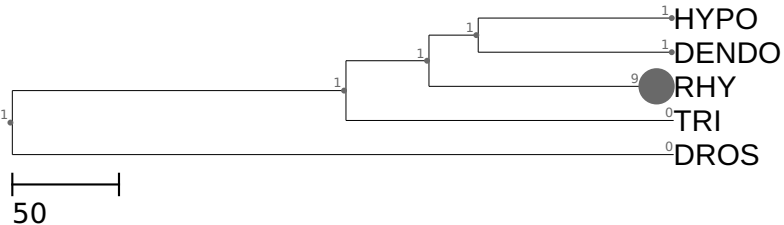

Supplement: Supplementary file 18 — Supplementary data file 13 [file 42003_2020_1060_MOESM18_ESM.zip › Additional_file_12/3008.pdf]

# Evolution of the gene family "348" ( $p=0.003$ )

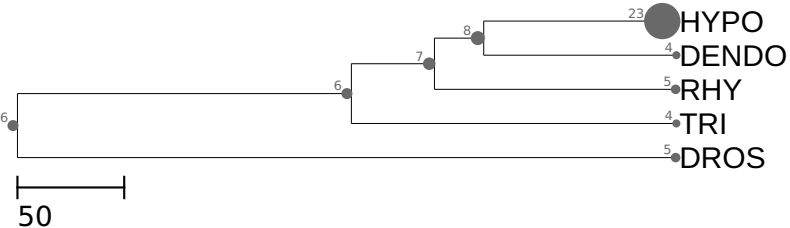

Supplement: Supplementary file 18 — Supplementary data file 13 [file 42003_2020_1060_MOESM18_ESM.zip › Additional_file_12/348.pdf]

# Evolution of the gene family "189" ( $p=0.002$ )

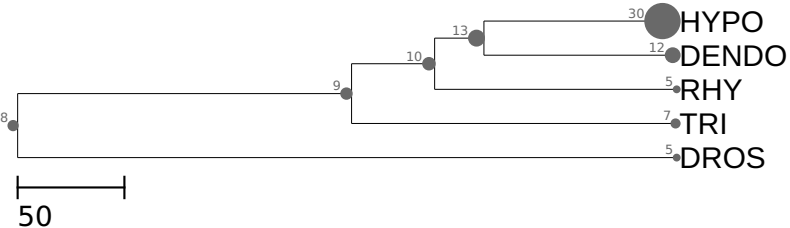

Supplement: Supplementary file 18 — Supplementary data file 13 [file 42003_2020_1060_MOESM18_ESM.zip › Additional_file_12/189.pdf]

Evolution of the gene family "1435" (p=0.008)

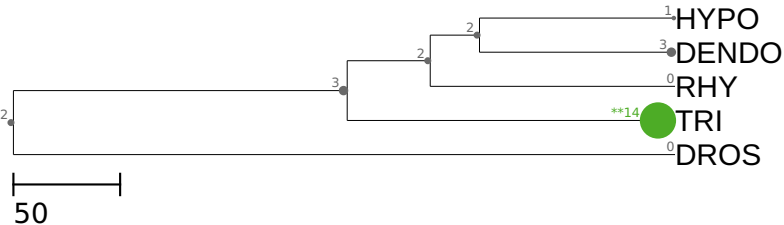

Supplement: Supplementary file 18 — Supplementary data file 13 [file 42003_2020_1060_MOESM18_ESM.zip › Additional_file_12/1435.pdf]

# Evolution of the gene family "638" ( $p=0.024$ )

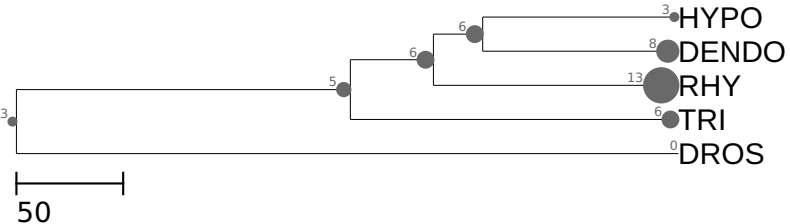

Supplement: Supplementary file 18 — Supplementary data file 13 [file 42003_2020_1060_MOESM18_ESM.zip › Additional_file_12/638.pdf]

# Evolution of the gene family "162" ( $p=0.001$ )

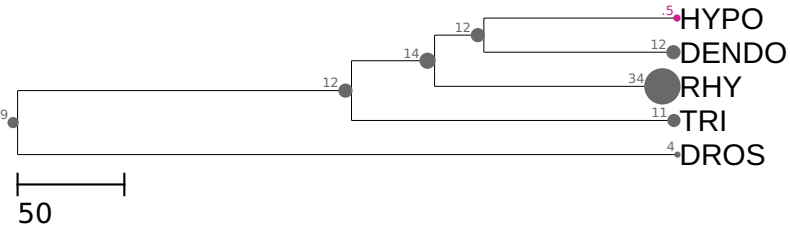

Supplement: Supplementary file 18 — Supplementary data file 13 [file 42003_2020_1060_MOESM18_ESM.zip › Additional_file_12/162.pdf]

Evolution of the gene family "1541" (p=0.013)

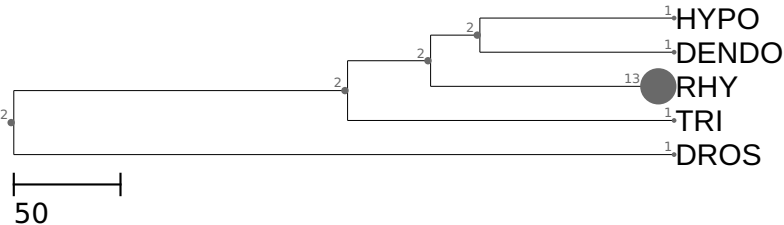

Supplement: Supplementary file 18 — Supplementary data file 13 [file 42003_2020_1060_MOESM18_ESM.zip › Additional_file_12/1541.pdf]

# Evolution of the gene family "770" ( $p=0.047$ )

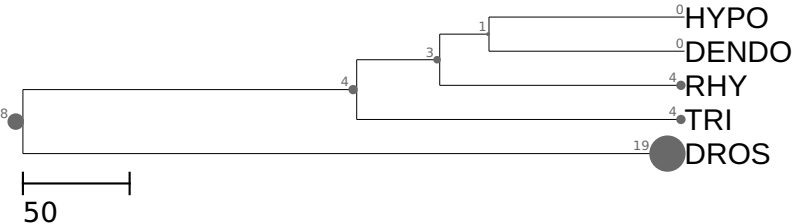

Supplement: Supplementary file 18 — Supplementary data file 13 [file 42003_2020_1060_MOESM18_ESM.zip › Additional_file_12/770.pdf]

Evolution of the gene family "1019" (p=0.04)

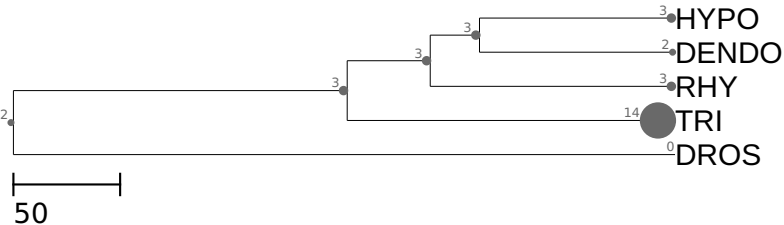

Supplement: Supplementary file 18 — Supplementary data file 13 [file 42003_2020_1060_MOESM18_ESM.zip › Additional_file_12/1019.pdf]

# Evolution of the gene family "599" ( $p=0.041$ )

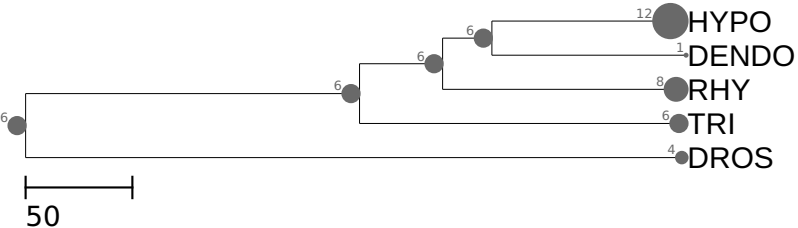

Supplement: Supplementary file 18 — Supplementary data file 13 [file 42003_2020_1060_MOESM18_ESM.zip › Additional_file_12/599.pdf]

Evolution of the gene family "228" (p=0.001)

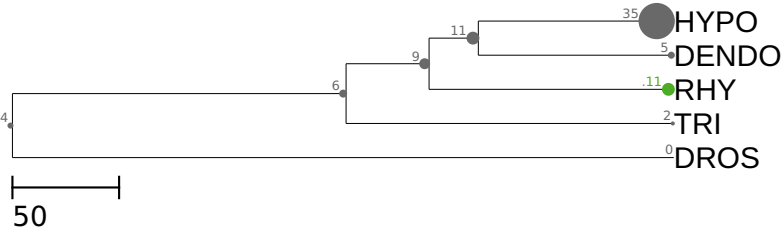

Supplement: Supplementary file 18 — Supplementary data file 13 [file 42003_2020_1060_MOESM18_ESM.zip › Additional_file_12/228.pdf]

# Evolution of the gene family "200" ( $p=0.004$ )

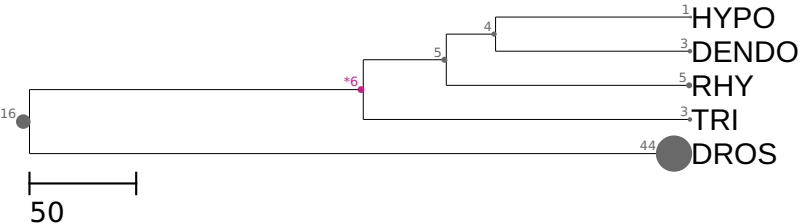

Supplement: Supplementary file 18 — Supplementary data file 13 [file 42003_2020_1060_MOESM18_ESM.zip › Additional_file_12/200.pdf]

# Evolution of the gene family "238" ( $p=0.012$ )

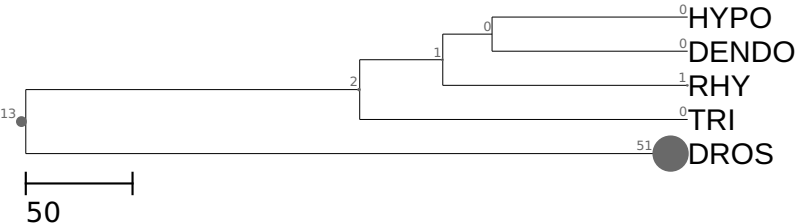

Supplement: Supplementary file 18 — Supplementary data file 13 [file 42003_2020_1060_MOESM18_ESM.zip › Additional_file_12/238.pdf]

Evolution of the gene family "1974" (p=0.023)

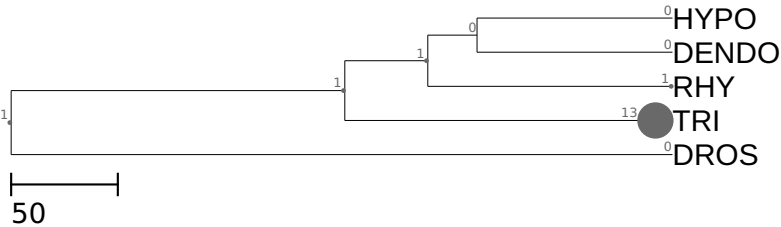

Supplement: Supplementary file 18 — Supplementary data file 13 [file 42003_2020_1060_MOESM18_ESM.zip › Additional_file_12/1974.pdf]

# Evolution of the gene family "210" ( $p=0.043$ )

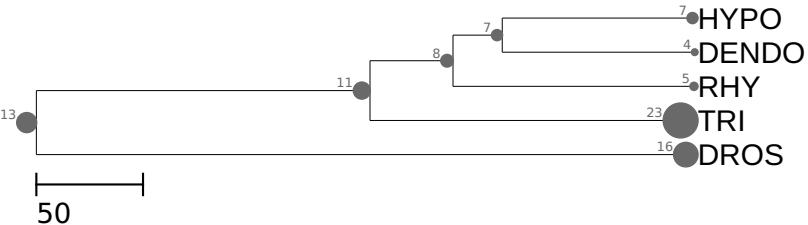

Supplement: Supplementary file 18 — Supplementary data file 13 [file 42003_2020_1060_MOESM18_ESM.zip › Additional_file_12/210.pdf]

# Evolution of the gene family "984" ( $p=0.009$ )

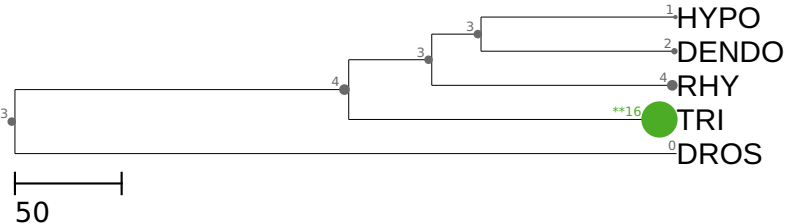

Supplement: Supplementary file 18 — Supplementary data file 13 [file 42003_2020_1060_MOESM18_ESM.zip › Additional_file_12/984.pdf]

Evolution of the gene family "3434" (p=0.041)

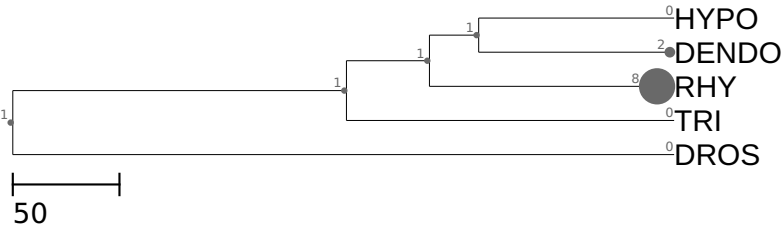

Supplement: Supplementary file 18 — Supplementary data file 13 [file 42003_2020_1060_MOESM18_ESM.zip › Additional_file_12/3434.pdf]

Evolution of the gene family "2689" (p=0.027)

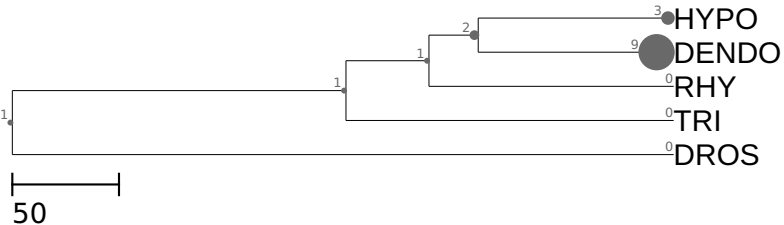

Supplement: Supplementary file 18 — Supplementary data file 13 [file 42003_2020_1060_MOESM18_ESM.zip › Additional_file_12/2689.pdf]

# Evolution of the gene family "166" ( $p=0.003$ )

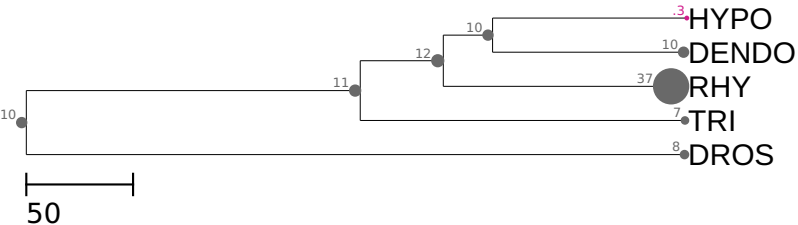

Supplement: Supplementary file 18 — Supplementary data file 13 [file 42003_2020_1060_MOESM18_ESM.zip › Additional_file_12/166.pdf]

# Evolution of the gene family "98" ( $p=0.002$ )

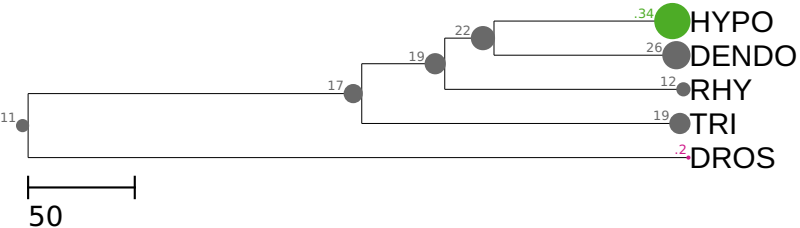

Supplement: Supplementary file 18 — Supplementary data file 13 [file 42003_2020_1060_MOESM18_ESM.zip › Additional_file_12/98.pdf]

# Evolution of the gene family "199" ( $p=0.003$ )

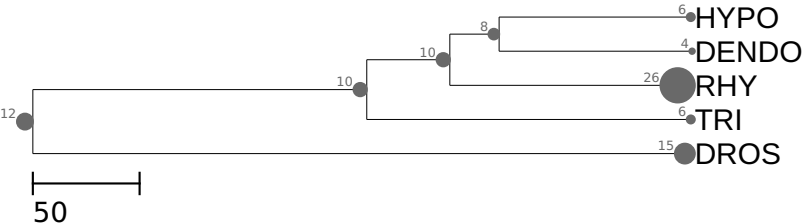

Supplement: Supplementary file 18 — Supplementary data file 13 [file 42003_2020_1060_MOESM18_ESM.zip › Additional_file_12/199.pdf]

# Evolution of the gene family "1425" ( $p=0.024$ )

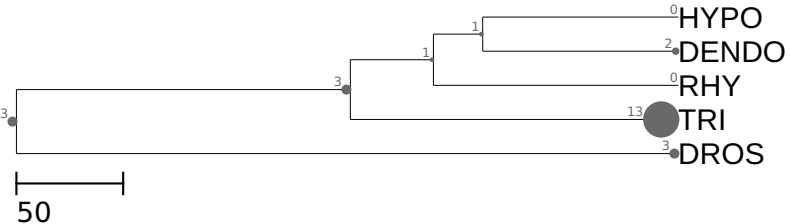

Supplement: Supplementary file 18 — Supplementary data file 13 [file 42003_2020_1060_MOESM18_ESM.zip › Additional_file_12/1425.pdf]

# Evolution of the gene family "370" ( $p=0.011$ )

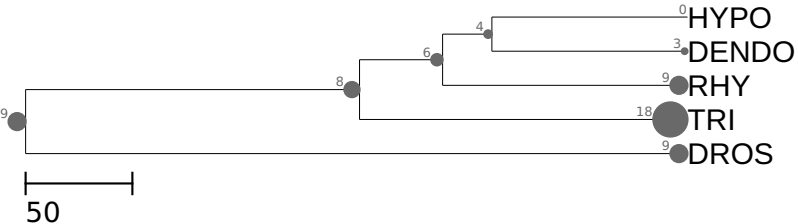

Supplement: Supplementary file 18 — Supplementary data file 13 [file 42003_2020_1060_MOESM18_ESM.zip › Additional_file_12/370.pdf]

Evolution of the gene family "1140" (p=0.002)

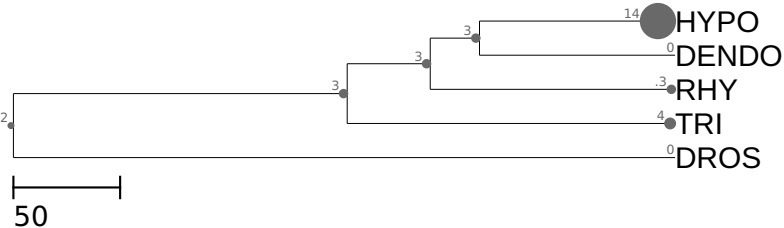

Supplement: Supplementary file 18 — Supplementary data file 13 [file 42003_2020_1060_MOESM18_ESM.zip › Additional_file_12/1140.pdf]

# Evolution of the gene family "371" ( $p=0.02$ )

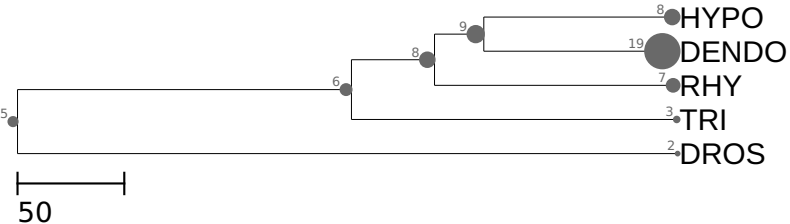

Supplement: Supplementary file 18 — Supplementary data file 13 [file 42003_2020_1060_MOESM18_ESM.zip › Additional_file_12/371.pdf]

Evolution of the gene family "1424" (p=0.028)

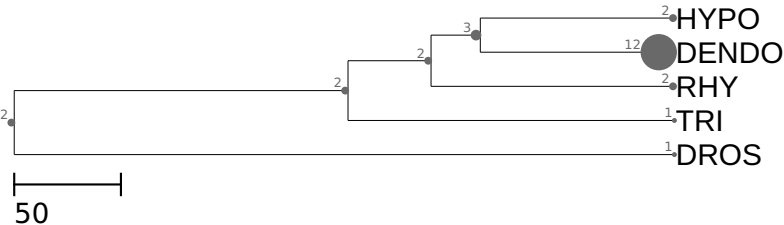

Supplement: Supplementary file 18 — Supplementary data file 13 [file 42003_2020_1060_MOESM18_ESM.zip › Additional_file_12/1424.pdf]

# Evolution of the gene family "198" ( $p=0.003$ )

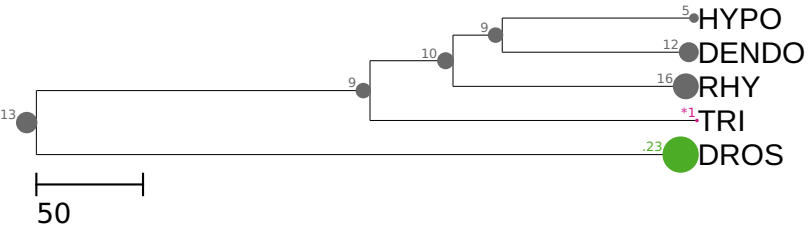

Supplement: Supplementary file 18 — Supplementary data file 13 [file 42003_2020_1060_MOESM18_ESM.zip › Additional_file_12/198.pdf]

Evolution of the gene family "832" (p=0.0)

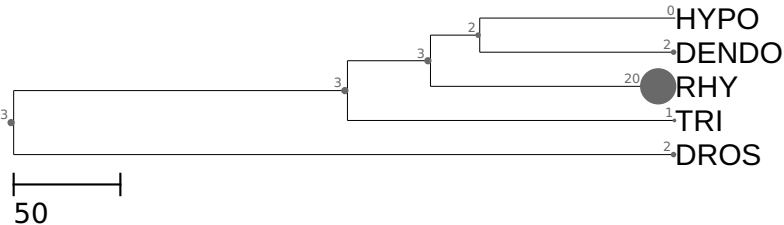

Supplement: Supplementary file 18 — Supplementary data file 13 [file 42003_2020_1060_MOESM18_ESM.zip › Additional_file_12/832.pdf]

# Evolution of the gene family "72" ( $p=0.008$ )

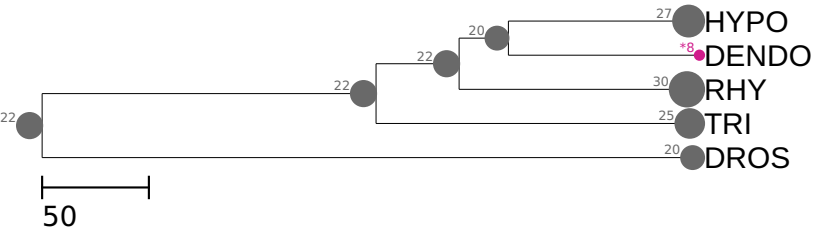

Supplement: Supplementary file 18 — Supplementary data file 13 [file 42003_2020_1060_MOESM18_ESM.zip › Additional_file_12/72.pdf]

# Evolution of the gene family "173" ( $p=0.039$ )

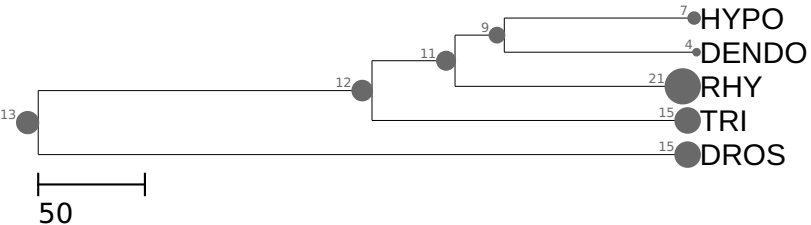

Supplement: Supplementary file 18 — Supplementary data file 13 [file 42003_2020_1060_MOESM18_ESM.zip › Additional_file_12/173.pdf]

# Evolution of the gene family "629" ( $p=0.001$ )

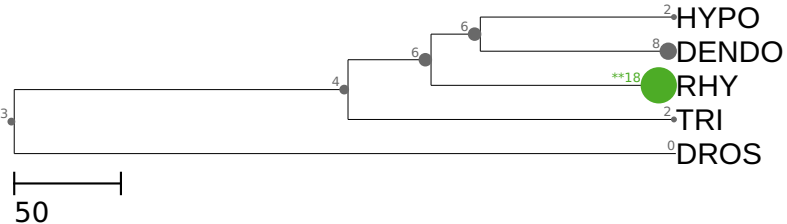

Supplement: Supplementary file 18 — Supplementary data file 13 [file 42003_2020_1060_MOESM18_ESM.zip › Additional_file_12/629.pdf]

# Evolution of the gene family "1746" ( $p=0.026$ )

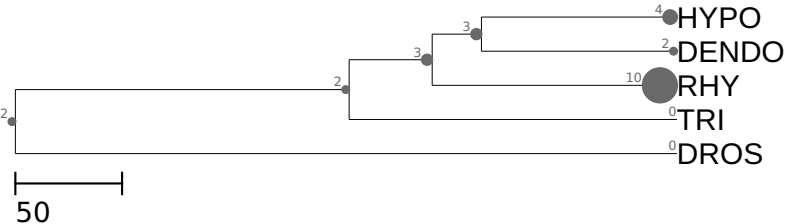

Supplement: Supplementary file 18 — Supplementary data file 13 [file 42003_2020_1060_MOESM18_ESM.zip › Additional_file_12/1746.pdf]

# Evolution of the gene family "588" ( $p=0.04$ )

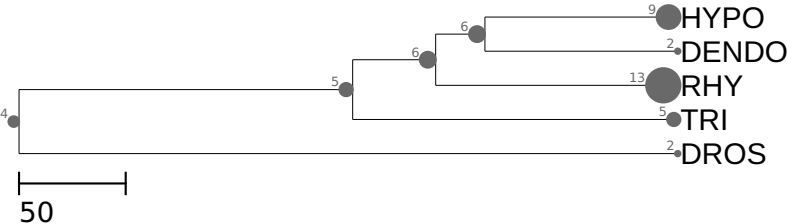

Supplement: Supplementary file 18 — Supplementary data file 13 [file 42003_2020_1060_MOESM18_ESM.zip › Additional_file_12/588.pdf]

Evolution of the gene family "2273" (p=0.003)

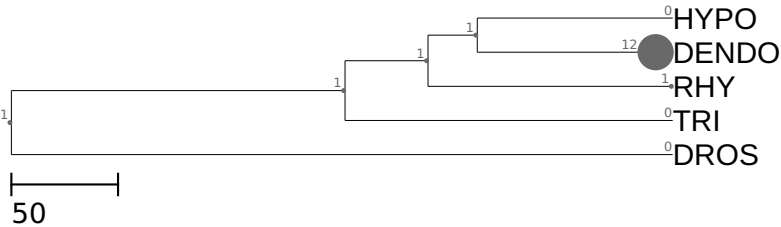

Supplement: Supplementary file 18 — Supplementary data file 13 [file 42003_2020_1060_MOESM18_ESM.zip › Additional_file_12/2273.pdf]

Evolution of the gene family "3838" (p=0.036)

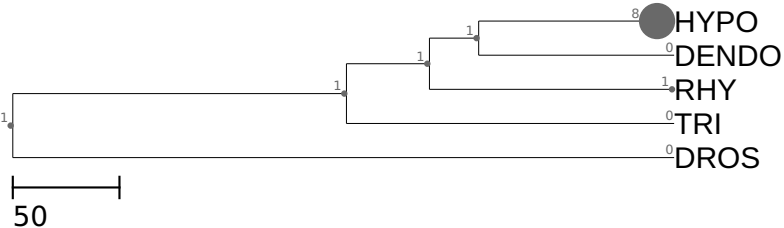

Supplement: Supplementary file 18 — Supplementary data file 13 [file 42003_2020_1060_MOESM18_ESM.zip › Additional_file_12/3838.pdf]

## Evolution of the gene family "1963" ( $p=0.008$ )

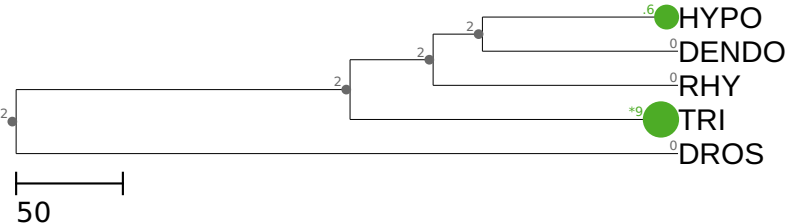

Supplement: Supplementary file 18 — Supplementary data file 13 [file 42003_2020_1060_MOESM18_ESM.zip › Additional_file_12/1963.pdf]

# Evolution of the gene family "213" ( $p=0.025$ )

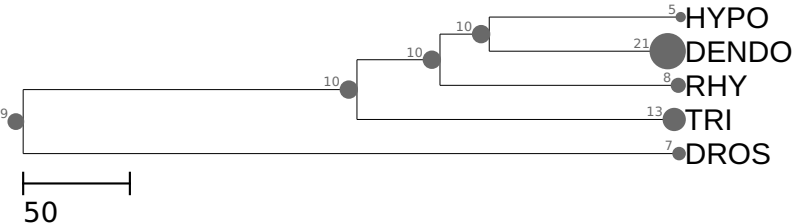

Supplement: Supplementary file 18 — Supplementary data file 13 [file 42003_2020_1060_MOESM18_ESM.zip › Additional_file_12/213.pdf]

# Evolution of the gene family "575" ( $p=0.001$ )

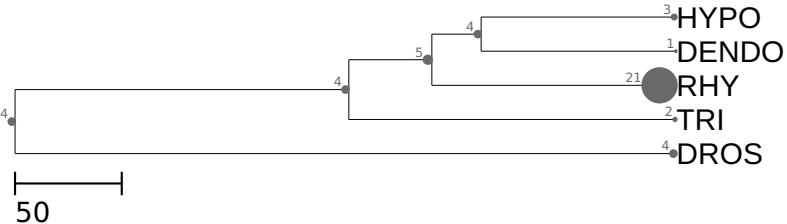

Supplement: Supplementary file 18 — Supplementary data file 13 [file 42003_2020_1060_MOESM18_ESM.zip › Additional_file_12/575.pdf]

# Evolution of the gene family "603" ( $p=0.012$ )

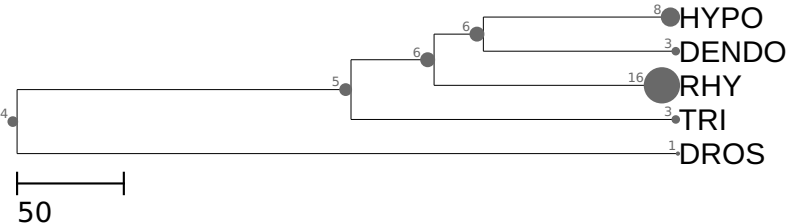

Supplement: Supplementary file 18 — Supplementary data file 13 [file 42003_2020_1060_MOESM18_ESM.zip › Additional_file_12/603.pdf]

# Evolution of the gene family "830" ( $p=0.005$ )

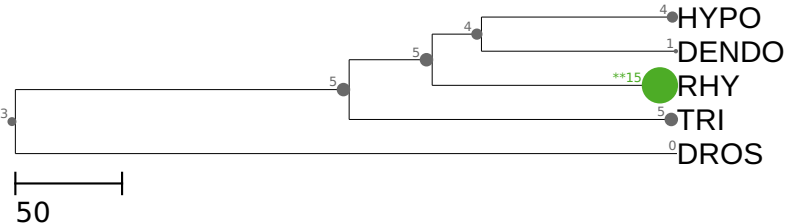

Supplement: Supplementary file 18 — Supplementary data file 13 [file 42003_2020_1060_MOESM18_ESM.zip › Additional_file_12/830.pdf]

Evolution of the gene family "2649" (p=0.023)

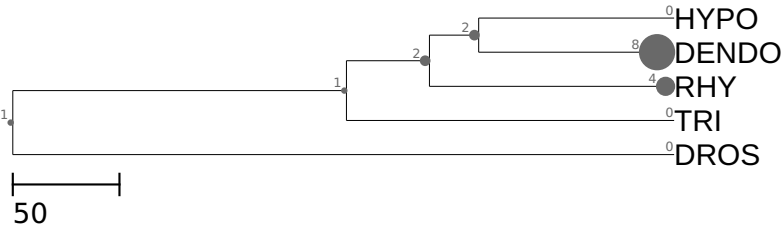

Supplement: Supplementary file 18 — Supplementary data file 13 [file 42003_2020_1060_MOESM18_ESM.zip › Additional_file_12/2649.pdf]

Evolution of the gene family "415" (p=0.001)

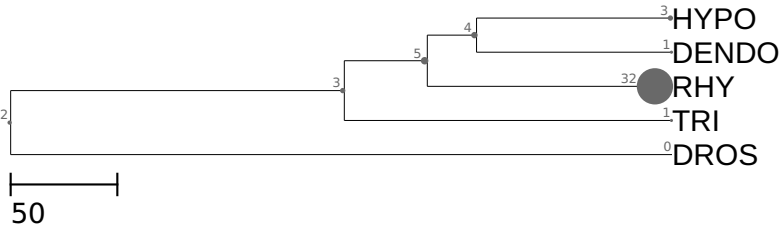

Supplement: Supplementary file 18 — Supplementary data file 13 [file 42003_2020_1060_MOESM18_ESM.zip › Additional_file_12/415.pdf]

# Evolution of the gene family "399" ( $p=0.044$ )

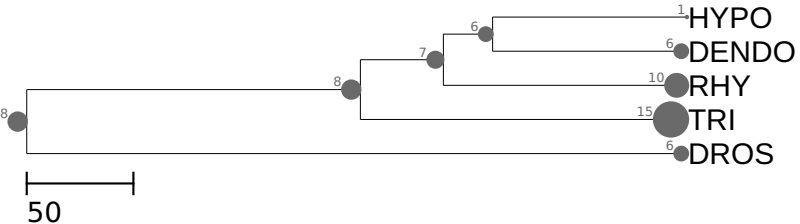

Supplement: Supplementary file 18 — Supplementary data file 13 [file 42003_2020_1060_MOESM18_ESM.zip › Additional_file_12/399.pdf]

Evolution of the gene family "400" (p=0.0)

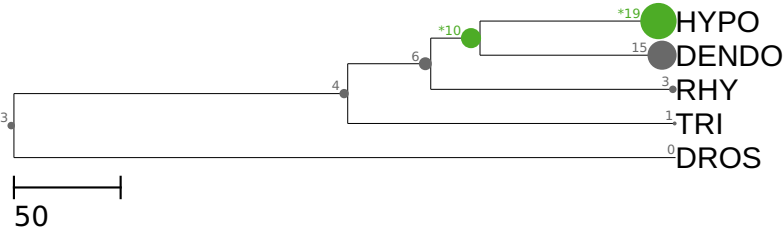

Supplement: Supplementary file 18 — Supplementary data file 13 [file 42003_2020_1060_MOESM18_ESM.zip › Additional_file_12/400.pdf]

Evolution of the gene family "1802" (p=0.013)

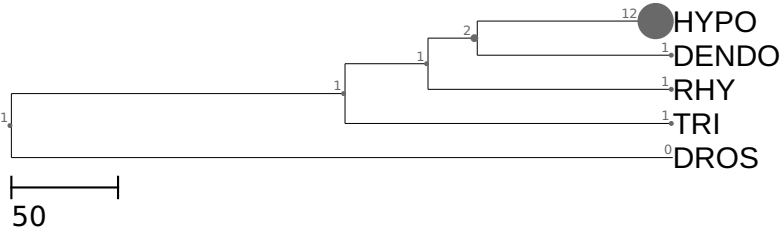

Supplement: Supplementary file 18 — Supplementary data file 13 [file 42003_2020_1060_MOESM18_ESM.zip › Additional_file_12/1802.pdf]

# Evolution of the gene family "819" ( $p=0.004$ )

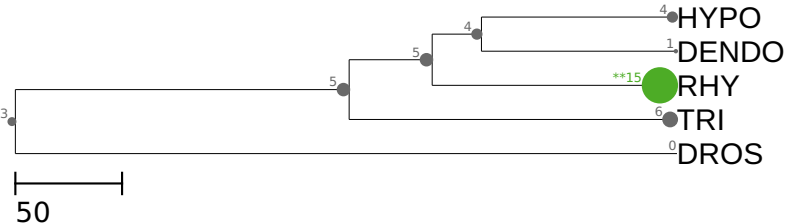

Supplement: Supplementary file 18 — Supplementary data file 13 [file 42003_2020_1060_MOESM18_ESM.zip › Additional_file_12/819.pdf]

Evolution of the gene family "3542" (p=0.036)

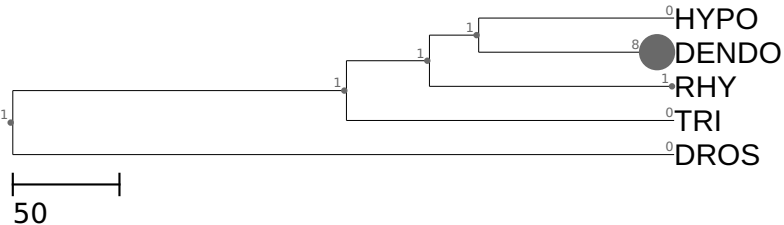

Supplement: Supplementary file 18 — Supplementary data file 13 [file 42003_2020_1060_MOESM18_ESM.zip › Additional_file_12/3542.pdf]

# Evolution of the gene family "65" ( $p=0.004$ )

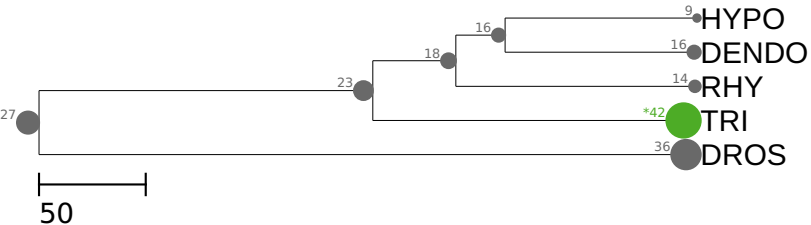

Supplement: Supplementary file 18 — Supplementary data file 13 [file 42003_2020_1060_MOESM18_ESM.zip › Additional_file_12/65.pdf]

# Evolution of the gene family "170" ( $p=0.003$ )

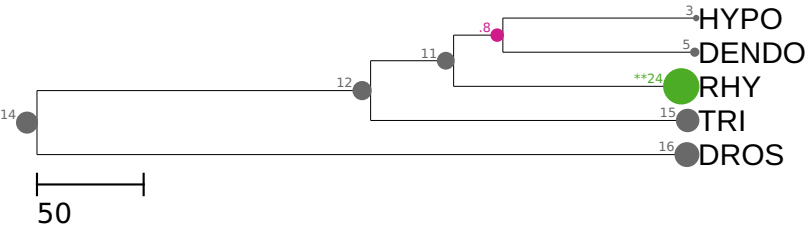

Supplement: Supplementary file 18 — Supplementary data file 13 [file 42003_2020_1060_MOESM18_ESM.zip › Additional_file_12/170.pdf]

Evolution of the gene family "1396" (p=0.002)

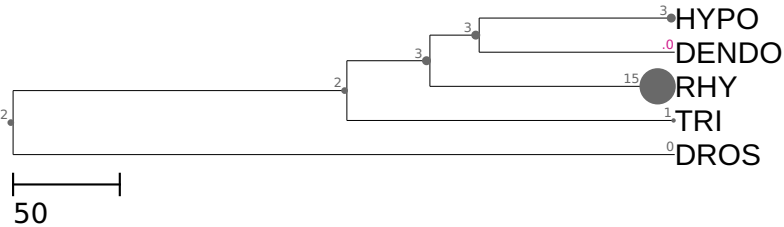

Supplement: Supplementary file 18 — Supplementary data file 13 [file 42003_2020_1060_MOESM18_ESM.zip › Additional_file_12/1396.pdf]

# Evolution of the gene family "158" ( $p=0.041$ )

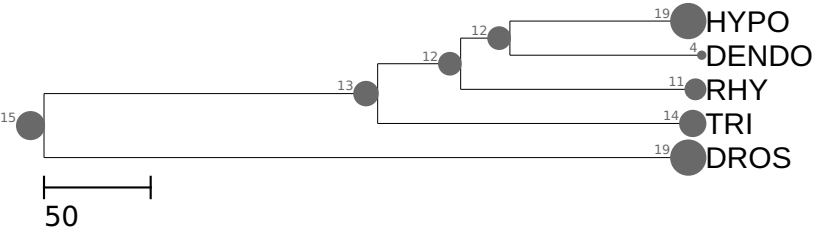

Supplement: Supplementary file 18 — Supplementary data file 13 [file 42003_2020_1060_MOESM18_ESM.zip › Additional_file_12/158.pdf]

# Evolution of the gene family "1235" ( $p=0.03$ )

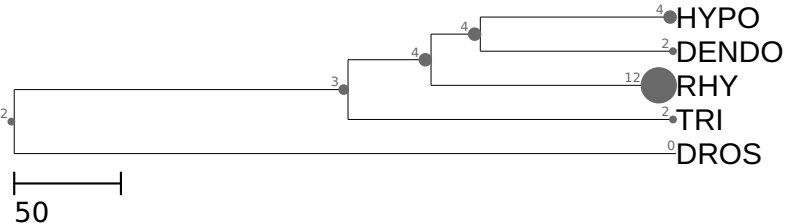

Supplement: Supplementary file 18 — Supplementary data file 13 [file 42003_2020_1060_MOESM18_ESM.zip › Additional_file_12/1235.pdf]

Evolution of the gene family "2728" (p=0.024)

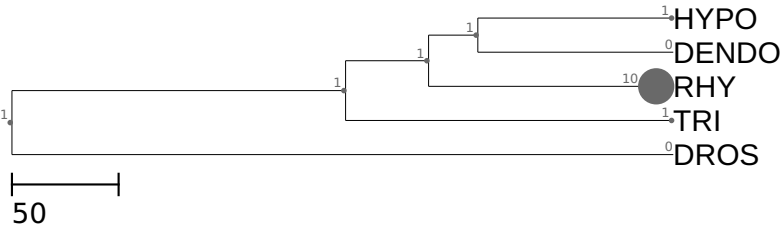

Supplement: Supplementary file 18 — Supplementary data file 13 [file 42003_2020_1060_MOESM18_ESM.zip › Additional_file_12/2728.pdf]

# Evolution of the gene family "1023" ( $p=0.036$ )

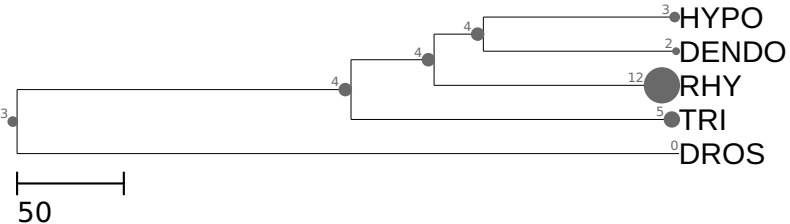

Supplement: Supplementary file 18 — Supplementary data file 13 [file 42003_2020_1060_MOESM18_ESM.zip › Additional_file_12/1023.pdf]

# Evolution of the gene family "212" ( $p=0.002$ )

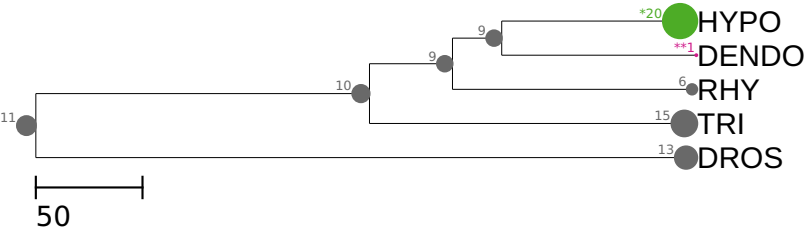

Supplement: Supplementary file 18 — Supplementary data file 13 [file 42003_2020_1060_MOESM18_ESM.zip › Additional_file_12/212.pdf]

# Evolution of the gene family "275" ( $p=0.003$ )

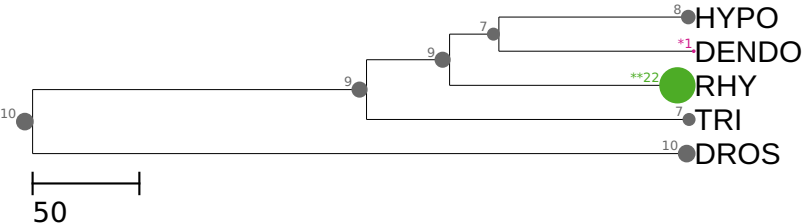

Supplement: Supplementary file 18 — Supplementary data file 13 [file 42003_2020_1060_MOESM18_ESM.zip › Additional_file_12/275.pdf]

Evolution of the gene family "1905" (p=0.003)

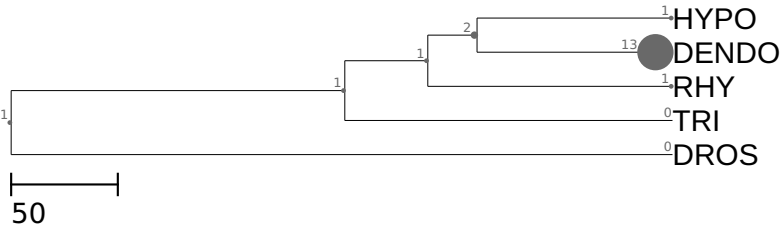

Supplement: Supplementary file 18 — Supplementary data file 13 [file 42003_2020_1060_MOESM18_ESM.zip › Additional_file_12/1905.pdf]

# Evolution of the gene family "117" (p=0.001)

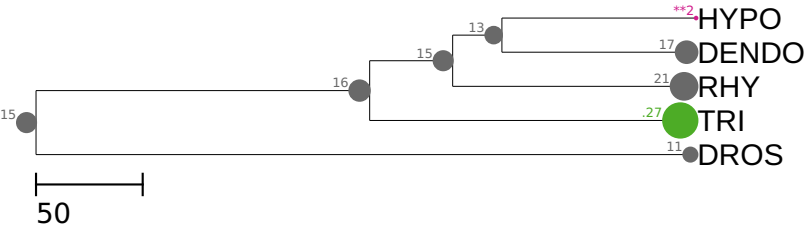

Supplement: Supplementary file 18 — Supplementary data file 13 [file 42003_2020_1060_MOESM18_ESM.zip › Additional_file_12/117.pdf]

Evolution of the gene family "1332" (p=0.017)

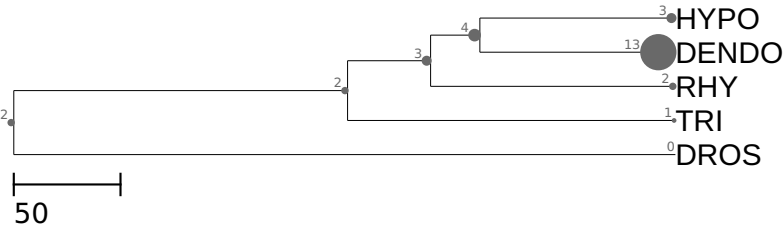

Supplement: Supplementary file 18 — Supplementary data file 13 [file 42003_2020_1060_MOESM18_ESM.zip › Additional_file_12/1332.pdf]

Evolution of the gene family "1440" (p=0.015)

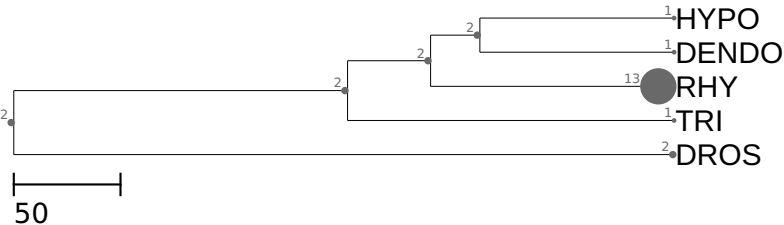

Supplement: Supplementary file 18 — Supplementary data file 13 [file 42003_2020_1060_MOESM18_ESM.zip › Additional_file_12/1440.pdf]

# Evolution of the gene family "1468" ( $p=0.04$ )

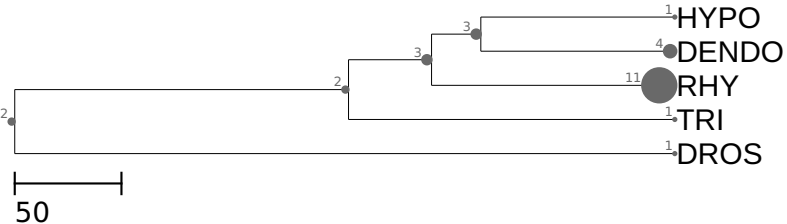

Supplement: Supplementary file 18 — Supplementary data file 13 [file 42003_2020_1060_MOESM18_ESM.zip › Additional_file_12/1468.pdf]

# Evolution of the gene family "473" ( $p=0.025$ )

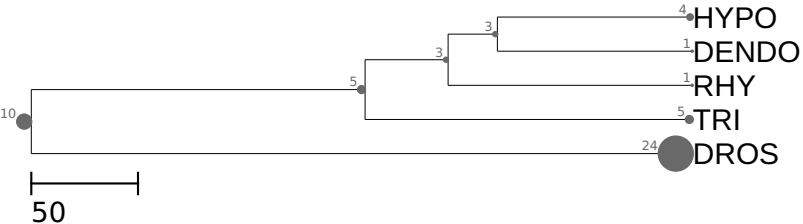

Supplement: Supplementary file 18 — Supplementary data file 13 [file 42003_2020_1060_MOESM18_ESM.zip › Additional_file_12/473.pdf]

Evolution of the gene family "3322" (p=0.031)

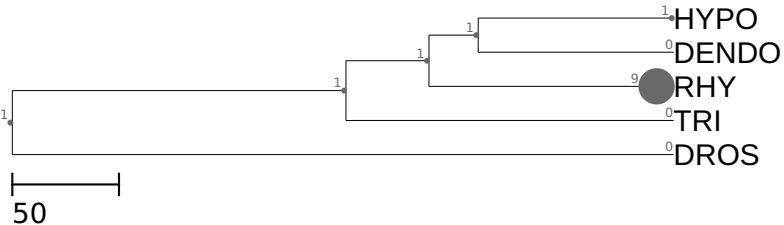

Supplement: Supplementary file 18 — Supplementary data file 13 [file 42003_2020_1060_MOESM18_ESM.zip › Additional_file_12/3322.pdf]

# Evolution of the gene family "248" ( $p=0.001$ )

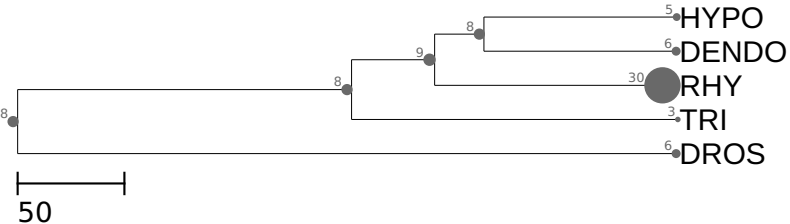

Supplement: Supplementary file 18 — Supplementary data file 13 [file 42003_2020_1060_MOESM18_ESM.zip › Additional_file_12/248.pdf]

# Evolution of the gene family "289" ( $p=0.001$ )

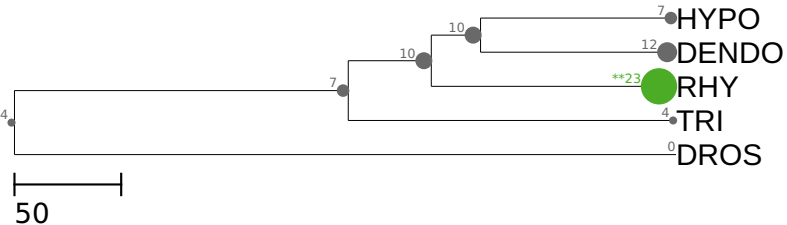

Supplement: Supplementary file 18 — Supplementary data file 13 [file 42003_2020_1060_MOESM18_ESM.zip › Additional_file_12/289.pdf]

# Evolution of the gene family "706" ( $p=0.005$ )

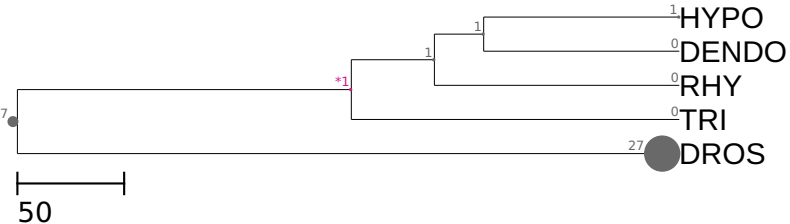

Supplement: Supplementary file 18 — Supplementary data file 13 [file 42003_2020_1060_MOESM18_ESM.zip › Additional_file_12/706.pdf]

# Evolution of the gene family "909" ( $p=0.027$ )

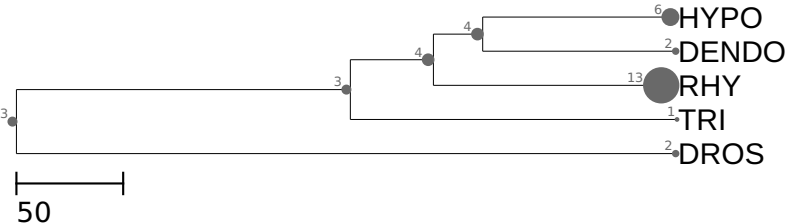

Supplement: Supplementary file 18 — Supplementary data file 13 [file 42003_2020_1060_MOESM18_ESM.zip › Additional_file_12/909.pdf]
