# Supplementary material for: The genome of pest Rhynchophorus ferrugineus reveals gene families important at the plant-beetle interface
Source: Commun Biol. 2020 Jun 24;3:323. doi: 10.1038/s42003-020-1060-8 (PMC7314810; doi:10.1038/s42003-020-1060-8)
Supplement: Supplementary file 20 — Description of Additional Supplementary Files [file 42003_2020_1060_MOESM20_ESM.pdf]

Supplementary data file legend

**Supplementary data file 1** Diversity estimate Pi (π) for red palm weevil (*R. ferrugineus*) using Angsd (http://www.popgen.dk/angsd/index.php/ANGSD)

**Supplementary data file 2** Transposable elements identification among the five species of insects in the study using Repeatmasker software (http://www.repeatmasker.org)

**Supplementary data file 3** Summary of the mitochondrial genes of the red palm weevil (*R. ferrugineus*).

**Supplementary data file 4** Annotation of red palm weevil proteins (*R. ferrugineus)* using NCBI non-redundant database (nr).

**Supplementary data file 5** Annotation of red palm weevil proteins (*R. ferrugineus)* using GO ontology terms.

**Supplementary data file 6** Annotation of red palm weevil proteins (*R. ferrugineus)* using InterProScan database.

**Supplementary data file 7** PFAM protein domain annotation of red palm weevil (*R. ferrugineus)* from InterProScan.

**Supplementary data file 8** KEGG pathway annotation of red palm weevil (*R. ferrugineus)*

**Supplementary data file 9** Enrichment results of red palm weevil proteins (*R. ferrugineus)* using 'Molecular Function from Gene Ontology (GO)', created by dcGO Enrichment.

**Supplementary data file 10** Comparative analysis of protein and signaling pathway of the five species in the study.

**Supplementary data file 11** Expanded gene families from CAFÉ software (https://github.com/hahnlab/CAFÉ).

**Supplementary data file 12** Contracted gene families from CAFÉ software (https://github.com/hahnlab/CAFÉ).

**Supplementary data file 13** Zipped file including expanded and contracted gene families phylogenetic trees.

**Supplementary data file 14** Comparative analysis of PBP/GOBP families of the five species in the study.

**Supplementary data file 15** Summary of structural variation, duplication, deletion, inversion, tandem duplication of the five species in the study.

**Supplementary data file 16** Total numbers of structural variations (SVs) of the five species in the study.

**Supplementary data file 17** Parameter estimates and likelihood scores for transient receptor potential ion channels (Melastatin) (TRPM) genes under models of variable ω ratios.

**Supplementary data file 18** Zipped files of LastZ alignments of red palm weevil (*R. ferrugineus)* against red flour beetle (*T.castaneum*)
